# Utilizing an Amino Acid Scaffold to Construct Heteroditopic Receptors Capable of Interacting with Salts under Interfacial Conditions

**DOI:** 10.3390/ijms221910754

**Published:** 2021-10-05

**Authors:** Damian Jagleniec, Natalia Walczak, Łukasz Dobrzycki, Jan Romański

**Affiliations:** Faculty of Chemistry, University of Warsaw, Pasteura 1, 02-093 Warsaw, Poland; djagleniec@chem.uw.edu.pl (D.J.); nataliachorzewska97@gmail.com (N.W.); dobrzyc@chem.uw.pl (Ł.D.)

**Keywords:** ion pair recognition, 4-nitro-L-phenylalanine, squaramide, crown ether, anion transport

## Abstract

A 4-nitro-L-phenylalanine scaffold was used to construct effective ion pair receptors capable of binding anions in an enhanced manner with the assistance of alkali metal cations. A benzocrown ether was linked to a receptor platform *via* the amide function so as to support the squaramide function in anion binding and to allow all three NHs to act simultaneously. The binding properties of the receptors were determined using UV-vis, ^1^H NMR, 2D NMR, and DOSY spectroscopy in MeCN and in the solid state by X-ray measurements. Ion pair receptor **2** was found to interact with the most strongly with salts, and the removal of its key structural elements was shown to hinder the receptor action. The amide proton was recognized to switch from having involvement in an intramolecular hydrogen bond to interacting with anions upon complexation. Apart from carboxylates, which promote deprotonation, and other monovalent salts creating 1:1 complexes with the receptor, more complex equilibria were established upon the complexation of **2** with sulfates. Receptor **2** was shown to be capable of the extraction of ion pairs from the aqueous to organic phase and of the cation-enhanced transport chloride and sulfate anions across a bulk chloroform membrane. These features may open the door for its use in regulating ion concertation under interfacial conditions and acting as a potential drug to treat channelopathies.

## 1. Introduction

**Figure 1:**
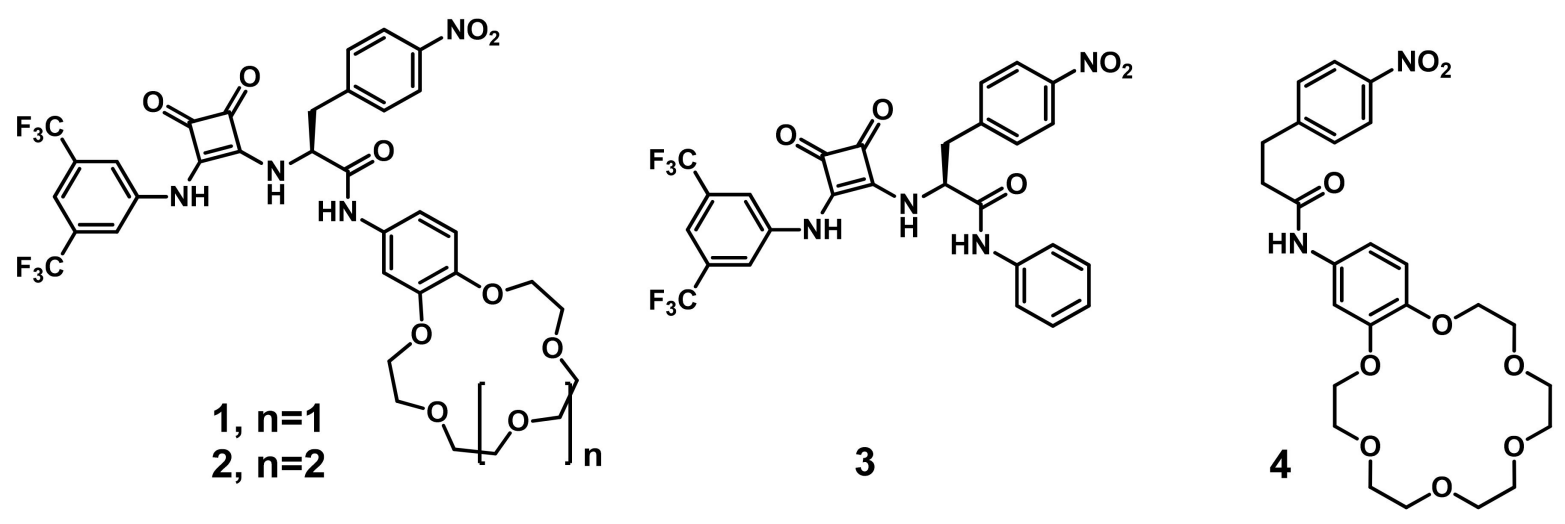
Structures of receptors **1**–**4**.

There has been significant progress in the design of molecular anion receptors characterized by high selectivity and efficiency in recent years. The use of anion binding domains tethered to appropriate molecular platforms has allowed a number of molecular receptors to be obtained, with applications in medicine, environmental remediation, and many industrial processes [1,2,3,4]. In particular, modifying anion receptors with a signaling unit has led to selective sensors [5,6,7,8,9]; incorporating strong and/or multiple binding domains into the receptor scaffold results in effective extractants that are able to operate in both solid–liquid and liquid–liquid conditions [10]; carefully balancing the strength of the receptor binding domains gives rise to transmembrane transporters [11,12,13,14]; and harnessing the interaction of the binding site with particular anions opens up the possibility of them acting as organocatalysts [15,16,17]. However, in some processes, the presence of strongly coordinating cations may hamper the action of monotopic anion receptors, as competitive ion pairing occurs [18,19]. A solution to this problem may lie in designing heteroditopic ion pair receptors which, possessing both cation and anion binding domains, are capable of binding ion pairs simultaneously [20,21,22,23]. Apart from the selection of binding sites, their proper arrangement on the molecular platform is important from the point of view of cooperativity in ion binding, which translates directly into the effectiveness of the receptors [24,25,26,27]. Thus, choosing an appropriate molecular platform is crucial in the design of molecular receptors, and it has repeatedly been shown that amino acids are convenient candidates for such a purpose. The presence of both the amino and carboxylate groups in their structure allows the binding domains to be introduced in a relatively simple way [28]. In addition, the existence of additional functional groups in the side chains of amino acids significantly expands the scope of their use in molecular recognition [29,30]. By treating amino acids as building blocks and by combining them together, it has been shown that acyclic or cyclic peptides can be obtained as monotopic anion receptors [28,31]. The pioneering work by Kubik demonstrated that cyclopeptides are able to recognize anions effectively, even in an aqueous environment [32,33,34,35]. On the other hand, dipeptides, cyclopeptides, and amino acid-based squaramides have been utilized in sulfate recognition [36,37,38]. An L-valine-based ion pair receptor was found to effectively recognize potassium carboxylates and extract them from the aqueous phase to the organic phase [39]. The advantages of peptide derivatives were also found to be utilized in the transmembrane transport of chloride anions [40,41,42,43,44] and in rare examples of sulfate anion transport by molecular receptors have also been reported [45,46]. In fact, effective transporters for both of these anions are highly desired. Appropriate amounts of sulfates assure the proper function of the kidneys, liver, lungs, intestines, brain, and various other organs in our bodies [47,48]. On the other hand, chloride anions are responsible for maintaining the acid–base balance, muscular activity, osmotic pressure, and water balance of the human body [49,50]. For this reason, chloride receptors and transporters could prove to be very effective in the treatment of numerous channelopathies resulting from the disturbance of their intercellular transport, such as cystic fibrosis [13]. Recently, it has also been shown that monotopic receptors capable of transporting chlorides can be treated as a potential anti-cancer therapy [51]. Our own contribution to the field of receptors that are able to interact with chloride anions under interfacial conditions has resulted in a series of amino acid-based ion pair receptors [52,53]. Nevertheless, all of our attempts to utilize them to extract salt from the aqueous phase to the organic phase failed; we found that only a receptor possessing squaramide units and an acylated aza-18-crown-ether unit as a cation-binding site could extract chloride anions, albeit with the assistance of bulky cations [54]. Therefore, we decided to revise our approach to the construction of amino acid-based ion pair receptors by keeping the squaramide unit, which has been established as strongly interacting with anions, and changing the type of cation-binding domain and the way in which it is linked [55,56,57]. We envisioned that by introducing a benzocrown unit [58] as a cation-binding domain tethered to the amino acid platform via an amide bond, we would be able obtain effective ion pair receptors that are able to operate under interfacial conditions and to transport anions across a membrane in an enhanced manner by utilizing the symport with the cations [46,59,60]. The following assumptions support this idea: the additional amide function should support the squaramide unit in anion binding, the presence of a cation-binding domain opens up a way to bind cations and anions cooperatively, and the side chain of the amino acid group should assure the lipophilic nature of the receptors required for effective transport across a membrane. To verify this hypothesis, 4-nitro-phenylalanine-based ion pair receptors were designed, and their binding properties were compared to those of an monotopic anion receptor or ion pair receptor lacking a squaramide unit (Figure 1).

## 2. Results and Discussion

**Scheme 1:**
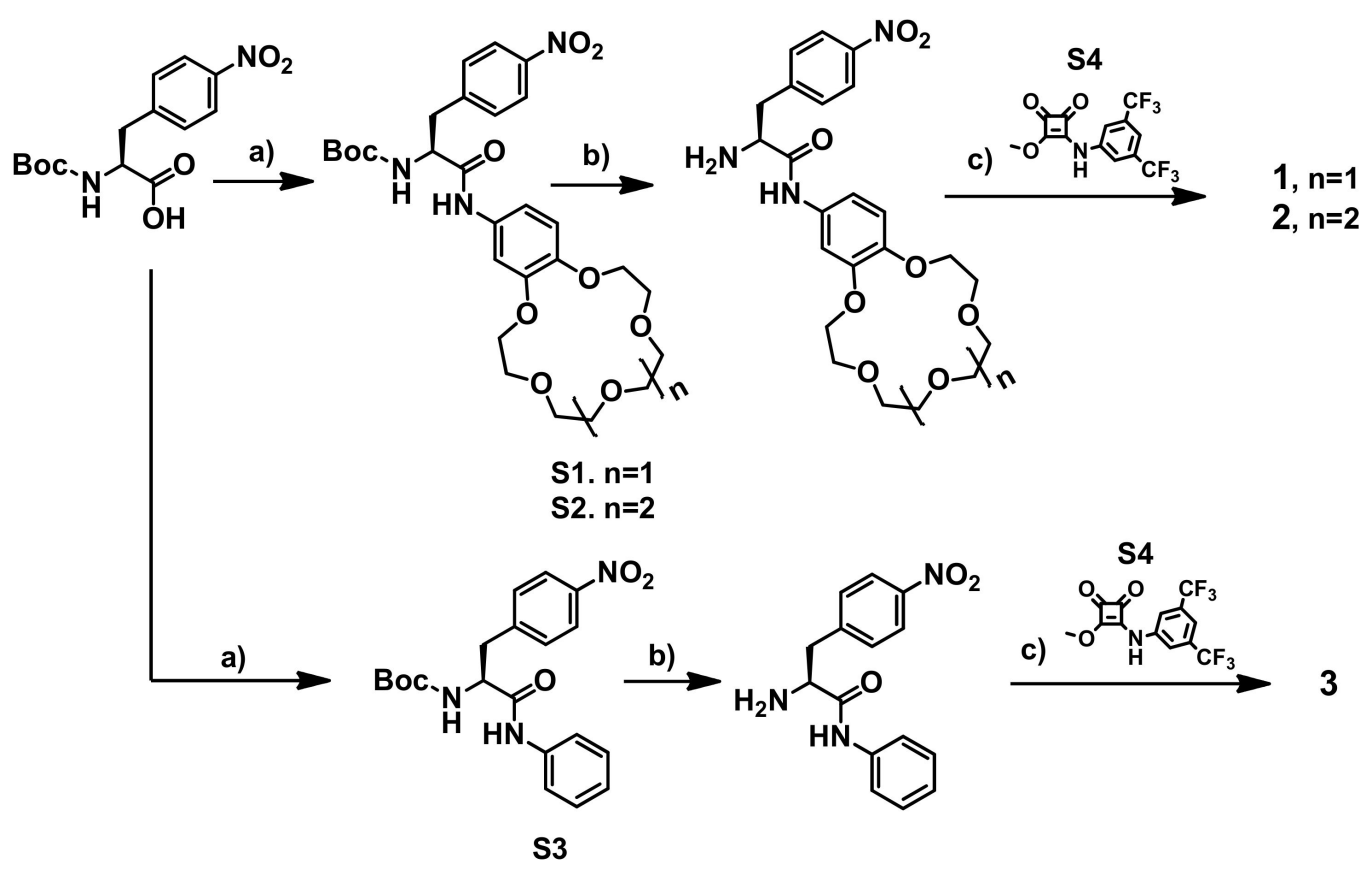
Synthesis of receptors **1**–**3**. Reagents and conditions: (**a**) CH_2_Cl_2_, DCC, 4-aminobenzo-15-crown-5, 4-aminobenzo-18-crown-6 or aniline, 0 °C to r.t., 24 h, 68% (**S1**), 90% (**S2**), 91% (**S3**); (**b**) TFA-CH_2_Cl_2_ (1:4), r.t., then NaHCO_3_; (**c**) triethylamine, **S4**, MeOH, 24 h, r.t., 70% (**1**), 65% (**2**) and 57% (**3**).

**Scheme 2:**
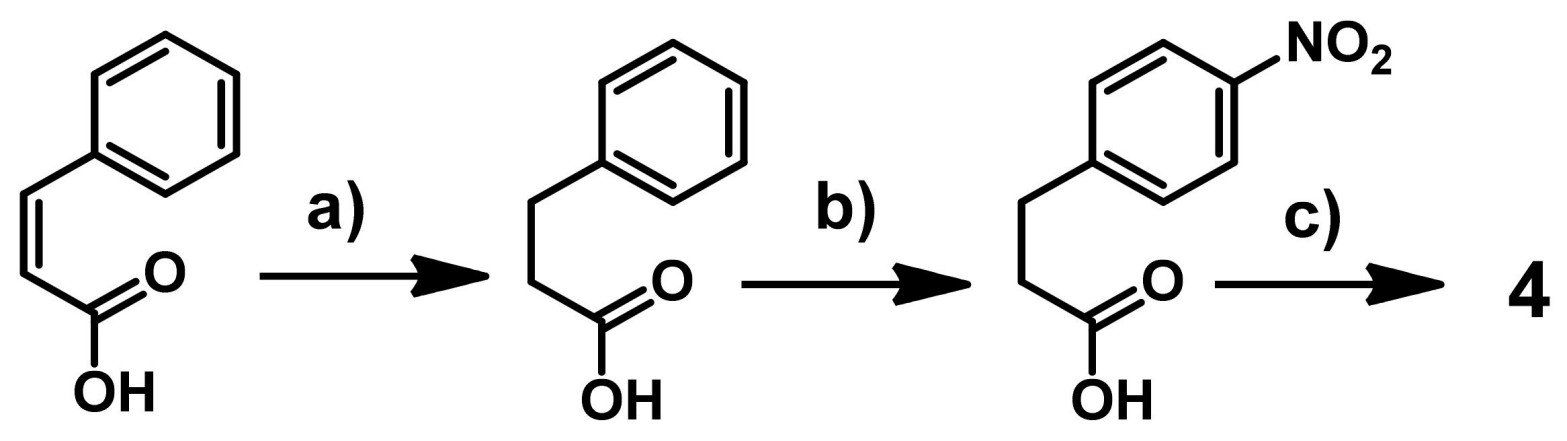
Synthesis of receptor **4**. Reagents and conditions: (**a**) H_2_, Pd/C, MeOH/THF, 12 h, r.t., quantitative; (**b**) H_2_SO_4_:HNO_3_ (1:2, *v*/*v*), CHCl_3_, 0 °C, 33%; (**c**) CH_2_Cl_2_, DCC, 4-aminobenzo-18-crown-6, 0 °C to r.t., 24 h, 53%.

Ion pair receptors **1** and **2** as well as monotopic anion receptor **3** were synthesized in four steps, starting from commercially available Boc-4-nitro-L-phenylalanine, as outlined in Scheme 1. Briefly, the DCC-promoted amide bond formation allowed benzocrown ethers or phenyl units to be introduced to the amino acid scaffold. The acid-mediated deprotection of the 4-nitro-L-phenylalanine derivative yielded the corresponding amines. These amines were used in the second step of the sequential amidation of dimethyl squarate after its reaction with 3,5-trifluoromethylaniline, providing receptors **1**–**3**. Reference receptor **4** was synthesized from cinnamic acid through the heterogenous hydrogenation of the double bond followed by the nitration of the phenyl group, which resulted in a mixture of *orto*- and *para*- isomers of 3-(nitrophenyl) propionic acid with a ratio of 25:75. The 3-(4-nitrophenyl) propionic acid could be separated by means of simple crystallization from a hexane:ethyl acetate mixture and was used in DCC-mediated coupling with 4-aminobenzo-18-crown-6 to yield receptor **4** (Scheme 2).

With all four receptors in hand, we investigated their binding properties towards different anions (tested as tetrabutylammonium salts, TBAX) and in situ generated ion pairs (mixtures of TBAX and sodium perchlorate or potassium hexafluorophosphate) in acetonitrile by means of UV-vis titration measurements. The dilution experiments excluded the self-association of **1**–**3** in the investigated concentration range. First, we compared the affinity of the receptors towards chloride anions in the absence and presence of sodium and potassium cations. An inspection of Table 1 shows that ion pair receptors **1** and **2** interact with chloride anions similarly and more strongly than anion receptor **3** does. This was contrary to expectations, according to which anion receptor **3**, which lacks two alkoxy donating groups on the phenyl ring nearby the amide function (as is in the structure of **1** or **2**), should interact more tightly with anions. This discrepancy suggests that the enhancement of anion binding by means of an additional amide function as a support for the squaramide anion-binding domain may be affected by other factors such as the formation of intramolecular hydrogen bonds. Due to the lack of electron donating groups in receptor **3′**s structure, the participation of amide function in the plausible formation of intramolecular hydrogen bonds with the squaramide oxygen atom is greater, reducing its ability to interact with anions. This conclusion is supported by the NMR measurements, which are discussed later in this paper. As expected, both ion pair receptors **1** and **2** could recognize chloride anions in an enhanced manner with the assistance of cations, with over twofold enhancement found for the recognition of chloride anions by receptor **2** in the presence of potassium cations. Monotopic anion receptor **3** is devoid of this feature, and the presence of cations hampers its action. Bidentate ligand **S2**, on the other hand, forms complexes with chlorides with apparent association constants that are two magnitudes of order weaker than they are for tridentate receptors **1**–**3**.

Based on the initial screening and consideration of the highest effectivity of **2** in anion and ion pair binding, extended binding studies were conducted for this receptor. A comparison of apparent association constants, summarized in Table 2, showed that receptor **2** binds the investigated anions in the following order: Cl^−^ > NO_2_^−^ > Br^−^ > NO_3_^−^. In the case of the titration experiments performed with carboxylate anions, partial deprotonation of receptor **2** was taken into consideration. Specifically, upon the titration of **2** with these anions, a new bathochromic band appeared, and the color of the solution changed to yellow. Similar behavior was noted for the interaction of **2** with tetrabutylammonium hydroxide, although the spectral changes were more pronounced (Figures S27–S29). For each of the qualitatively tested anions, binding enhancement was found to occur with the assistance of sodium cations, and a further increase in binding strength was noted for the interaction of **2** with in situ generated potassium salts. The obtained results clearly show the high affinity of **2** towards chlorides. Thus, we examined the ability of **2** to recognize chloride salts in more competitive, aqueous media. Specifically, we conducted titration experiments in acetonitrile with 0.5, 1, and 5% water and found that the presence of sodium or potassium cations still enhanced chloride anion binding by receptor **2** (Table S1, Figures S30–S37). The presence of 5% water in acetonitrile created an opportunity to test sodium and potassium chloride directly instead of in salts generated in situ. The association constants for the interaction of NaCl with **2** was too low to be precisely determined by the UV-vis technique (K_NaCl_ = 954 M^−1^ was assumed); however, in the case of interaction with potassium chloride, the association constant was calculated to be K_KCl_ = 1425 M^−1^. This indicates that receptor **2** is able to create stable complexes with KCl, even in highly competitive aqueous media. On the other hand, the data collected from the titration of **2** with sulfate salts in acetonitrile could not be fitted to the appropriate binding model. The binding isotherm thus obtained shows a multistep profile, suggesting more complex equilibria (Figures S38–S40). 

**Figure 2:**
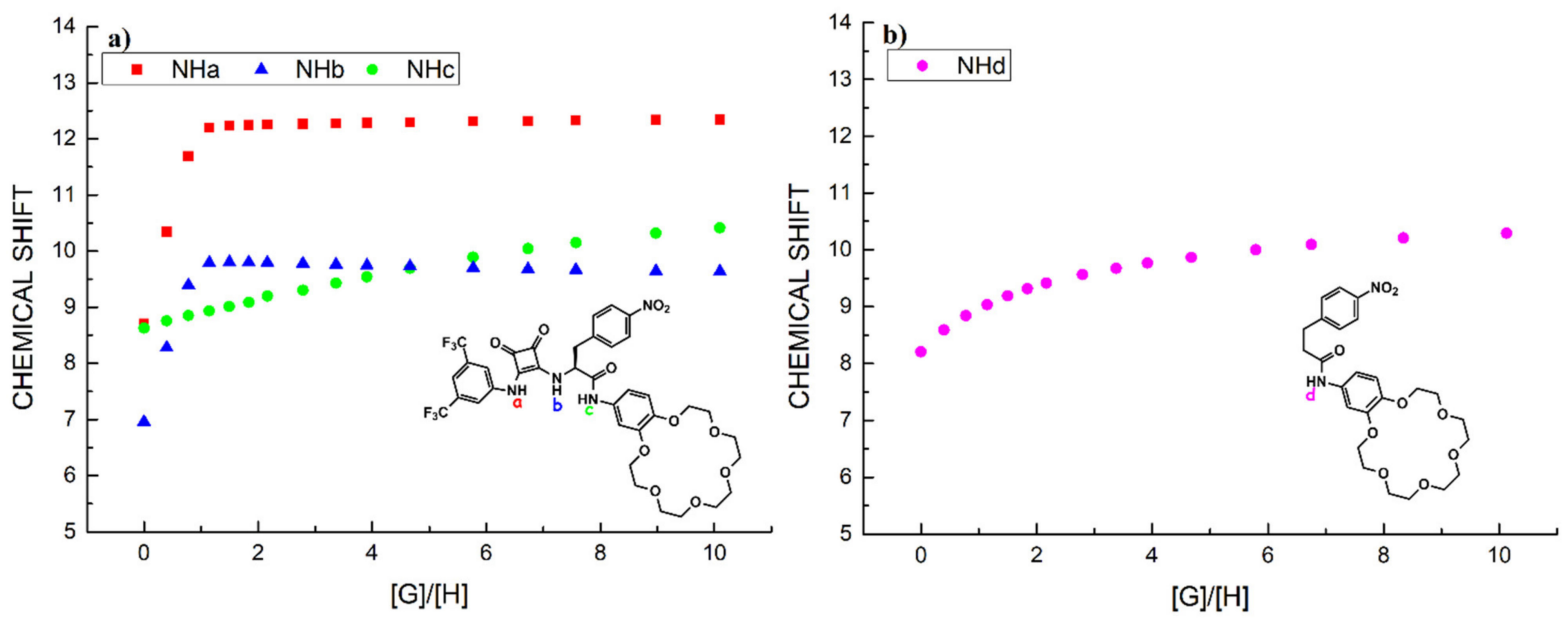
Titration curves obtained by following squaramide and amide protons upon incremental addition of TBACl into solution of (**a**) **2** and (**b**) **4** in CD_3_CN.

Better insight into the binding mechanism of the synthesized receptors came from the ^1^H NMR measurements carried out in CD_3_CN. Two-dimensional NMR experiments were also performed for receptor **2** to assign signals corresponding to specific functional groups that could be involved in complexation (Figures S47–S48). Dilution experiments ruled out possible intermolecular interaction in the investigated concentration range. Interestingly, we noticed that in the case of **2**, the signal corresponding to the amide proton shifted downfield and resonated at δ = 8.62 ppm. An even more pronounced shift (δ = 8.79 ppm) was noted for anion receptor **3**, which was lacking a crown ether unit *viz.* an electron donating group. This suggests that in both cases, an intramolecular hydrogen bond involving the proton of the amide (NH_Am_) and possibly one of the oxygen atoms of the squaramide unit should be taken into account. Its more prominent participation in the case of monotopic receptor **3** (due to the lack of two donating alkoxy substituents on phenyl ring) explains the aforementioned findings, i.e., the weaker interaction of **3** with different anions than in the case of receptor **2**. To support this conclusion, the ^1^H NMR spectrum of receptor **4** was analyzed. Indeed, the removal of the squaramide function, as in the structure of receptor **4**, was reflected in the change of the position of the signal corresponding to the amide proton in the ^1^H NMR spectrum to δ = 8.16 ppm, which was most likely the result of the inability of intramolecular hydrogen to undergo bond formation (Figure 2b). Further analyses of the binding properties of **4** led to other important conclusions. Although the cooperation in ion pair binding was retained (K_TBACl_= 76 M^−1^ vs. K_KCl_= 196 M^−1^), the lack of a squaramide function substantially decreased the ability of **4** to recognize anions and ion pairs in reference to receptors **1** or **2** (Figures S49–S52). The picture emerging from these findings could contradict our idea of designing of ion pair receptors utilizing an amide function as a support to the squaramide-binding domain since this function is involved in intramolecular binding. Inspection of the ^1^H NMR spectra collected from the titration of **2** with chloride salts, however, showed that all three hydrogen bond (Figure 2a, Figure S53) donors may interact with anions simultaneously at the expense of breaking their intramolecular hydrogen bond. Specifically, with up to approximately 1.5 equivalents of chloride anions added to the solution of **2**, a substantial downfield shift of both squaramide protons was noted, with Δδ = 3.50 and 2.85 ppm for NH_a_ and NH_b_, respectively. At the same time the NH_c_ signal was shifted only by Δδ = 0.31 ppm. This may be rationalized in terms of the strong interaction of both squaramide protons with anions and the participation of amide protons (NH_c_) switching from intramolecular hydrogen bonding to interaction with anions. Such conclusion was supported by 2D ROESY measurements (Figures S47 and S55). Specifically, the analysis conducted for the solution of **2** in deuterated acetonitrile shows no correlation of the NH_c_ proton with those belonging to the squaramide unit NH_b_. After the addition of chloride anions, such a correlation did appear, which confirms the close proximity of both protons and supports our findings regarding the simultaneous interaction of all three NHs with anions. The further addition of chloride anions (up to 10 equivalents) caused a consistent, only slight downfield shift of NH_a_ (by Δδ = 0.14 ppm), while NH_b_ was shifted back and slightly upfield (by Δδ = 0.15 ppm) together with a considerable downfield shift of NH_c_ by Δδ = 1.77 ppm. Changes in the ^1^H NMR spectra during titration were also observed for other signals, with greater alterations in those nearby the anion binding sites. The continuous shift of NHc up to the end of titration may suggest that the excess of chloride anions invoked the change in the binding mode comprising the participation of the complexes with 1:2 (host:guest) stoichiometry. 

Receptor **2** exhibited similar behavior when titration with chloride anions was conducted with the assistance of potassium cations. Due to the switching of the amide proton from participation in the formation of intramolecular hydrogen bonds to interaction with anions, we could not fit the obtained ^1^H NMR titration data to an appropriate model and calculate a stability constant using the ^1^H NMR technique. 

Another picture emerged from the titration of **2** with sulfate anions (added as TBA salt) and indicates more complex equilibria (Figures S59 and S60). Specifically, upon the incremental addition of sulfate anions, all three NHs were shifted consistently downfield for up to 2.64 equivalents of anions added, together with consistent signal shifts corresponding to almost all of the other protons. Interestingly, despite the absence of strongly coordinating cations in the system, the changes were also evident in the signals corresponding to crown ether protons. This may be attributed to the formation of complexes with higher stoichiometry, which requires ligands to be tightly packed around the sulfate anion. Specifically, the signals corresponding to the crown ether were initially shifted upfield. Upon the addition of approximately 3.40 equivalents of sulfate anions, new sets of signals appeared, enabling different types of complexes of **2** with sulfates to be distinguished in the NMR timescale. This also applied to crown ether protons, with the appearance of a new set of signals that were shifted downfield. After this point was exceeded, only new signals remained, and they did not change their position until the end of the titration, most likely due to formation of 1:1 complexes (**2** ⸦ SO_4_^2−^). 

To better understand the binding mode of **2** with sulfates, DOSY and ROESY experiments were applied. The diffusion coefficient measured for **2** in acetonitrile was found to be D = 0.946 × 10^−9^ m^2^ s^−1^ and dropped after the addition of 5 equivalents of sulfate anions added to D = 0.849 × 10^−9^ m^2^ s^−1^. Interestingly, we were able to measure the diffusion coefficients based on the resolved signals at the point where 3.40 equivalents of sulfate anions were added to the solution of **2**. However, we found that these two values are comparable to each other, with D = 0.807 × 10^−9^ m^2^ s^−1^ and D = 0.759 ×10^−9^ m^2^ s^−1^, respectively. On the one hand, their values suggest the existence of a complex with higher stoichiometry in solution, while on the other hand, they do not evidently differentiate these species. The ROESY experiments confirmed the rapid exchange between these complexes, which explains the similar values of the diffusion coefficients measured at this point and shows that these values should be treated as being averaged. Indeed, when the diffusion coefficient was measured after the addition of one equivalent sulfate anions to receptor **2**, where only one set of signals appeared, a lower diffusion coefficient was found, with D = 0.756 × 10^−9^ m^2^ s^−1^. This suggests that the complexes with higher stoichiometry had the highest mol fraction at the beginning of titration. 

To analyze the ability of **2** to form crystalline complexes and to obtain the receptor structure in the solid state, crystallization with **2** and KBr in acetonitrile media was performed. However, the resulting crystals also contained NaBr species—surprisingly, as a major component. The source of the Na^+^ cations can be attributed to the glassware used during the synthesis and crystallization experiments. The obtained crystals belong to the *P*$\bar{1}$ space group, with an asymmetric part being composed of receptor **2**, NaBr/KBr salt with a non-stoichiometric number of cations, a Na^+^/K^+^ ratio yielding 0.866/0.134, and partial occupancy water moiety and one acetonitrile molecule. Such a mixed salt structure results in the disorder of the moieties in the crystal lattice. Both the partial occupancy Na^+^ and K^+^ cations are of course coordinated by the crown ether ring. However, the slightly too small sodium moiety causes the ring to fold somewhat around the cation. The crown ether part is also corrugated when coordinating K^+^ species; however, in the case of this cation, the complexation is partly external. 

**Figure 3:**
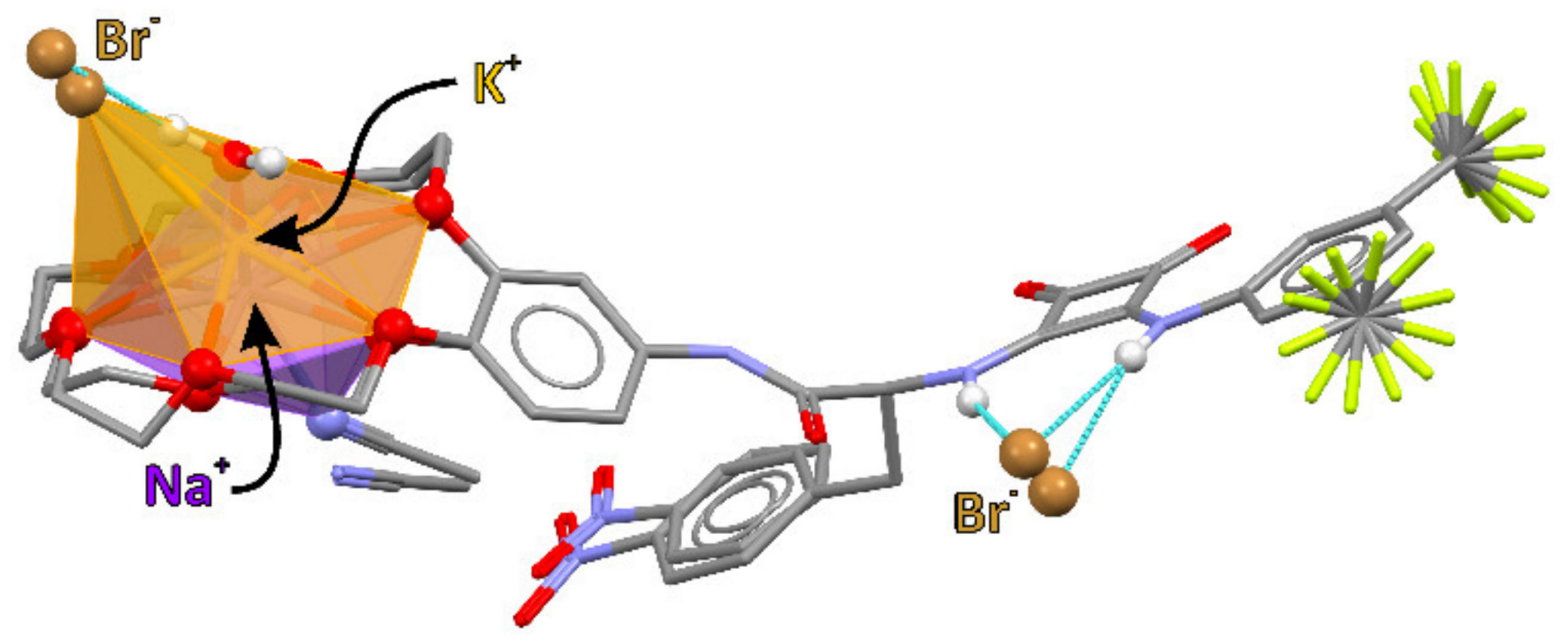
Structure of receptor **2** in a complex with KBr/NaBr salts presenting the coordination of the cations. All N-H and C-H hydrogen atoms are omitted for clarity.

This different manner of cation coordination leads to disorder in the aliphatic part of the crown ether moiety, as the occupancy is related to the Na^+^/K^+^ ratio. The smaller Na^+^ cation located inside the crown ether ring is coordinated from both sides by additional ligands: the N-end of the acetonitrile molecule and the O atom of the water molecule. Four distances from the crown ether O atoms to Na^+^ are significantly shorter than two others (2.60–2.67 Å versus 2.88–2.94 Å); however, the acetonitrile and water moieties coordinate the cation even more strongly, which is especially visible in the case of interaction with H_2_O (2.40 Å and 2.27 Å for Na^+^···N and Na^+^···O distances, respectively). The partial occupancy water molecule interacts via a hydrogen bond with a Br^−^ anion (O···Br^−^ distance equal to 3.31 Å) that is bound by the squaramide N-H moieties of a neighboring receptor (N···Br^−^ distances yielding 3.44 Å and 3.26 Å for the N2 and N3 amide groups, respectively). All the acetonitrile, water, and Br^−^ moieties have the same occupancy as the sodium cation, i.e., 0.866. When the K^+^ moiety is coordinated instead, the water molecule is absent (thus, its amount in the unit cell is non-stoichiometric), but from the same site, the Br^−^ anion acts as a ligand. Of course, the position of the bromide anion coordinating the K^+^ ion is different when the Na^+^ cation is present, resulting in static disorder. Nonetheless, the total number of anions per one receptor is equal exactly one. The alternatively located Br^−^ anion is also coordinated by squaramide N-H groups, with the N···Br^−^ distances equal to 3.56 Å and 3.42 Å for the N2 and N3 amide groups, respectively. Once the K^+^ is externally bound by the crown ether, the space around the concave site of the folded crown ether moiety is filled by an acetonitrile molecule with occupancy yielding 0.134, only acting as a free solvent that does not coordinate with the potassium ion. The potassium ion is also not symmetrically coordinated by the crown ether oxygen moieties, but the difference in the distances is less prominent than it is in the case of sodium cation. Here, there are four shorter distances ranging from 2.71 Å to 2.79 Å, and two longer ones that are 2.92 Å and 2.98 Å. The K^+^···Br^−^ distance is of course longer and is equal to 3.39 Å. Similar to the case of the Br^−^ ion, the acetonitrile molecule is statically disordered over two positions. In addition, some disorder of the nitrobenzene part and the CF_3_ groups can also be observed. In spite of the disorder of both the cationic domain and the anion, the squaramide-binding site is fully ordered. This slightly complicated, mixed site and disordered structure is visualized in Figure 3.

**Figure 4:**
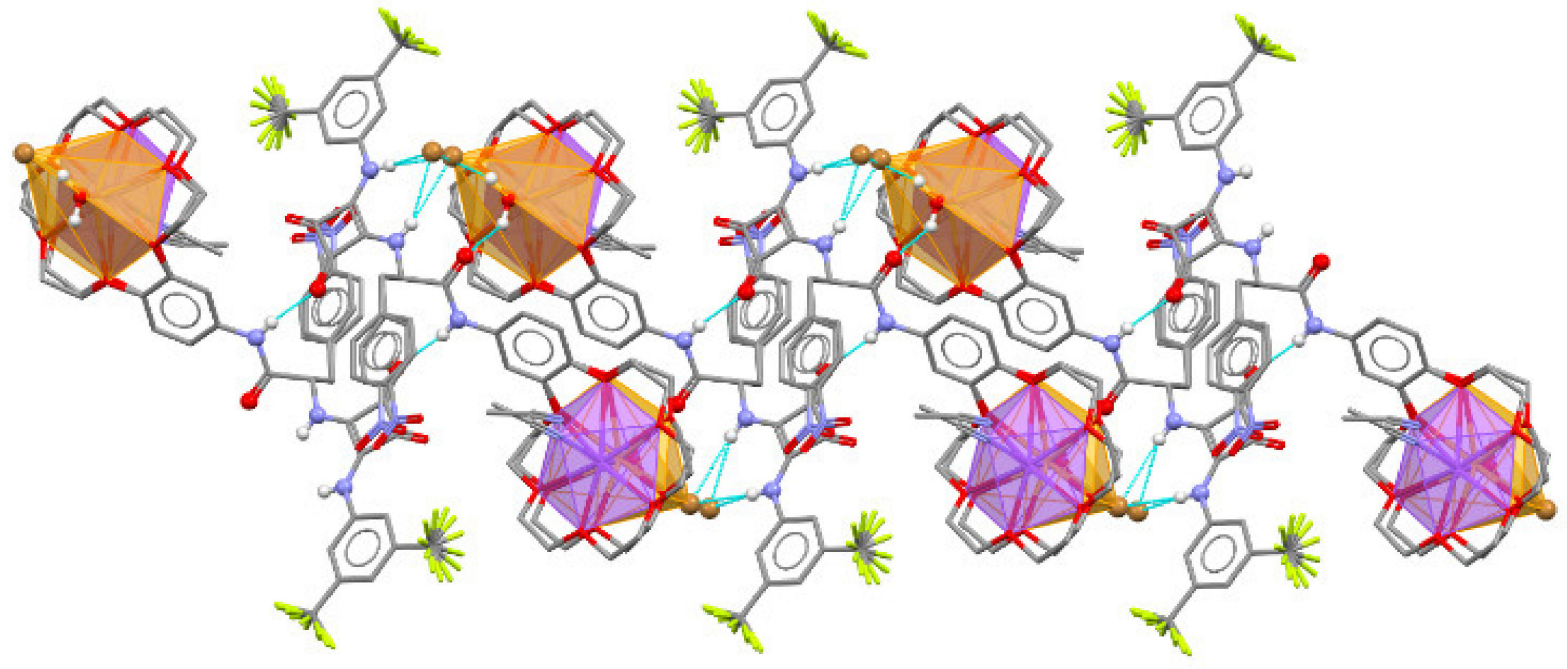
Intermolecular interaction and formation of 1-D supramolecular polymer in the structure of **2** with KBr/NaBr salts. All N-H and C-H hydrogen atoms are omitted for clarity.

In the structure, only the squaramide N-H groups are involved in bromide anion binding. The third amide moiety is engaged in an intermolecular hydrogen bond with one of the carbonyl O moieties of the squaramide unit from the neighboring receptor. This behavior is somewhat similar to the undiluted solution of the KBr and the receptor mixture observed during the ^1^H NMR measurements. The crystal can be considered the most undiluted system. In the solid state, however, intermolecular instead of intramolecular N-H…O hydrogen bonds are present, which is of course associated with the minimization of the energy of all of the molecules frozen in the 3-D crystal lattice. Such intermolecular hydrogen bonds link the moieties in the 1-D supramolecular, centrosymmetric polymers extended along the [010] direction. In such polymers, weak parallel π-π stacking interactions can also be observed between the aromatic rings of the benzo-crown units or between the nitrobenzene and squaramide moieties. The supramolecular polymeric structure formed by **2**, KBr/NaBr salt, and solvent molecules is presented in Figure 4.

**Figure 5:**
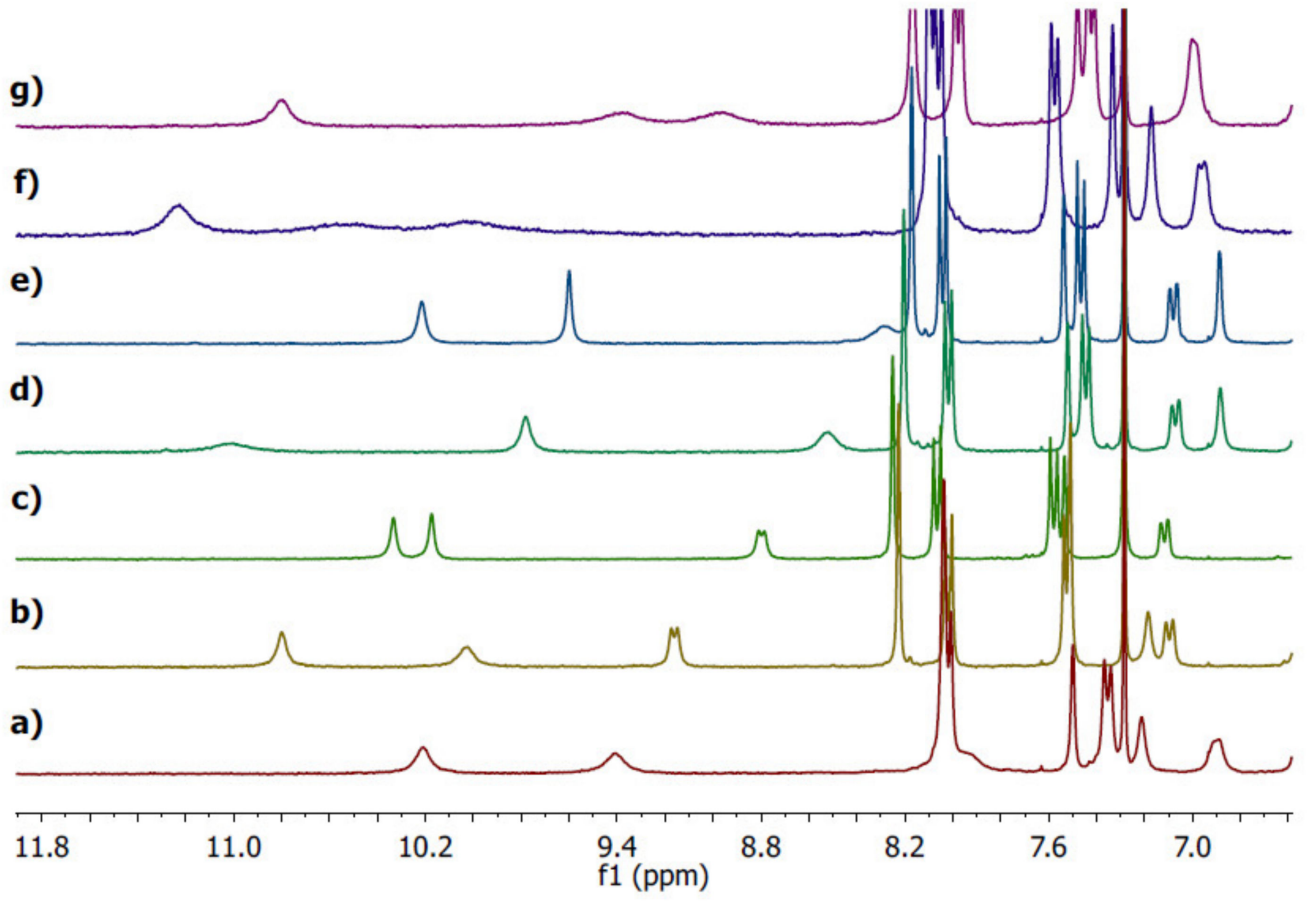
Partial ^1^H NMR spectra of a 3mM solution receptor **2** in CDCl_3_ after contact with: (**a**) water and aqueous 0.5 M solutions of (**b**) KCl, (**c**) KBr, (**d**) KNO_2_, (**e**) KNO_3_, (**f**) K_2_SO_4_, and (**g**) KH_2_PO_4_.

Taking into consideration the anion binding cooperativity of all three NHs together with the enhancement of anion binding that is assured by the assistance of cations, we tested receptor **2** under interfacial conditions. Specifically, we examined whether receptor **2** could be used to extract inorganic salts from the aqueous to organic phase. Qualitative evidence for this was obtained from extraction experiments monitored by the ^1^H NMR control (Figure 5). We found that after putting a solution of **2** in wet CDCl_3_ in contact with aqueous solutions of various potassium salts, considerable changes in the spectrum pattern of **2** in chloroform were noted. Specifically, the most pronounced downfield shifts were found for all signals corresponding to the squaramide and amide protons. These changes demonstrate that all NHs can participate together in anion binding, this time under interfacial conditions. The simultaneous complexation of potassium cations upon extraction by **2** was manifested by the perturbation of the signals corresponding to the crown ether protons and the neighboring aromatic ones after making contact with the aqueous salt solution. Interestingly, the case of potassium sulfate extraction also suggests the effective transfer of this salt from the aqueous to organic phase, since the changes in the spectrum after extraction were among the largest. Receptor **2** exhibited similar behavior in the extraction of sodium salts, although these changes were less pronounced and suggested lesser effectivity for these cases (Figure S70).

**Figure 6:**
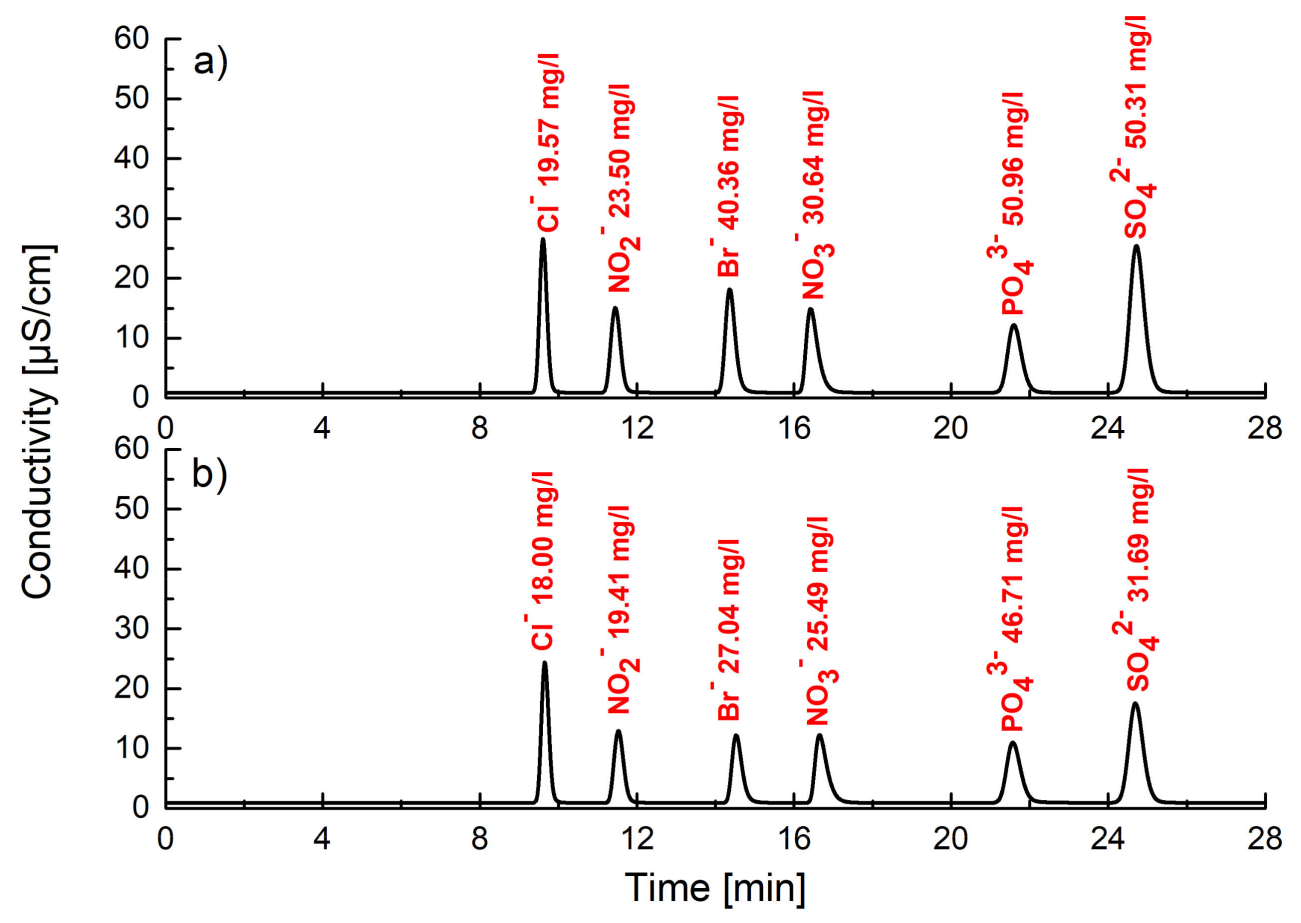
Chromatograms obtained during extraction experiments after tenfold dilution: (**a**) source phase: 5 mM aqueous solution of KCl, KBr, KNO_2_, KNO_3_, K_2_SO_4_, and KH_2_PO_4_ (**b**) after extraction with 20 mM **2** in CHCl_3_.

This is in agreement with the stability constants determined from titration experiments, which showed the formation of weaker complexes of **2** with sodium rather than potassium salts. In order to quantify the extraction experiments and to estimate the extraction efficiency, the ion chromatography technique was applied. We tracked the loss of anions in the aqueous phase after contact with chloroformic solution of **2**. We conducted a series of extractions of 5 mM aqueous solutions of potassium salts with a 5 mM solution of receptor **2** in chloroform and calculated the loss of particular salts to be 4.6, 11.7, 12.4, 12.7, 13.1, and 17.7% for KH_2_PO_4_, KNO_3_, KCl, KNO_2_, K_2_SO_4_, and KBr, respectively. As expected, increasing the concentration of receptor **2** to 20 mM allowed for the more effective removal of potassium salts from an aqueous 5 mM solution of this salt. For instance, the drop in the sulfate concentration in the aqueous phase increased to 37%. Competitive extraction experiments with mixtures of all of the potassium salts tested in the aqueous phase (5 mM each) and using 20 mM of **2** in chloroform shows selectivity towards sulfate and bromide salts (Figure 6). Specifically, the decrease in the salt concentration in the aqueous phase was found to be 8.3, 8.0, 16.8, 17.4, 33, and 37% for KCl, KH_2_PO_4_, KNO_3_, KNO_2_, KBr, and K_2_SO_4_, respectively. Control extraction experiments utilizing receptor **1** and **2** and sodium salts revealed their lower effectivity under such conditions (Figures S71 and S72). On the other hand, extraction experiments with **3** failed since this receptor and its complexes with salts were found to be insoluble in chloroform and caused no homogenous phase separation. This shows that the presence of the crown ether unit in receptor structures of ion pairs not only allows for anion binding enhancement but also assures the solubility of the receptor and its complexes and provides the possibility of acting as a transmembrane transporter. 

**Figure 7:**
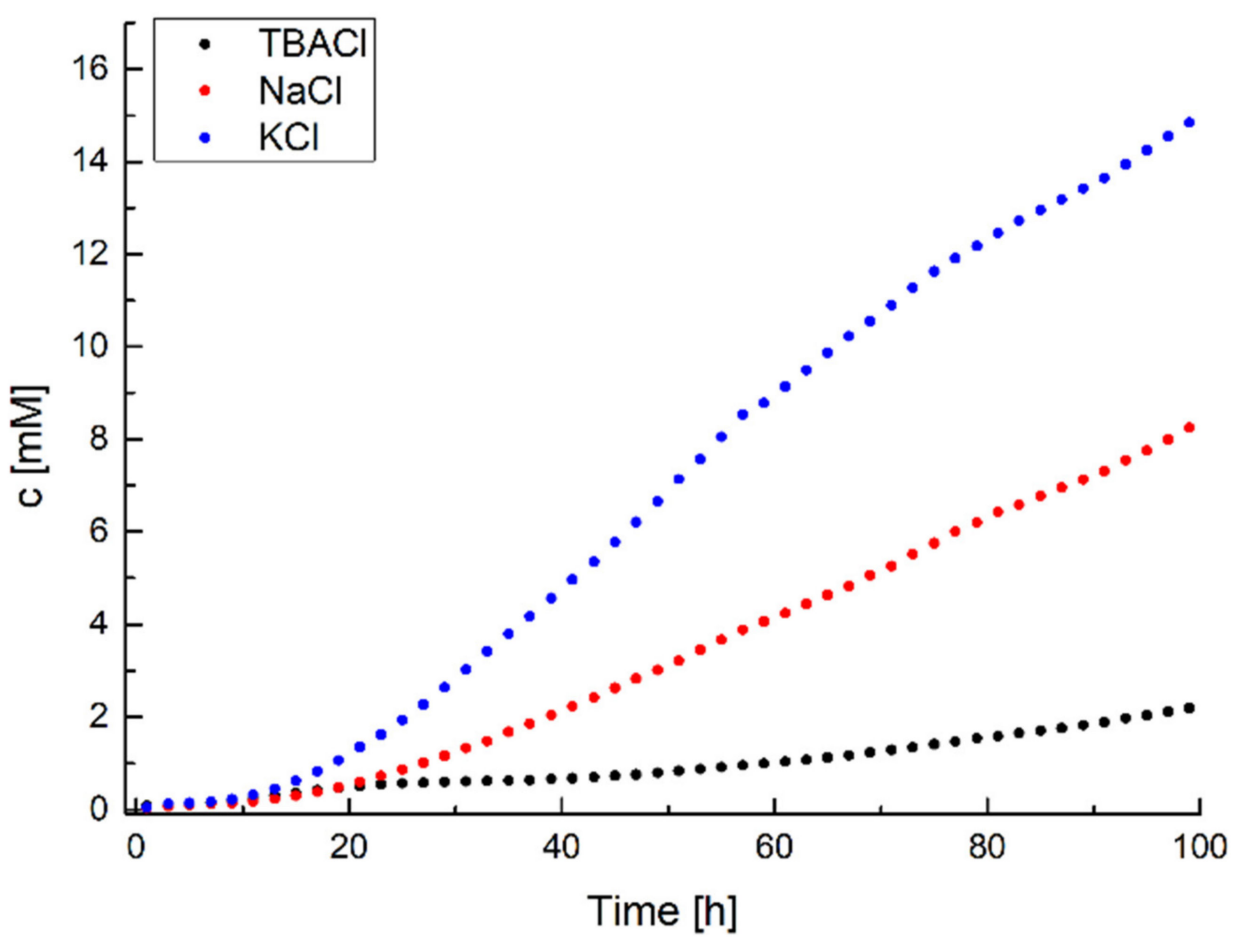
Chloride transport by **2** across chloroform membrane determined by the chloride concentration in the receiving phase. Source phase: 50 mM solution of TBACl, NaCl, or KCl in water; organic phase: 5 mM **2** in CHCl_3_; receiving phase: water.

In this context, U-tube experiments were conducted using a 5 mL 5 mM solution of receptor **2** in chloroform as a bulky membrane and a 5 mL source phase consisting of a 50 mM aqueous solution of TBACl, NaCl, or KCl. The salt content in the receiving phase (5 mL) was monitored using conductometry. We found that receptor **2** is capable of transporting chloride anions across the membrane and that this process depends on the presence of cations. When aqueous TBACl was used as a source phase after 100 h, the chloride anions were transported with an efficiency of 9% (efficiency was defined as the quotient of the concentration of chloride salt in the receiving phase to half of the initial concentration of the salt in the source phase). In the presence of sodium or potassium cations (aq. NaCl or KCl were used as a source phase), a considerable enhancement in the transporting of chloride salts was noted, indicating M^+^/Cl^−^ symport. Specifically, after 100 h, the sodium and potassium chlorides were transported more effectively, with 33% and 59% efficiency, respectively (Figure 7). Importantly, receptor **2** was also found to transport extremely hydrophilic sulfate anions and was able to do so in an enhanced manner with potassium cations, achieving an efficiency of 34% after 100 h (Figure S80). This clearly shows the potency of **2** to act as an effective transporter that is capable of cooperatively transporting ion pairs.

## 3. Materials and Methods

### 3.1. General Consideration

Unless specifically indicated, all other chemicals and reagents used in this study were purchased from commercial sources and were used as received. If necessary, product purification was performed using column chromatography on silica gel (Merck Kieselgel 60, 230–400 mesh) with mixtures of chloroform/methanol. Thin-layer chromatography (TLC) was performed on silica gel plates (Merck Kieselgel 60 F254). The 1H and 13C NMR spectra used for product characterization were recorded on a Bruker 300 spectrometer (Bruker Corporation, Billerica, MA, USA) using a residual protonated solvent as the internal standard. DOSY, ROESY, and HSQC experiments were conducted at 298 K on Varian VNMRS 600 MHz instrument (Varian Inc., Palo Alto, CA, USA) with a residual solvent signal as an internal standard. High resolution mass spectra (HRMS) were measured on a Quattro LC Micromass unit (Waters Corporation, Milford, CT, USA) using the ESI technique. UV-vis analyses were performed using a Thermo Spectronic Unicam UV500 Spectrophotometer (Thermo Fisher Scientific, Waltham, MA, USA). High-performance ion chromatography (HPIC) analyses were performed using a 930 Compact IC Flex apparatus (Metrohm AG, Herisau, Switzerland).

### 3.2. Synthetic Details

**Compound S1:** To a degassed solution of 4-nitrobenzo-15-crown-5 ether (1.10 g, 3.51 mmol) in 45 mL of a THF/MeOH mixture (1:4), 20 mg of 10% Pd/C was added. The reaction mixture was kept under a H_2_ atmosphere (balloon pressure) at room temperature overnight. The catalyst was removed by filtration through a pad of Celite and was washed with MeOH. The filtrate was concentrated under reduced pressure to ensure that the crude product was in a near quantitative yield (0.98 g). The obtained 4-aminobenzo-15-crown-5 ether was used in the next step without further purification.

To the solution of *N*-(*tert-*butoxycarbonyl)-4-nitro-L-phenylalanine (400 mg, 1.29 mmol) and 1,3-dicyclohexylocarbodiimide (293 mg 1.42 mmol) in 10 mL of dry dichloromethane, 4-aminobenzo-15-crown-5 ether (365 mg, 1.29 mmol) at 0 °C (ice bath) was added. The reaction mixture was stirred for 30 min and was then left at room temperature overnight. The precipitate was filtered off, washed with dichloromethane, and the filtrate was evaporated. The residue was purified by silica gel column chromatography (5% methanol in chloroform) to create a title product that was a yellow oil (504 mg, 0.88 mmol, 68% yield).

HRMS (ESI): calcd 598.2377 for C_28_H_37_N_3_O_10_Na [M + Na]^+^: found: 598.2361.

^1^H NMR (300 MHz, CDCl_3_) δ 8.23 (s, 1H, NH_sq_), 8.20–8.10 (m, 2H, CH_Ar_), 7.47–7.40 (m, 2H, CH_Ar_), 7.18 (s, 1H, CH_Ar_), 6.86–6.74 (m, 2H, CH_Ar_), 5.37–5.25 (m, 1H, NH_Am_), 4.65–4.48 (m, 1H, CH_α_), 4.14–4.05 (m, 4H, CH_2 Crown_), 3.95–3.85 (m, 4H, CH_2 Crown_), 3.80–3.72 (m, 8H, CH_2 Crown_), 3.41–3.08 (m, 2H, CH_2_), 1.41 (s, 9H, CH_3_).

^13^C NMR (75 MHz, CDCl_3_) δ 169.1, 155.9, 149.3, 147.0, 146.1, 144.7, 131.3, 130.3, 123.7, 114.6, 112.6, 107.0, 80.8, 70.5, 70.4, 69.6, 69.5, 56.0, 40.9, 38.3, 28.2.

**Compound S4:** To a solution of 3,4-dimethoxy-3-cyclobutane-1,2-dione (2.00 g, 14.1 mmol) in methanol (20 mL), 3,5-bis(trifluoromethyl)aniline (2.40 mL, 14.5 mmol, 1.1 equiv) was added at room temperature. After being stirred for 3 days, the reaction mixture was filtered, and the collected solid material was washed with MeOH. The obtained light yellow solid was dried in vacuo to produce the desired product (4.54 g, 13.4 mmol, 95%).

HRMS (ESI): calcd for C_13_H_7_F_6_NO_3_Na [M + Na]^+^: 362.0228, found: 362.0225.

^1^H NMR (300 MHz, DMSO-d_6_) δ 11.19 (s, 1H, NH_sq_), 8.05 (s, 2H, CH_Ar_), 7.79 (s, 1H, CH_Ar_), 4.42 (s, 3H, CH_3_).

^13^C NMR (75 MHz, DMSO-d_6_) δ 187.4, 184.5, 179.9, 169.1, 140.2, 131.9, 131.4, 131.0, 130.5, 128.5, 124.9, 121.3, 119.2, 117.6, 116.1, 60.9.

**Receptor 1:** Compound S1 (500 mg, 0.87 mmol) was dissolved in 20 mL dichloromethane, and 5 mL of trifluoroacetic acid was added. The reaction mixture was stirred at room temperature until the starting material was consumed (TLC controlled). The reaction mixture was diluted in dichloromethane and was then treated with saturated and solid NaHCO_3_ to neutralize the trifluoroacetic acid. The biphasic mixture was separated, and the organic phase was dried by evaporating the solvent several times. The obtained amine was used in the next step without further purification. 

To a solution of the abovementioned amine (429 mg, 0.9 mmol) in 10 mL of methanol, triethylamine (138 μL, 1.0 mmol) and squaramide **S4** (306 mg, 0.9 mmol) were added. After being stirred overnight in room temperature, the reaction mixture was concentrated, and the residue was purified by silica gel column chromatography (2% methanol in chloroform) to ensure that receptor **1** was a yellow solid (493 mg, 70% yield). 

HRMS (ESI): calcd 805.1920 for C_35_H_32_F_6_N_4_O_10_Na [M + Na]^+^: found: 805.1930. M.p. 152–154 °C.

IR: 3275 cm^−1^ (stretching N-H), 1795 cm^−1^ (stretching C=O), 1686 cm^−1^ (stretching C=O).

^1^H NMR (300 MHz, DMSO-d_6_) δ 10.57 (s, 1H, NH_sq_), 10.44 (s, 1H, NH_Am_), 8.45–8.30 (m, 1H, NH_Sq_), 8.23–8.12 (m, 2H, CH_Ar_), 8.06 (s, 2H, CH_Ar_), 7.65 (s, 1H, CH_Ar_), 7.57–7.45 (m, 2H, CH_Ar_), 7.25–7.17 (m, 1H, CH_Ar_), 7.16–7.05 (m, 1H, CH_Ar_), 6.99–6.88 (m, 1H, CH_Ar_), 5.25–5.13 (m, 1H, CH_α_), 4.13–3.92 (m, 4H, CH_2 Crown_), 3.86–3.71 (m, 4H, CH_2 Crown_), 3.67–3.55 (m, 8H, CH_2 Crown_), 3.40–3.19 (m, 2H, CH_2_). 

^13^C NMR (75 MHz, DMSO-d_6_) δ 184.4, 181.1, 169.1, 167.9, 163.4, 148.9, 147.0, 145.6, 144.7, 141.6, 132.3, 132.1, 131.6, 131.5, 125.4, 123.7, 121.8, 118.4, 114.8, 112.7, 106.9, 70.8, 70.2, 70.1, 69.3, 69.2, 68.7, 58.2, 41.1. 

**Compound S2**: To a degassed solution of 4-nitrobenzo-18-crown-6 ether (797 mg, 2.23 mmol) in 45 mL of a THF/MeOH mixture (1:4), 20 mg of 10% Pd/C was added. The reaction mixture was kept under a H2 atmosphere (balloon pressure) at room temperature overnight. The catalyst was removed by filtration through a Celite pad and was washed with MeOH. The filtrate was concentrated under reduced pressure to ensure that the crude product was in near quantitative yield (730 mg). The obtained 4-aminobenzo-18-crown-6 ether was used in the next step without further purification.

To a solution of N-(tert-butoxycarbonyl)-4-nitro-L-phenylalanine (691 mg, 2.23 mmol) and 1,3-dicyclohexylocarbodiimide (506 mg 2.45 mmol) in 20 mL of dry dichloromethane, 4-aminobenzo-18-crown-6 ether (730 mg, 2.23 mmol) at 0 °C (ice bath) was added. The reaction mixture was stirred for 30 min and was then left at room temperature overnight. The precipitate was filtered off, washed with dichloromethane, and the filtrate was evaporated. The residue was purified by silica gel column chromatography (5% methanol in chloroform) to create a title product that was a yellow oil (1.24 g, 2.11 mmol, 90% yield).

HRMS (ESI): calcd 642.2640 for C_30_H_40_N_3_O_11_Na [M + Na]^+^: found: 642.2611.

^1^H NMR (300 MHz, CDCl_3_) δ 8.37 (s, 1H, NH_Am_), 8.17–8.10 (m, 2H, CH_Ar_), 7.48–7.40 (m, 2H, CH_Ar_), 7.18 (s, 1H, CH_Ar_), 6.88–6.73 (m, 2H, CH_Ar_), 5.44–5.34 (m, 1H, NH_am_), 4.66–4.49 (m, 1H, CH_α_), 4.18–4.04 (m, 4H, CH_2 Crown_), 3.95–3.84 (m, 4H, CH_2 Crown_), 3.81–3.64 (m, 12H, CH_2 Crown_), 3.36–2.72 (m, 2H, CH_2_), 1.40 (s, 9H, CH_3_). 

^13^C NMR (75 MHz, CDCl_3_) δ 168.9, 155.8, 149.0, 147.0, 145.9, 144.7, 131.3, 130.3, 123.7, 114.5, 113.5, 112.7, 107.1, 80.9, 70.8, 70.7, 69.7, 69.5, 56.1, 38.1, 28.2.

**Receptor 2:** Compound **S2** (966 mg, 1.56 mmol) was dissolved in 30 mL dichloromethane, and 10 mL of trifluoroacetic acid was added. The reaction mixture was stirred at room temperature until the starting material was consumed (TLC controlled). The reaction mixture was diluted with dichloromethane then was treated with saturated and solid NaHCO_3_ to neutralize the trifluoroacetic acid. The biphasic mixture was separated, and the organic phase was dried by evaporating the solvent several times. The obtained amine was used in the next step without further purification. 

To a solution of the abovementioned amine (810 mg, 1.56 mmol) in 20 mL of methanol, triethylamine (240 μL, 1.72 mmol) and squaramide **S4** (529 mg, 1.56 mmol) were added. After being stirred overnight at room temperature, the reaction mixture was concentrated, and the residue was purified by silica gel column chromatography (5% methanol in chloroform) to ensure that receptor **2** was a yellow solid (841 mg, 65% yield). 

HRMS (ESI): calcd 849.2183 for C_37_H_36_F_6_N_4_O_11_Na [M + Na]^+^: found: 849.2186. M.p. 132–133 °C.

IR: 3275 cm^−1^ (stretching N-H), 1795 cm^−1^ (stretching C=O), 1686 cm^−1^ (stretching C=O).

^1^H NMR (300 MHz, DMSO-d6) δ 10.49 (s, 1H, NH_Sq_), 10.44 (s, 1H, NH_Am_), 8.40–8.28 (m, 1H, NH_Sq_), 8.20–8.12 (m, 2H, CH_Ar_), 8.06 (s, 2H, CH_Ar_), 7.65 (s, 1H, CH_Ar_) 7.54–7.46 (m, 2H, CH_Ar_), 7.24–7.17 (m, 1H, CH_Ar_), 7.15–7.05 (m, 1H, CH_Ar_), 6.97–6.89 (m, 1H, CH_Ar_), 5.27–5.14 (m, 1H, CH_α_), 4.10–3.98 (m, 4H, CH_2 Crown_), 3.83–3.70 (m, 4H, CH_2 Crown_), 3.66–3.49 (m, 12H, CH_2 Crown_), 3.44–3.19 (m, 2H, CH_2_). 

^13^C NMR (75 MHz, DMSO-d6) δ 184.4, 181.1, 169.0, 167.8, 163.3, 148.5, 147.0, 145.3, 144.7, 141.5, 132.1, 131.6, 131.5, 125.4, 123.7, 113.9, 112.5, 106.4, 70.3, 69.2, 69.1, 68.8, 68.5, 58.1, 41.2.

**Compound S3:** To a solution of N-(tert-butoxycarbonyl)-4-nitro-L-phenylalanine (500 mg, 1.61 mmol) and 1,3-dicyclohexylocarbodiimide (366 mg 1.77 mmol) in 20 mL of dry dichloromethane, aniline (150 mg, 1.61 mmol) at 0 °C (ice bath) was added. The reaction mixture was stirred for 30 min and was then left at room temperature overnight. The precipitate was filtered off, washed with dichloromethane, and the filtrate was evaporated. The residue was purified by silica gel column chromatography (5% methanol in chloroform) to produce a title product that was a yellow oil (565 mg, 1.47 mmol, 91% yield).

HRMS (ESI): calcd 408.1535 for C_20_H_23_N_3_O_5_Na [M + Na]^+^, found: 408.1552. 

^1^H NMR (300 MHz, DMSO-d_6_) δ 10.12 (s, 1H, NH_Am_), 8.22–8.16 (m, 2H, CH_Ar_), 7.67–7.57 (m, 4H, CH_Ar_), 7.36–7.28 (m, 2H, CH_Ar_), 7.27–7.20 (m, 1H, NH_Am_), 7.10–7.03 (m, 1H, CH_Ar_), 4.47–4.34 (m, 1H, CH_α_), 3.21–2.94 (m, 2H, CH_2_), 1.30 (s, 9H, CH_3_). 

^13^C NMR (75 MHz, DMSO-d_6_) δ 170.1, 155.9, 147.0, 146.7, 139.3, 131.1, 129.2, 123.9, 123.6, 119.8, 78.7, 56.5, 37.7, 28.5.

**Receptor 3:** Compound **S3** (565 mg, 1.47 mmol) was dissolved in 20 mL dichloromethane, and 7 mL of trifluoroacetic acid was added. The reaction mixture was stirred at room temperature until the starting material was consumed (TLC controlled). The reaction mixture was diluted in dichloromethane then was treated with saturated and solid NaHCO_3_ to neutralize the trifluoroacetic acid. The biphasic mixture was separated, and the organic phase was dried by evaporating the solvent several times. The obtained amine was used in the next step without further purification. 

To a solution of the abovementioned amine (354 mg, 1.24 mmol) in 20 mL of methanol, squaramide **S4** (421 mg, 1.24 mmol) were added. After being stirred overnight in room temperature, the reaction mixture was filtrated, and the collected solid material was washed several times with MeOH. The obtained light yellow solid was dried in vacuo to produce the desired receptor **3** (418 g, 0.71 mmol, 57%).

IR: 3275 cm^−1^ (stretching N-H), 1800 cm^−1^ (stretching C=O), 1686 cm^−1^ (stretching C=O). M.p. 290–292 °C.

HRMS (ESI): calcd 615.1079 for C_27_H_18_F_6_N_4_O_5_Na [M + Na]^+^:, found: 615.1089.

^1^H NMR (300 MHz, DMSO-*d*_6_) δ 10.59 (s, 1H, NH_Sq_), 10.47 (s, 1H, NH_Am_), 8.35–8.26 (s, 1H, NH_Sq_), 8.22–8.12 (m, 2H, CH_Ar_), 8.06 (s, 2H, CH_Ar_), 7.69 (s, 1H, CH_Ar_), 7.64–7.55 (m, 2H, CH_Ar_), 7.54–7.46 (m, 2H, CH_Ar_), 7.42–7.31 (m, 2H, CH_Ar_), 7.17–7.07 (m, 1H, CH_Ar_), 5.33–5.20 (m, 1H, CH_α_), 3.43–3.19 (m, 2H, CH_2_). 

^13^C NMR (75 MHz, DMSO-*d*_6_) δ 184.5, 181.1, 169.0, 168.3, 163.4, 147.0, 144.6, 141.5, 138.6, 132.1, 131.5, 129.4, 125.4, 124.5, 123.7, 121.8, 120.1, 118.4, 115.3, 58.1, 41.2. 

**Receptor 4:** To a degassed solution of cinnamic acid (1.23 g, 8.30 mmol) in 45 mL of a THF/MeOH mixture (1:4), 20 mg of 10% Pd/C was added. The reaction mixture was kept under a H_2_ atmosphere (balloon pressure) at room temperature overnight. The catalyst was removed by filtration through a Celite pad and was washed with MeOH. The filtrate was concentrated under reduced pressure to produce a crude product with a near quantitative yield (1.21 g). In the next step, 3-phenylpropionic acid (1.21 g, 8.06 mmol) was dissolved in 30 mL of chloroform and was cooled to 0⁰ in an ice bath. Concentrated nitric acid (5 mL) was added dropwise over 15 min to keep the temperature at 0⁰, and 2.5 mL of concentrated sulfuric acid was added dropwise over 15 min. Then, after 1h of stirring, the reaction mixture was diluted with chloroform (15 mL) and was poured into water (50 mL). The organic phase was separated and was washed with water (2 × 20 mL). Aqueous phases were combined and washed with chloroform (20 mL). Organic phases were dried with Na_2_SO_4_, and solvent was evaporated to produce a mixture of *ortho* and *para* isomers of3-(nitro phenyl)propionic acid (1.14 g). This mixture was purified by means of crystallization from a hexane/ethyl acetate mixture, which produced 3-(4-nitrophenyl)propionic acid (500 mg, 32% yield). 

To the solution of 3-(4-nitrophenyl)propionic acid (227 mg, 1.16 mmol) and 1,3-dicyclohexylocarbodiimide (264 mg 1.28 mmol) in 10 mL of dry dichloromethane, 4-aminobenzo-18-crown-6 ether (379 mg, 1.16 mmol) at 0 °C (ice bath) was added. The reaction mixture was stirred for 30 min and was then left at room temperature overnight. The precipitate was filtered off, washed with dichloromethane, and the filtrate was evaporated. The residue was purified by silica gel column chromatography (5% methanol in chloroform) to create a title product that was a solid yellow (310 mg, 0.61 mmol, 53% yield).

HRMS (ESI): calcd 527.2006 for C_25_H_32_N_2_O_9_Na [M + Na]^+^, found: 527.1992. M.p. 141–143 °C.

IR: 3300 cm^−1^ (stretching N-H), 1660 cm^−1^ (stretching C=O).

^1^H NMR (300 MHz, DMSO-*d*_6_) δ 9.80 (s, 1H, NH_Am_), 8.21–8.12 (m, 2H, CH_Ar_), 7.59–7.50 (m, 2H, CH_Ar_), 7.26 (s, 1H, CH_Ar_), 7.09–7.01 (m, 1H, CH_Ar_), 6.91–6.82 (m, 1H, CH_Ar_), 4.08–3.94 (m, 4H, CH_2 Crown_), 3.82–3.68 (m, 4H, CH_2 Crown_), 3.65–3.47 (m, 12H, CH_2 Crown_), 3.10–2.99 (m, 2H, CH_2_), 2.72–2.59 (m, 2H, CH_2_). 

^13^C NMR (75 MHz, DMSO-*d*_6_) δ 169.8, 150.2, 148.5, 146.4, 144.6, 133.4, 130.1, 123.9, 114.1, 111.8, 106.1, 70.3, 69.3, 69.2, 68.9, 68.6, 37.4, 31.0.

### 3.3. X-ray Measurements

The X-ray measurements of **2** + KBr/NaBr were performed at 130.0(5) K on a Bruker D8 Venture Photon II diffractometer equipped with a INCOATEC IμS micro-focus source (CuKα, *λ* = 1.54178 Å) and a mirror monochromator. A total of 5578 frames were collected with the Bruker APEX3 program [61]. The frames were integrated with the Bruker SAINT software package [62] using a narrow-frame algorithm. The integration of the data using a triclinic unit cell yielded a total of 34,181 reflections to a maximum *θ* angle of 66.48° (0.84 Å resolution), of which 7656 were independent (average redundancy 4.465, completeness = 99.7%, *R_int_* = 4.36%, *R_sig_* = 3.06%) and 6766 (88.38%) were greater than **2***σ*(*F*^2^). The final cell constants of *a* = 9.1517(3) Å, *b* = 14.9141(4) Å, *c* = 17.4557(5) Å, *α* = 66.9565(14)°, *β* = 83.4708(16)°, *γ* = 87.1685(15)°, and *V* = 2178.16(11) Å^3^ are based upon the refinement of the XYZ-centroids of 9989 reflections above 20 *σ*(*I*) with 5.531° < 2*θ* < 133.1°. Data were corrected for absorption effects using the multi-scan method (SADABS) [63]. The ratio of minimum to maximum apparent transmission was 0.844. The calculated minimum and maximum transmission coefficients (based on crystal size) were 0.640 and 0.949.

The structure was solved and refined using the SHELXTL Software Package [64,65] using the space group *P*$\bar{1}$, with *Z* = 1 for the formula unit, C_78_H_81.47_Br_2_F_12_K_0.27_N_10_Na_1.73_O_23.73_. The final anisotropic full-matrix least-squares refinement on *F*^2^ with 782 variables converged at *R*1 = 5.17% for the observed data and at *wR*2 = 14.65% for all data. The goodness-of-fit was 1.051. The largest peak in the final difference electron density synthesis was 0.763 e^−^/Å^3^, and the largest hole was −0.675 e^−^/Å^3^ with an RMS deviation of 0.065 e^−^/Å^3^. On the basis of the final model, the calculated density was 1.507 g/cm^3^ and *F*(000), 1011 e^−^. CCDC 2,079,937 contains the supplementary crystallographic data for this paper. The data can be obtained free of charge from The Cambridge Crystallographic Data Centre via www.ccdc.cam.ac.uk/structures (accessed on 28 August 2021). All of the details concerning the crystal data and structural parameters of 2 + KBr/NaBr are collected in Table 3.

**Figure 8:**
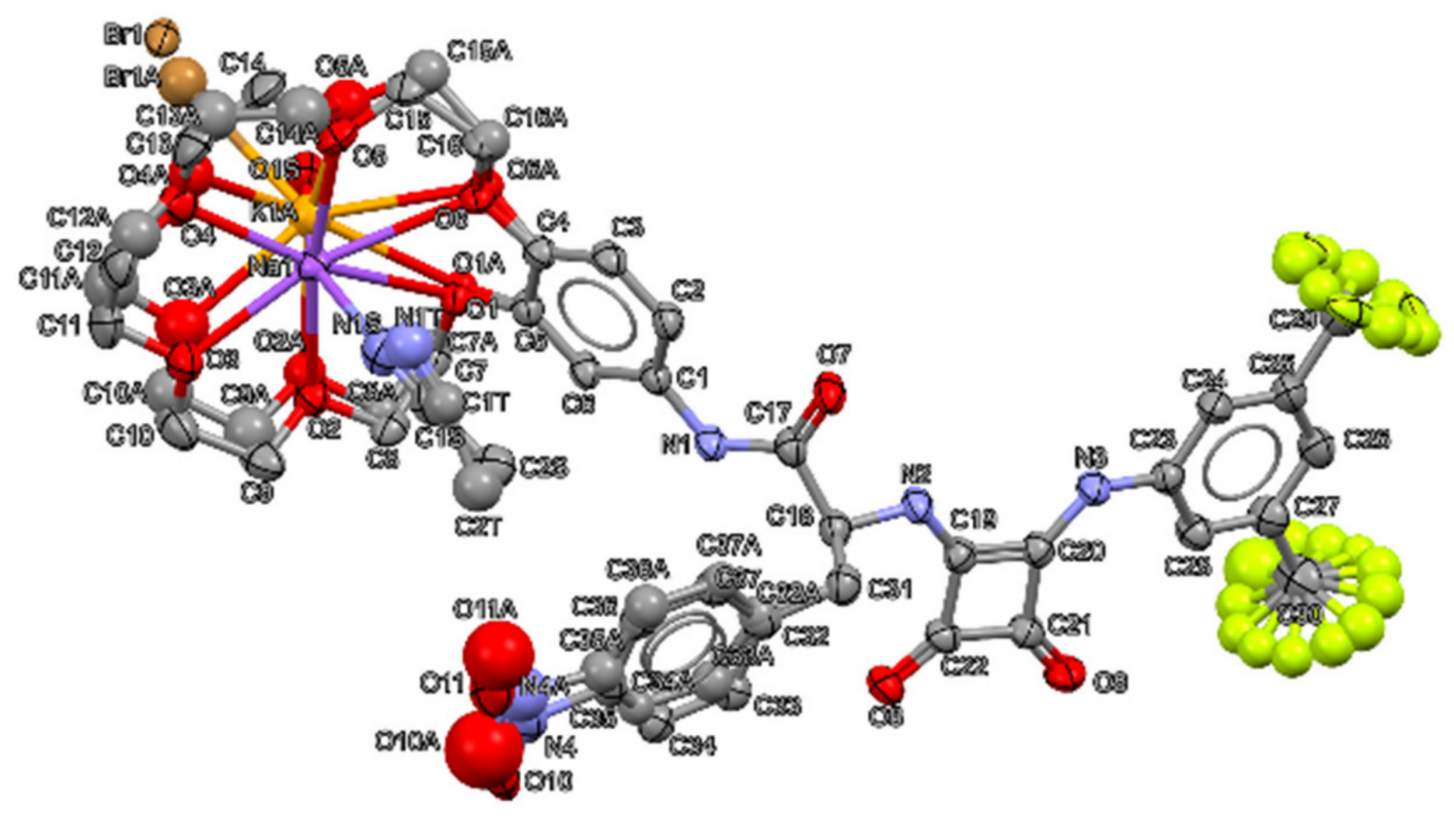
Thermal ellipsoid plot of a 50% probability level together with the numbering scheme of atoms in the structure of **2** + KBr/NaBr. Hydrogen atoms are omitted for clarity.

The structure contains a non-stoichiometric amount of NaBr/KBr salt. The cation is present in the crown ether ring. However, due to different sizes of the Na^+^ and K^+^ ions, the positions of these species are different; nonetheless, the crown ether moiety bears 100% of the Na^+^/K^+^ moiety with a refined ratio yielding 0.866(2)/0.134(2). This imposes a disorder of the aliphatic part of the crown ether ring, a disorder of the nitrobenzene moiety, and a disorder of the ligands complexing/located close to cationic site–indeed, the bromide anion and CH_3_CN molecule together with the nitrobenzene part are disordered over two positions, with the same refined ratio as applied for the cations. The structure also contains a non-stoichiometric amount of water molecules, which link the Na^+^ cation with the Br^−^ anion; thus, the occupancy of the H_2_O in the structure yields 0.866(2). In addition, both CF_3_ groups in the ligand are disordered over four positions each, presenting as a rotation along the C-CF_3_ bonds. The refined occupancy ratios are equal to 0.405(3):0.248(3):0.237(3):0.109(3) and 0.309(3):0.160(3):0.338(3):0.193(3) for the C(29) and C(30) CF_3_ groups, respectively. All of the ordered non-hydrogen atoms and major component-disordered moieties (except F atoms) were refined anisotropically. Most of the hydrogen atoms were placed in calculated positions and were refined within the riding model. The positions of three hydrogen atoms engaged in hydrogen bonds (all N-H groups) were freely refined. The positions of the H atoms of the water molecules were restrained. The temperature factors of the hydrogen atoms were not refined (except for those of the H_2_O molecules) and were set to be either 1.2 or 1.5 times larger than the Ueq of the corresponding heavy atom. The atomic scattering factors were taken from the International Tables [66]. Molecular graphics were prepared using the program Mercury 4.1 [67]. Thermal ellipsoid parameters are presented at a 50% probability level in Figure 8.

## 4. Conclusions

Summing up, we have developed the synthesis of highly effective ion pair receptors that are capable of binding anions in an enhanced manner with the assistance of alkali metal cations. The synthesis relies on the sequential decoration of an amino acid platform with binding sites. The α-amino function of the amino acid was converted into squaramide moiety, enabling interaction with anions. A benzocrown ether unit, designed as a cation binding site, was introduced via amide bond formation. This linkage not only allowed for cation binding but also opened up the possibility of supporting the squaramide function in anion binding, reinforcing ion pair binding overall. Based on UV-vis, ^1^H NMR, 2D NMR, and DOSY spectroscopy in the MeCN and X-ray solid state measurements, the binding properties of the receptors were established. The most effective ion pair receptor **2** was found to be deprotonated with carboxylate anions and formed 1:1 complexes with other monovalent salts. More complex equilibria were recognized upon the interaction of **2** with sulfates. Receptors **1**–**3** were found to form intramolecular hydrogen bonds, applying the amide unit as hydrogen bond donor. In the presence of anions, the amide function switched to interaction with anions, allowing all three NHs to act simultaneously. In contrast to the monotopic receptor **3** or the amide-based ion pair receptor **4**, the squaramide-based ditopic receptor **2** can extract ion pairs from the aqueous phase to the organic phase, with selectivity towards sulfates and bromides. The ability of receptor **2** to take up and to release salts and its possibility of symporting ion pairs was utilized in cation-enhanced transport experiments of chloride and sulfate anions across a bulky chloroform membrane.

## Figures and Tables

**Table 1:** Apparent association constants (*K*_a_) for interactions between receptors **1**–**3** and chloride anions without and in the presence of one equivalent of sodium perchlorate or potassium hexafluorophosphate ^a^.

	TBACl	TBACl + 1 eq. NaClO_4_	TBACl + 1 eq. KPF_6_	K_Na+_/K_TBA+_	K_K+_/K_TBA+_
**1**	2.71 × 10^5^	3.25 × 10^5^	-	1.20	-
**2**	2.75 × 10^5^	3.49 × 10^5^	5.68 × 10^5^	1.27	2.13
**3**	1.98 × 10^5^	1.83 × 10^5^	1.67 × 10^5^	0.92	0.84
**S2**	3.40 × 10^3^	5.70 × 10^3^	6.80 × 10^3^	1.68	2.00

^a^ UV-Vis, solvent CH_3_CN, 293 K, [1] = 2.52 × 10^−5^ M, [2] = 2.50 × 10^−5^ M, [3] = 2.46 × 10^−5^ M, chloride added as TBACl salt, [TBACl] ~0.8 mM; M^−1^, errors < 10%.

**Table 2:** Apparent association constants (*K*_a_) for interactions between receptor **2** and selected anions and their apparent association for the interaction of receptor **2** with anions in the presence of one equivalent of sodium perchlorate or potassium hexafluorophosphate ^a^.

	TBACl	TBACl + 1 eq. NaClO_4_	TBACl + 1 eq. KPF_6_
Cl^−^	2.75 × 10^5^	3.49 × 10^5^	5.68 × 10^5^
Br^−^	2.52 × 10^4^	4.11 × 10^4^	4.40 × 10^4^
NO_2_^−^	1.33 × 10^5^	1.67 × 10^5^	1.75 × 10^5^
NO_3_^−^	5.73 × 10^3^	6.30 × 10^3^	6.50 × 10^3^
SO_4_^2−^	^b^	^b^	^b^
PhCOO^−^	^c^	^c^	^c^
CH_3_COO^−^	^c^	^c^	^c^

^a^ UV-Vis, solvent CH3CN, 293 K, [2] = 2.50 × 10^−5^ M, anions added as TBA salt, [TBAX] ~0.8 mM; M^−1^, errors < 10%. ^b^ We could not fit the obtained data to the appropriate binding model. ^c^ Deprotonation.

**Table 3:** Data collection and structure refinement for **2** + KBr/NaBr.

Formula	C_78_H_81.47_Br_2_F_12_K_0.27_N_10_Na_1.73_O_23.73_, corresponding to: 2 × **2** + 1.73 × NaBr + 0.27 × KBr + 2.00 × acetonitrile + 1.73 × H_2_O
M_x_/g∙mol^−1^	1976.83
T/K	130.0(5)
λ/Å	1.54178
Crystal size	0.023 × 0.122 × 0.213 mm
Space group	P$\bar{1}$
Unit cell dimensions	a = 9.1517(3) Å α = 66.9565(14)° b = 14.9141(4) Å β = 83.4708(16)° c = 17.4557(5) Å γ = 87.1685(15)°
V/Å^3^, Z	2178.16(11), 1
D_x_/g∙cm^−3^	1.507
μ/mm^−1^	2.300
F(000)	1011
θ_min_, θ_max_	2.77°, 66.48°
Index ranges (merged data)	-10 ≤ h ≤ 10, −17 ≤ k ≤ 17, −20 ≤ l ≤ 20
Reflections collected/independent	34181/7656, R_int_ = 0.0436
Completeness	99.7%
Absorption correction	Multi-Scan
T_max_, T_min_	0.949, 0.640
	
Refinement method	Full-matrix LSQ on F^2^
Data/restraints/parameters	7656/181/782
GOF on F^2^	1.051
Final R indices	6766 data; I > 2σ(I) R1 = 0.0517, wR2 = 0.1390 all data R1 = 0.0589, wR2 = 0.1465
Δρ_max_, Δρ_min_	0.763, −0.675 e∙Å^−3^

## Data Availability

Not applicable.

## Supplementary Materials

Supplementary Materials can be found at https://www.mdpi.com/article/10.3390/ijms221910754/s1.
