# Supplementary material for: Utilizing an Amino Acid Scaffold to Construct Heteroditopic Receptors Capable of Interacting with Salts under Interfacial Conditions
Source: Int J Mol Sci. 2021 Oct 5;22(19):10754. doi: 10.3390/ijms221910754 (PMC8509731; doi:10.3390/ijms221910754)

Utilizing an amino acid scaffold to construct heteroditopic receptors capable of interacting with salts under interfacial conditions

by

Damian Jagleniec, Natalia Walczak, Łukasz Dobrzycki, Jan Romański

Faculty of Chemistry, University of Warsaw, Pasteura 1, PL 02-093 Warsaw, Poland

*jarom@chem.uw.edu.pl

TABLE OF CONTENTS

1. UV-vis measurements (JOB Plots, Binding isotherms) 1

2. NMR measurements (Binding isotherms, 2D NMR spectra) 25

3. Extraction experiments 40

4. NMR spectra 46

UV-vis titration experiments

The UV-Vis titration was performed using Thermo Spectronic Unicam UV500 Spectrophotometer at 298K in acetonitrile. In each case, a 2500 μL of freshly prepared 3.05×10^{-5} M solution of receptor was added to a cuvette and small aliquots of TBAX, containing constant concentration of the receptor, were added and a spectrum was acquired after each addition. In the case of ion pair titration receptor was firstly pretreated with one equivalent of KPF_6 or $NaClO_4$ (refers to receptor). The resulting titration data were analyzed using BindFit (v0.5) package, available online at http://supramolecular.org. Each titration was carried out in duplicate. Reported values are calculated as weighted arithmetic mean, where the weights were the errors obtained for each value separately. The given uncertainty of the association constants is the largest of the variance (external or internal)

Fig. S1. Dilution curve of receptor **1** in CH_3CN.

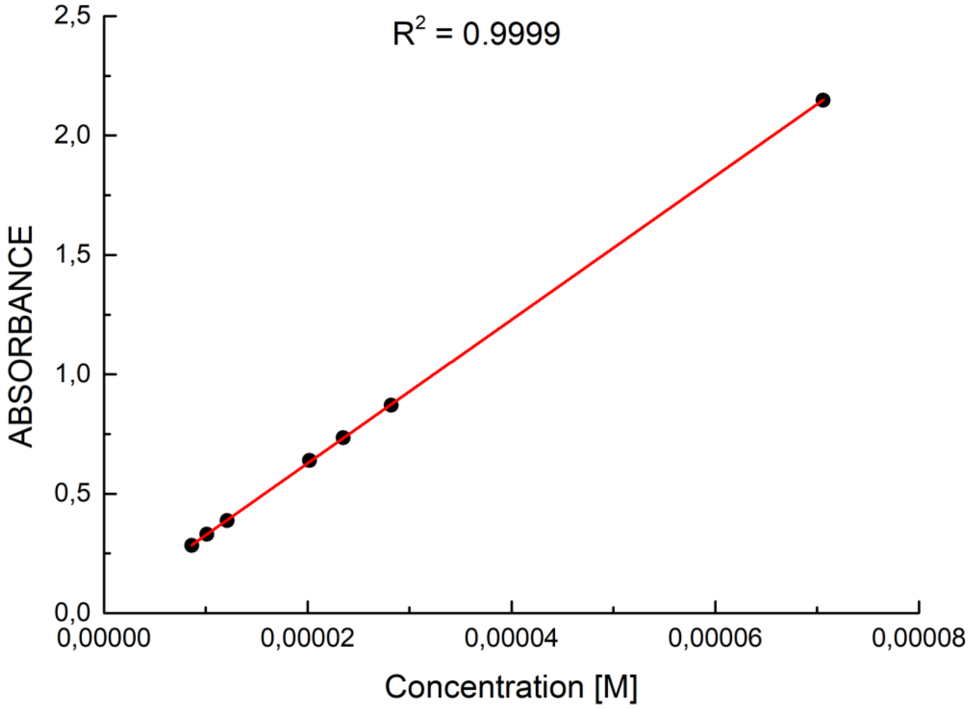

Fig. S2. Dilution curve of receptor **2** in CH_3CN.

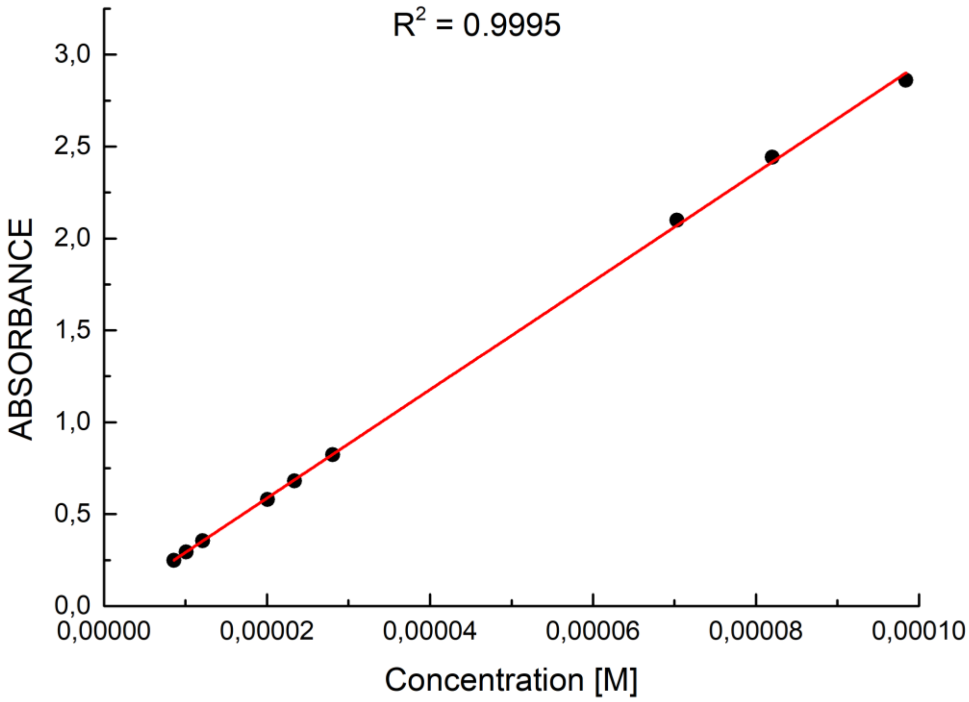

Fig. S3. Dilution curve of receptor **3** in CH_3CN.

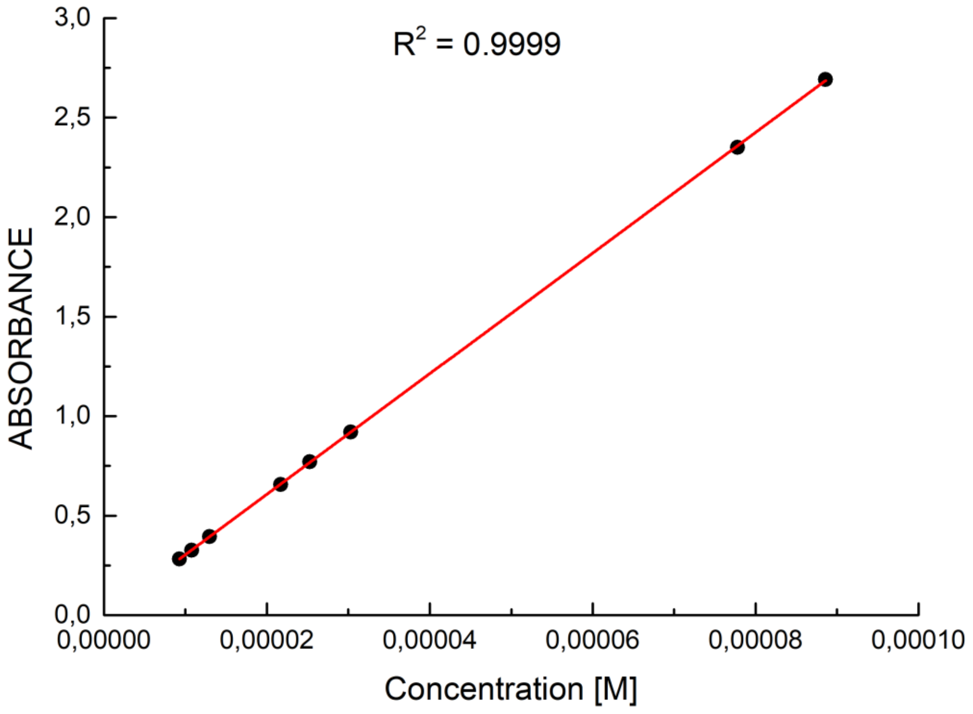

Fig. S4. Job plot analysis for **1** + TBACl in CH_3CN.

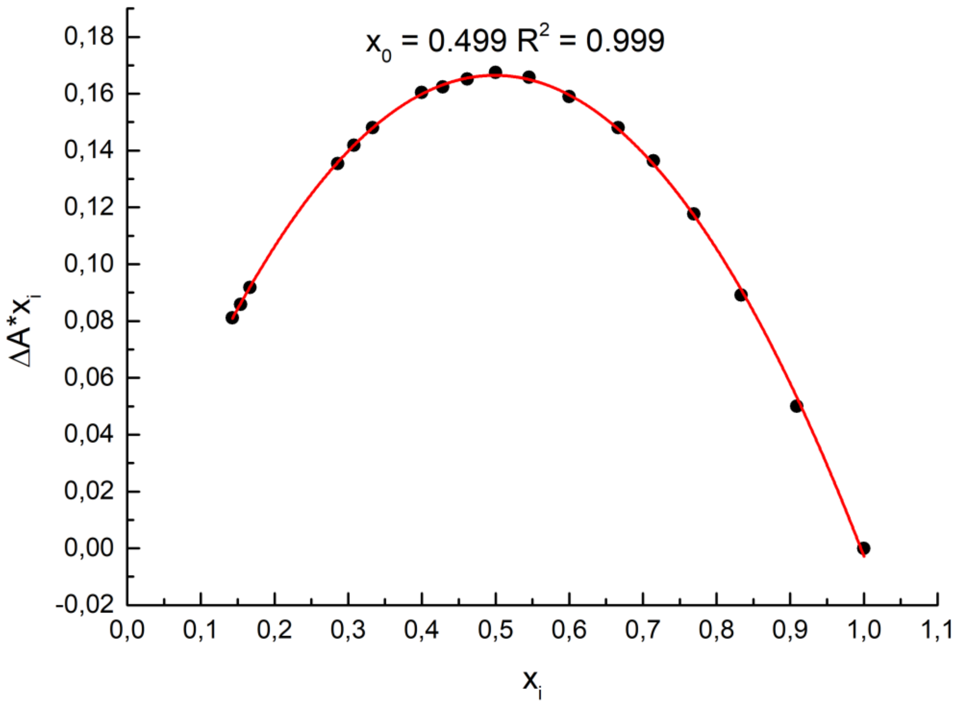

Fig. S5. Job plot analysis for **1** + TBABr in CH_3CN.

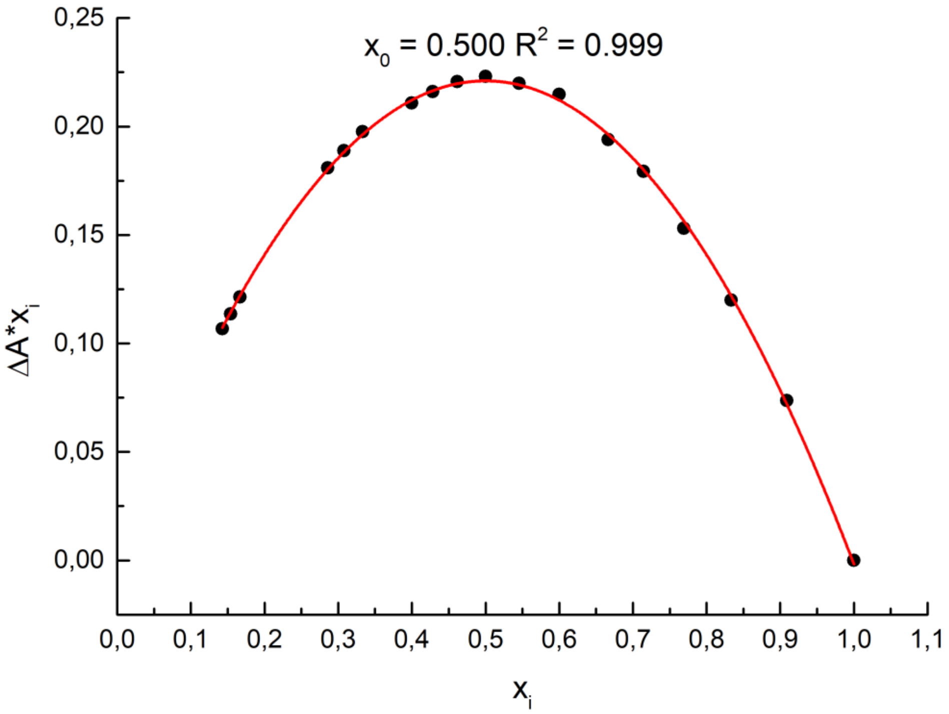

Fig. S6. Job plot analysis for **1** + $NaClO_4$ in CH_3CN.

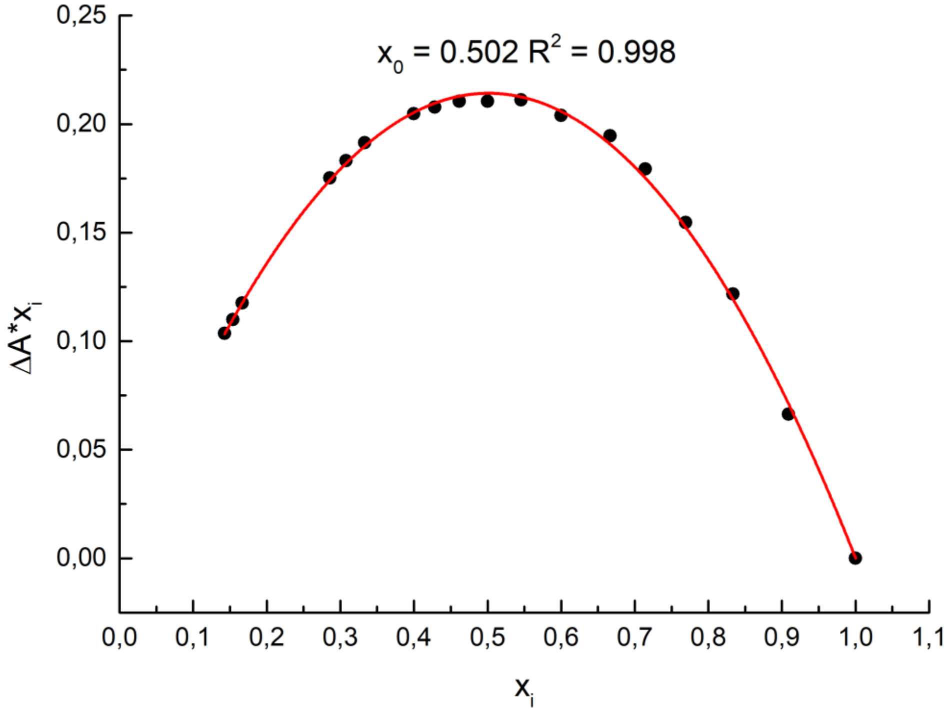

Fig. S7. Job plot analysis for **1** + KPF_6 in CH_3CN.

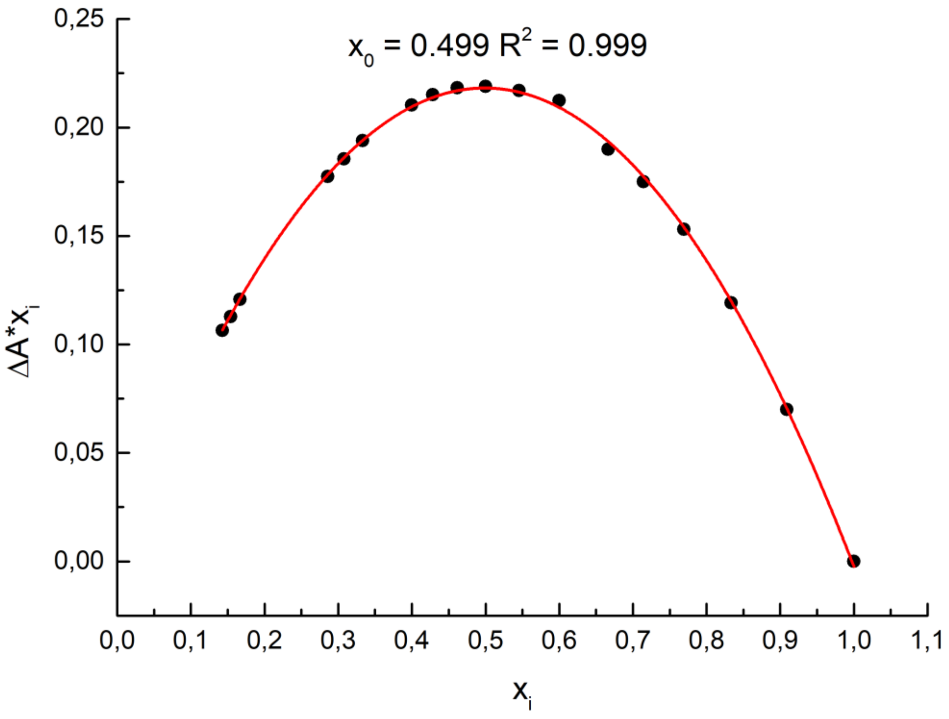

Fig. S8. Job plot analysis for **2** + TBACl in CH_3CN.

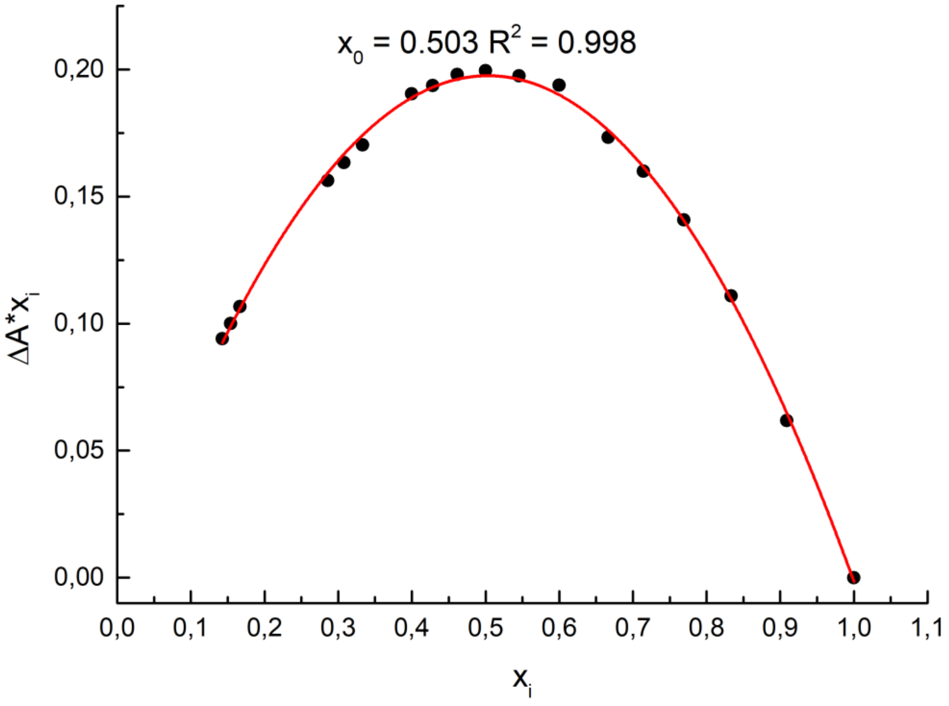

Fig. S9. Job plot analysis for **2** + TBABr in CH_3CN.

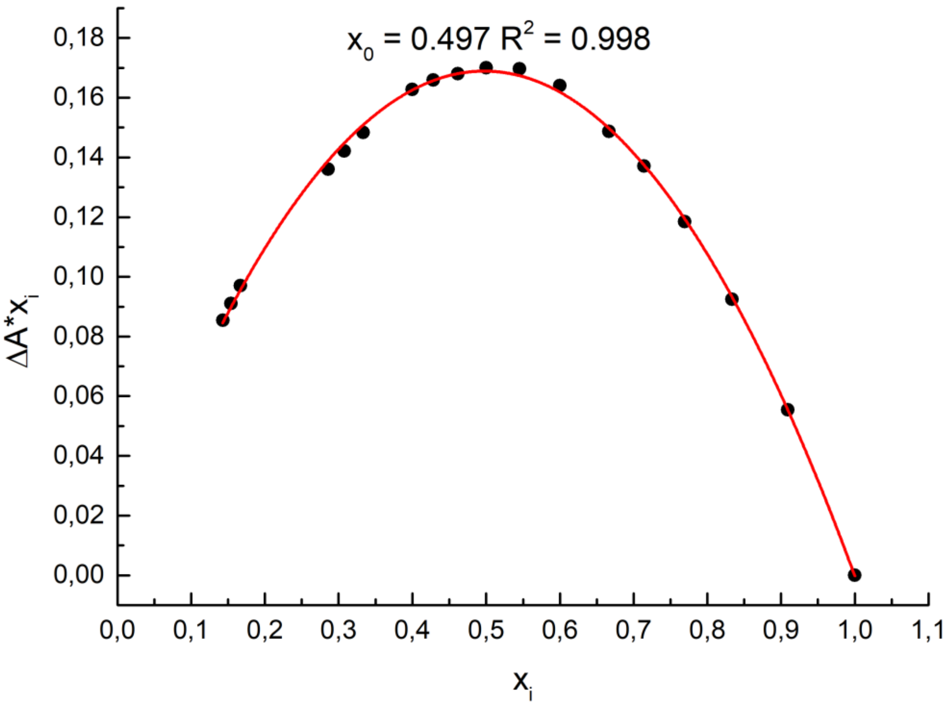

Fig. S10. Job plot analysis for **2** + $NaClO_4$ in CH_3CN.

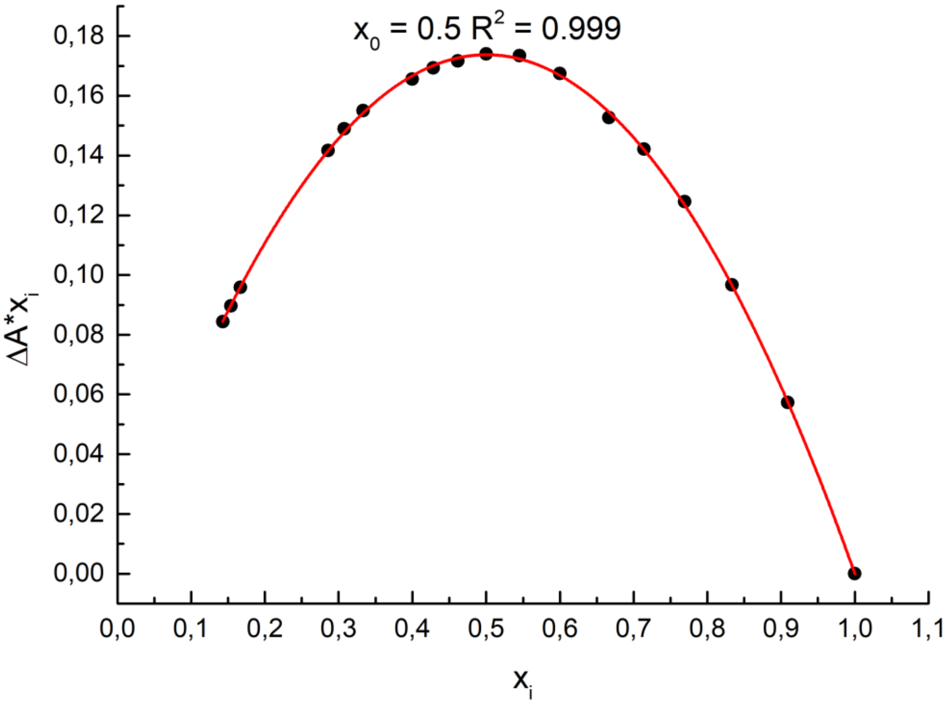

Fig. S11. Job plot analysis for **2** + KPF_6 in CH_3CN.

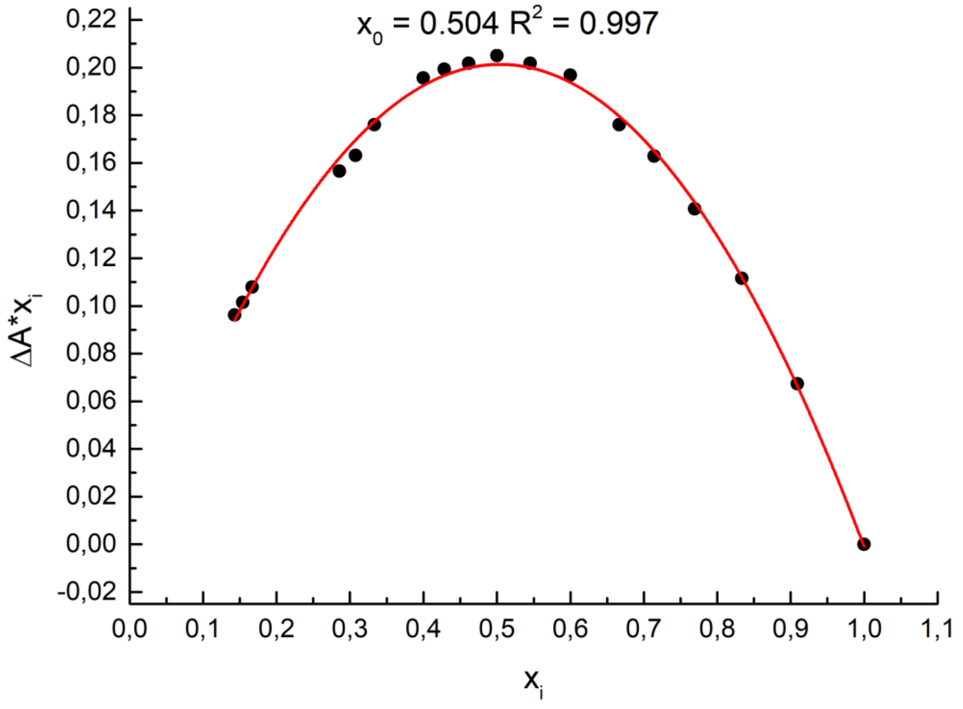

Fig. S12. Job plot analysis for **3** + TBACl in CH_3CN.

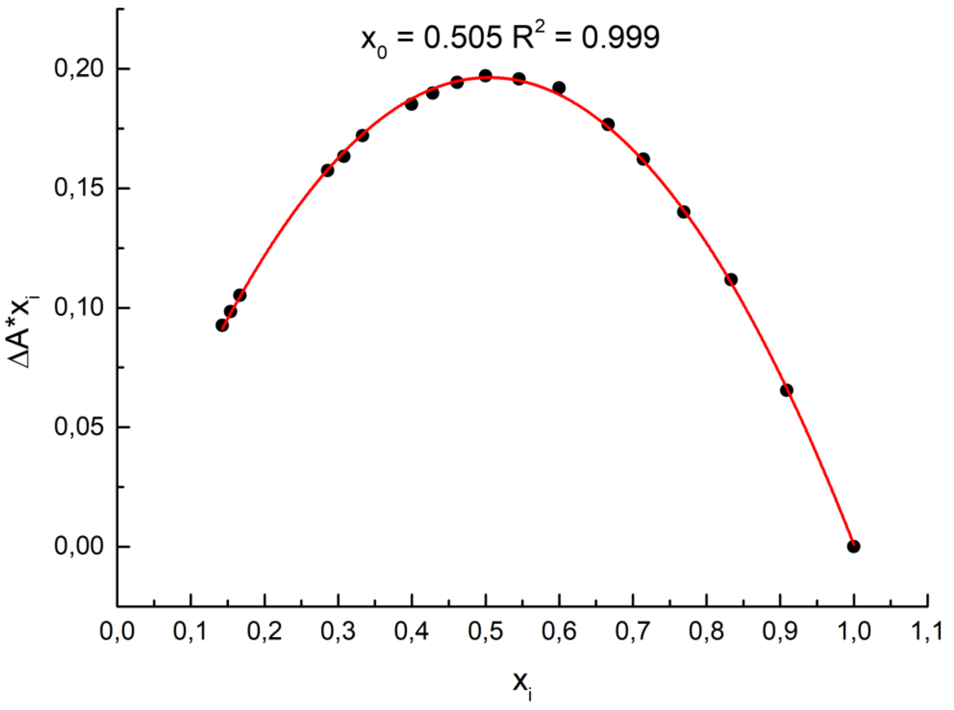

Fig. S13. Job plot analysis for **3** + TBABr in CH_3CN.

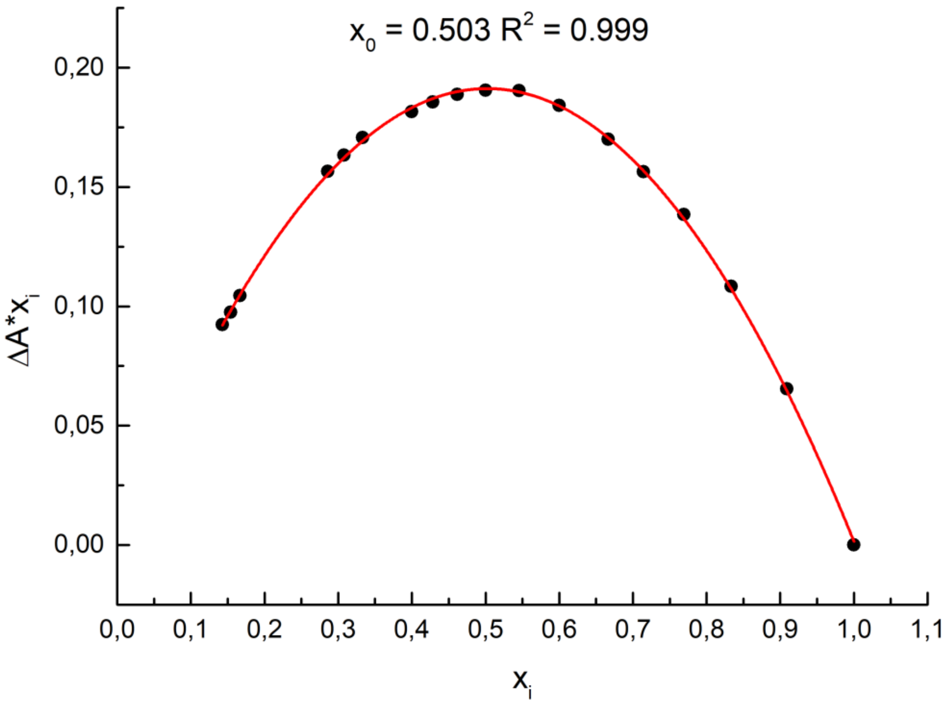

Fig. S14. UV-Vis titration of receptor **1** with TBACl and selected binding isotherms in CH_3CN.

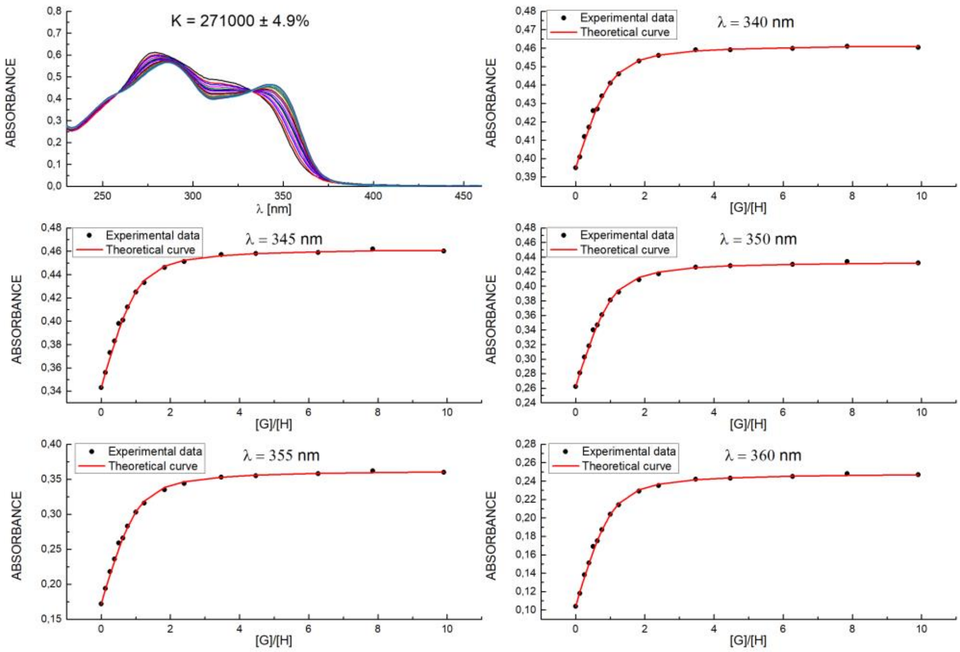

Fig. S15. UV-Vis titration of receptor **1** with TBACl in the presence of 1 equivalent of $NaClO_4$ and selected binding isotherms in CH_3CN.

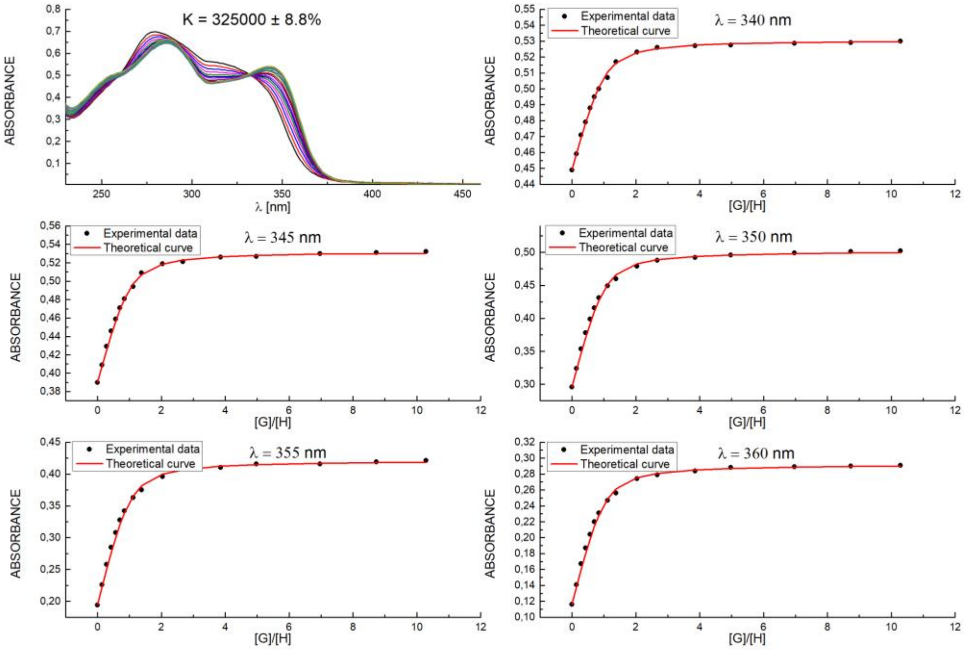

Fig. S16a. UV-Vis titration of receptor **2** with TBACl and selected binding isotherms in CH_3CN.

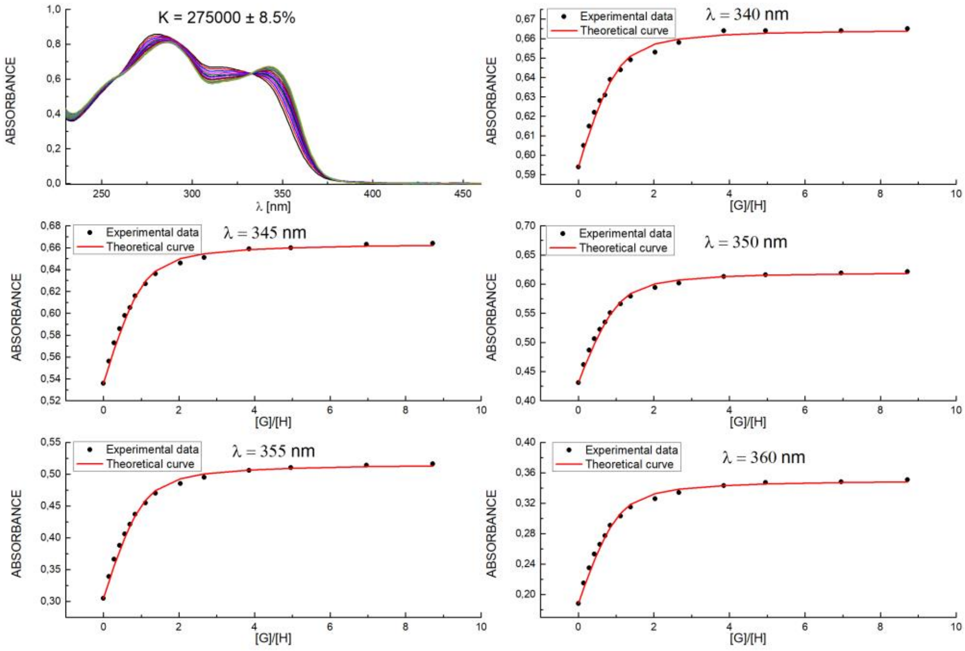

Fig. S16b. UV-Vis titration of receptor **2** with TBACl in the presence of 1 equivalent of $NaClO_4$ and selected binding isotherms in CH_3CN.

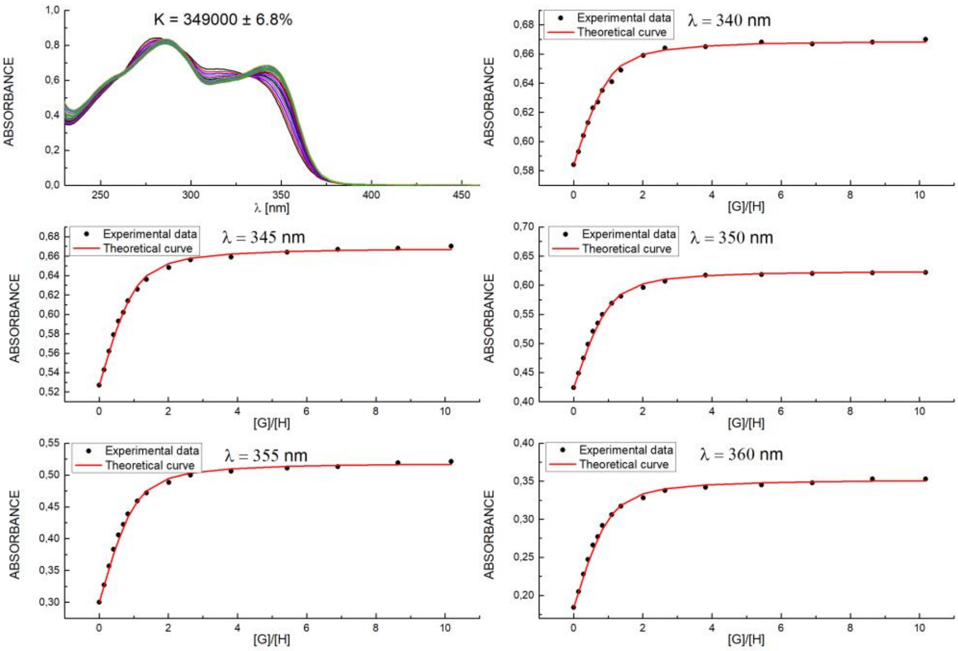

Fig. S17. UV-Vis titration of receptor **2** with TBACl in the presence of 1 equivalent of KPF_6 and selected binding isotherms in CH_3CN.

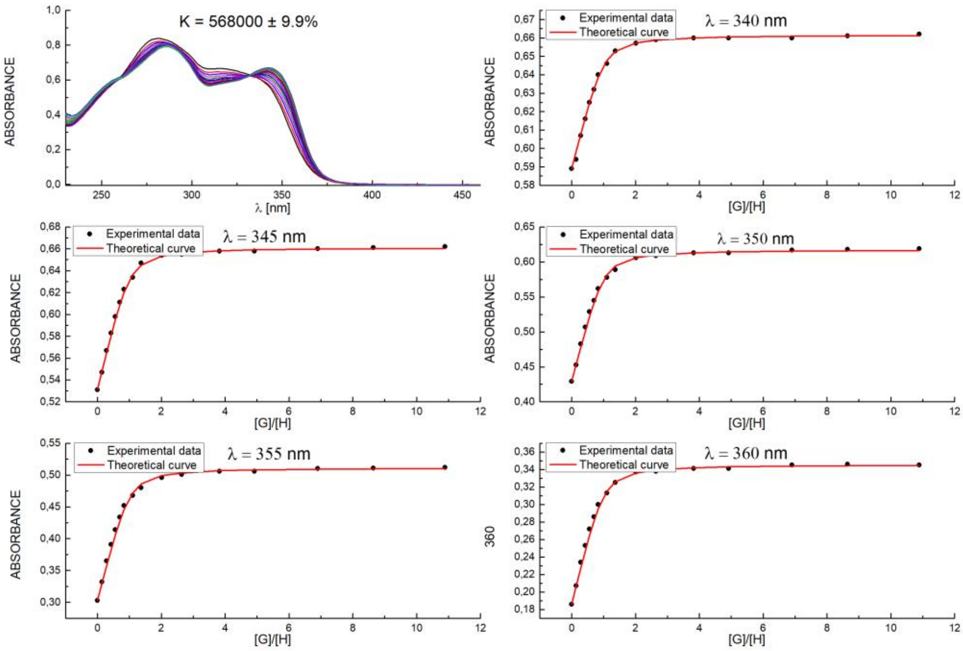

Fig. S18. UV-Vis titration of receptor **2** with TBABr and selected binding isotherms in CH_3CN.

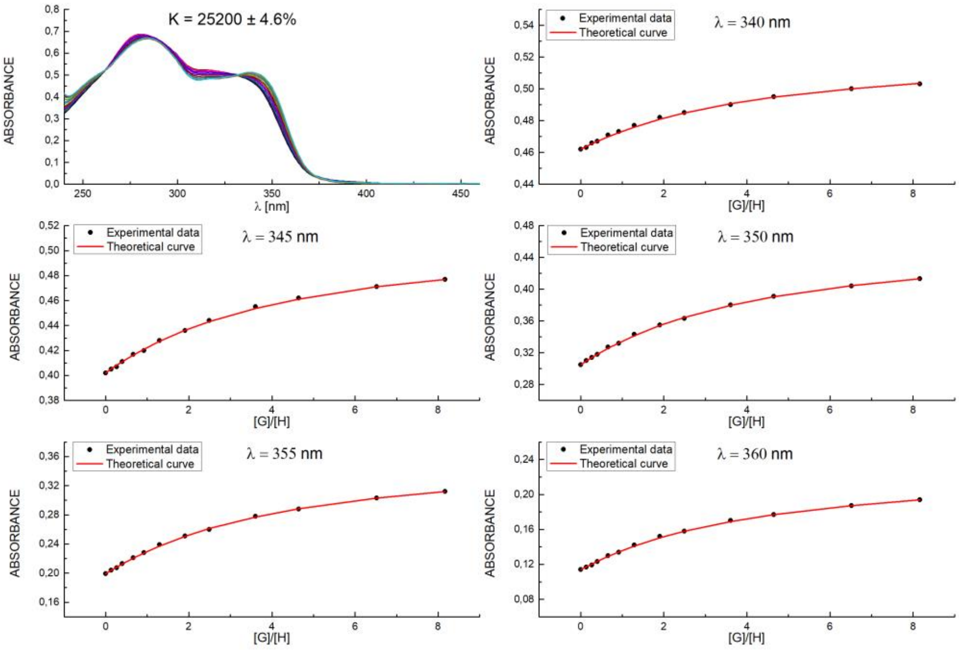

Fig. S19. UV-Vis titration of receptor **2** with TBABr in the presence of 1 equivalent of $NaClO_4$ and selected binding isotherms in CH_3CN.

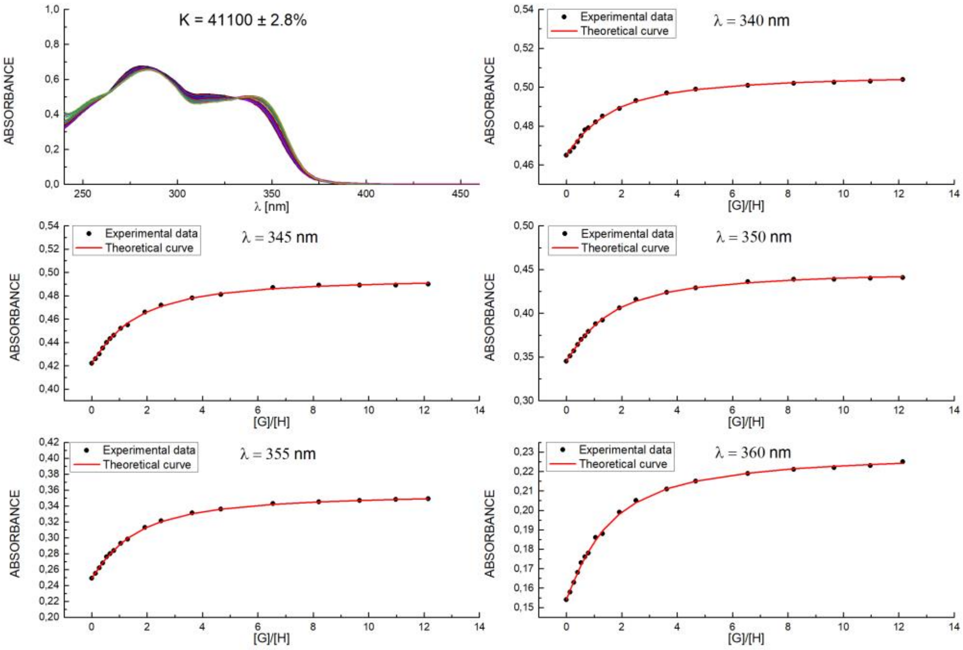

Fig. S20. UV-Vis titration of receptor **2** with TBABr in the presence of 1 equivalent of KPF_6 and selected binding isotherms in CH_3CN.

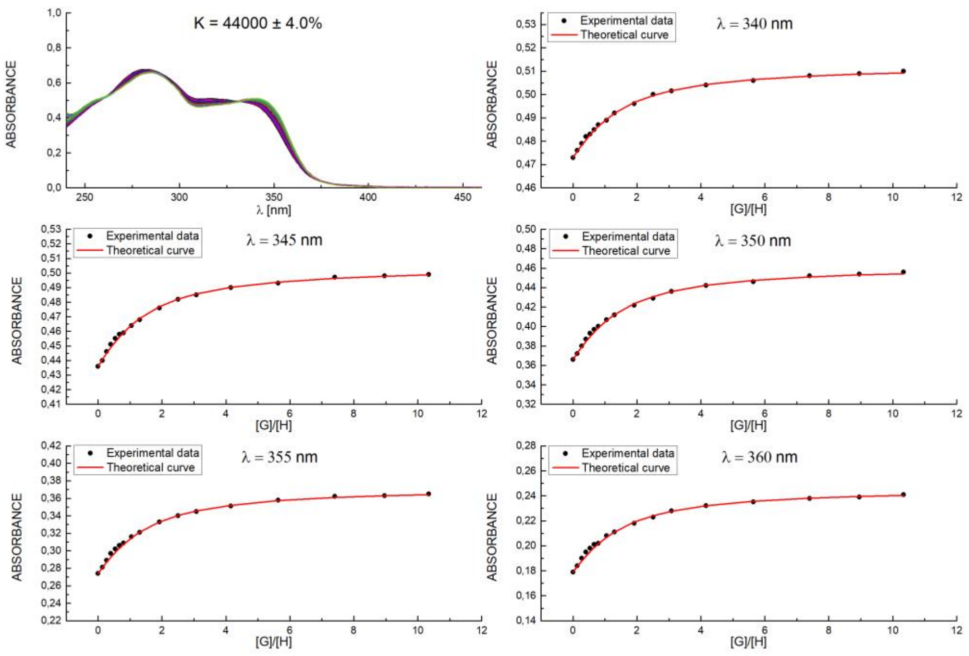

Fig. S21. UV-Vis titration of receptor **2** with $TBANO_2$ and selected binding isotherms in CH_3CN.

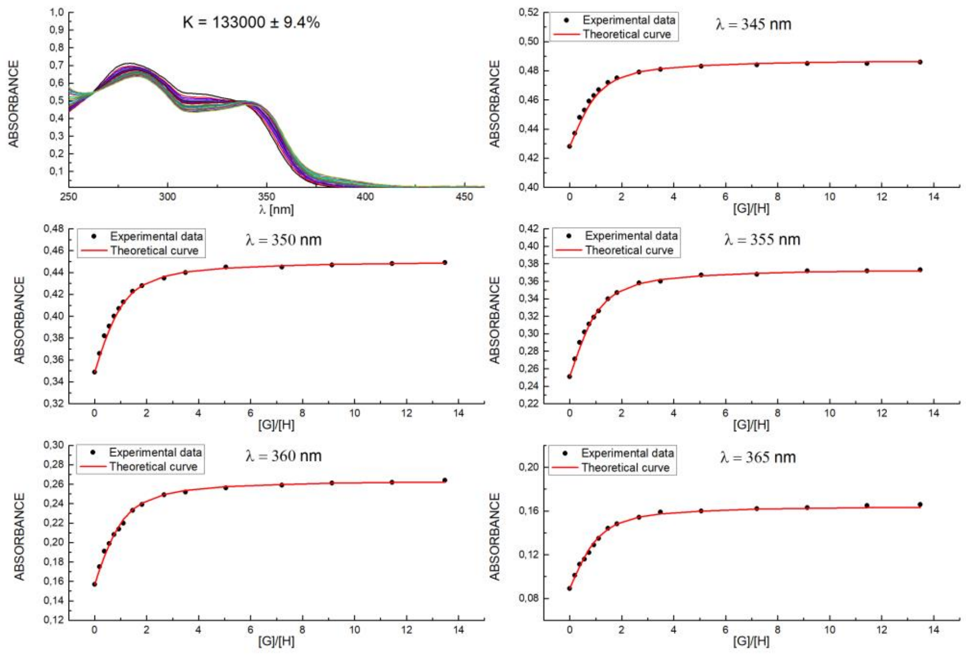

Fig. S22. UV-Vis titration of receptor **2** with $TBANO_2$ in the presence of 1 equivalent of $NaClO_4$ and selected binding isotherms in CH_3CN.

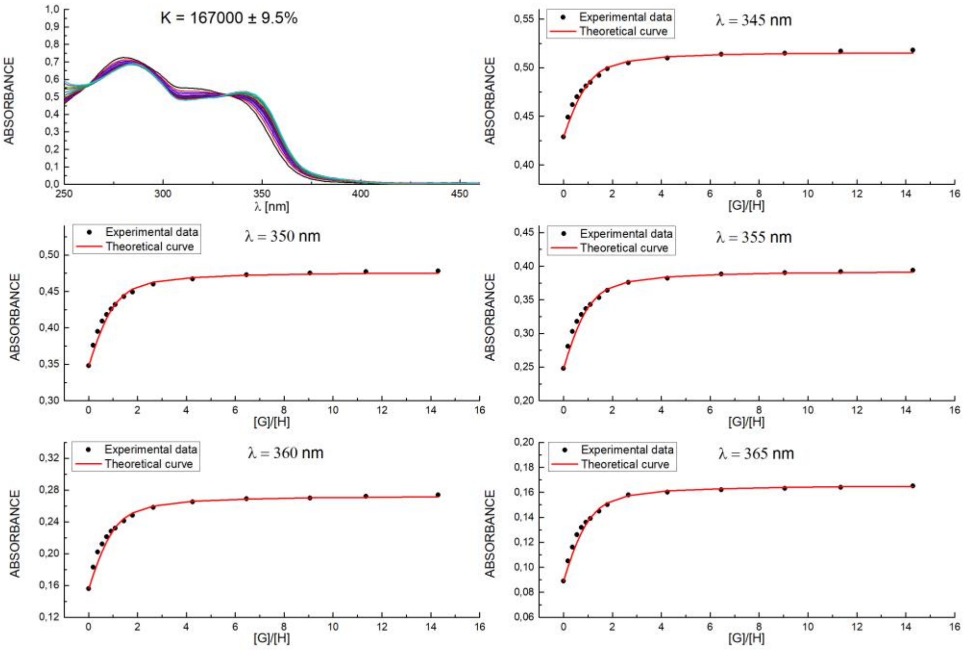

Fig. S23. UV-Vis titration of receptor **2** with $TBANO_2$ in the presence of 1 equivalent of KPF_6 and selected binding isotherms in CH_3CN.

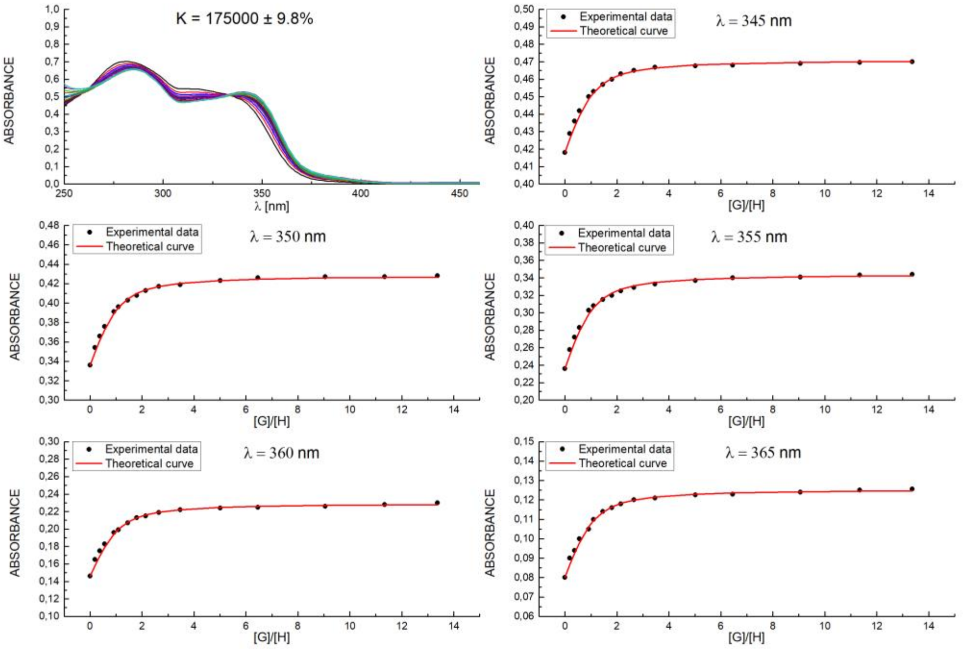

Fig. S24. UV-Vis titration of receptor **2** with $TBANO_3$ and selected binding isotherms in CH_3CN.

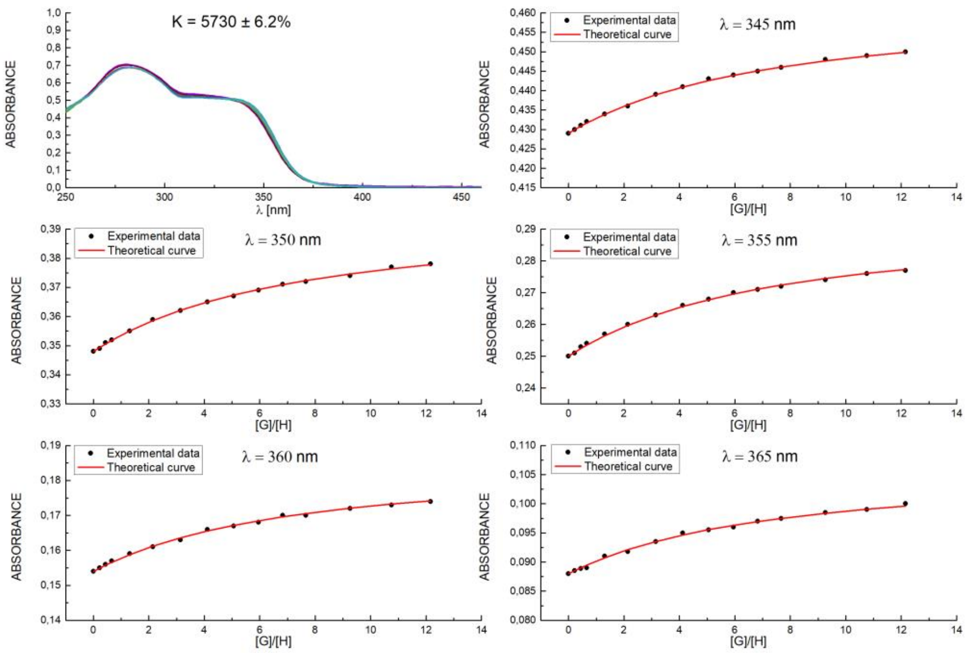

Fig. S25. UV-Vis titration of receptor **2** with $TBANO_3$ in the presence of 1 equivalent of $NaClO_4$ and selected binding isotherms in CH_3CN.

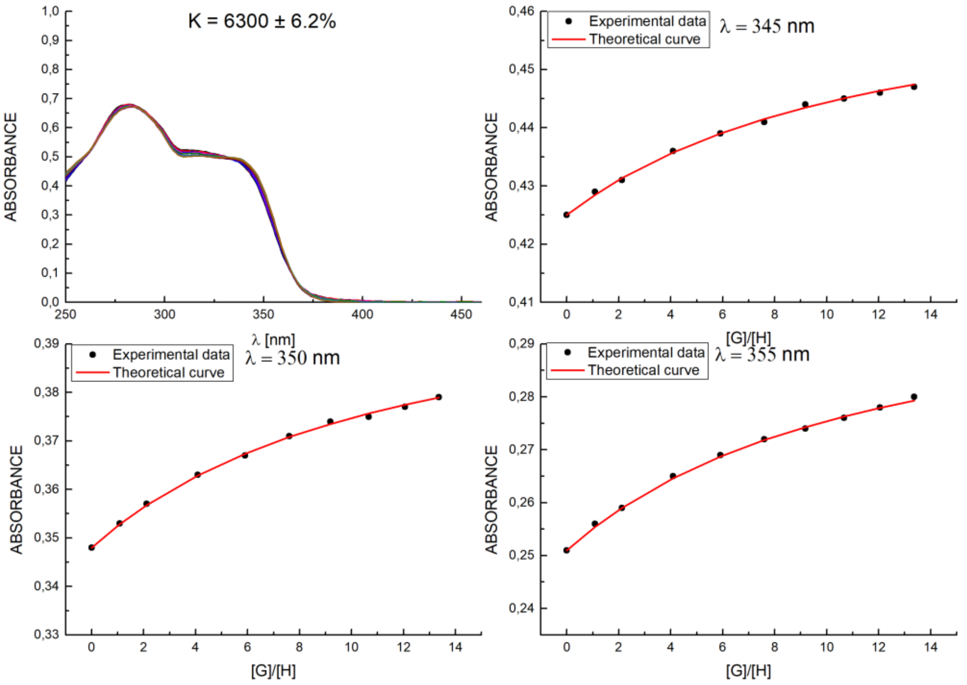

Fig. S26. UV-Vis titration of receptor **2** with $TBANO_3$ in the presence of 1 equivalent of KPF_6 and selected binding isotherms in CH_3CN.

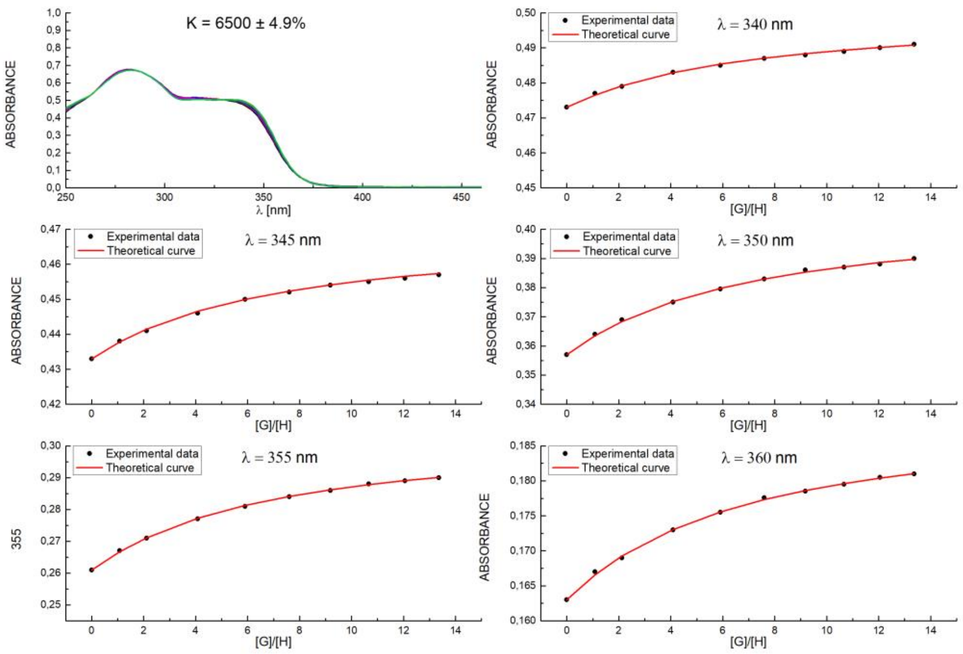

Fig. S27. UV-Vis titration spectra of receptor **2** with TBAOH in CH_3CN.

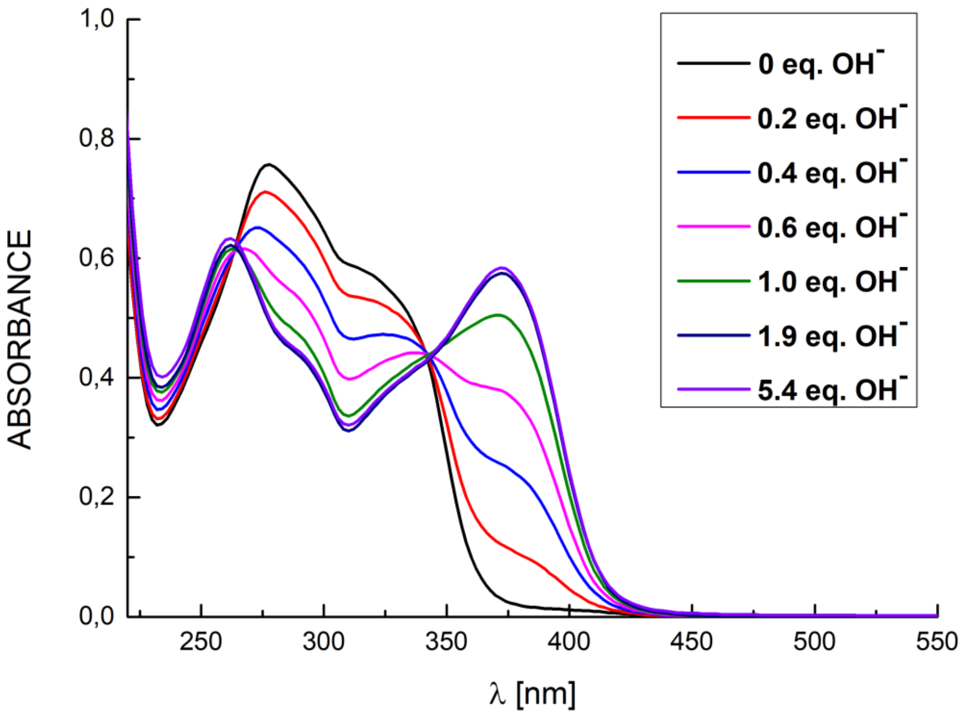

Fig. S28. UV-Vis titration spectra of receptor **2** with TBACH3COO in CH_3CN.

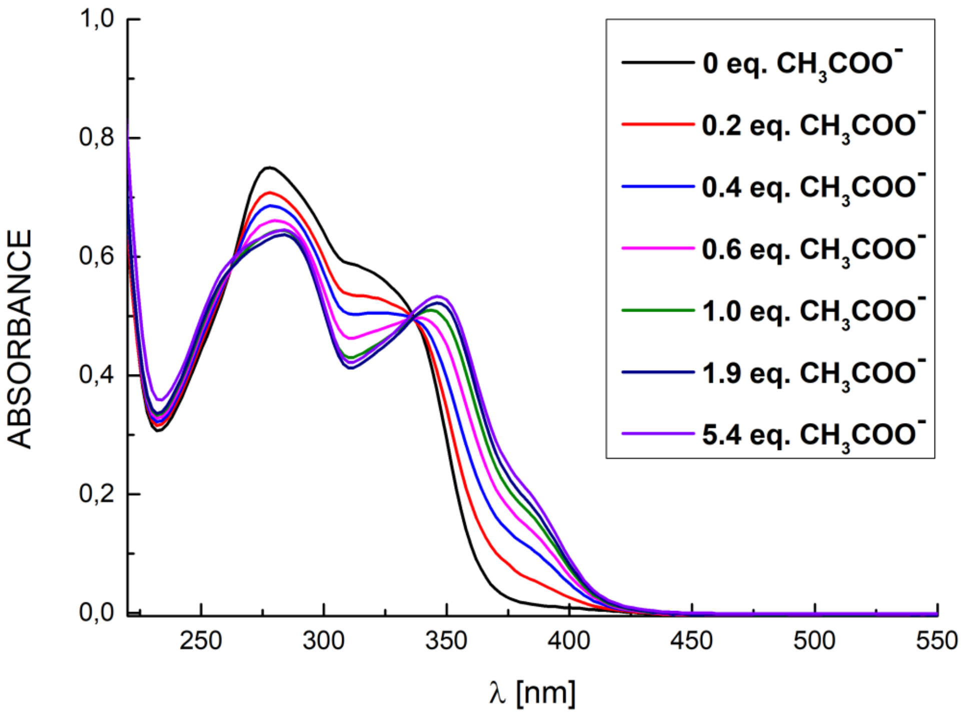

Fig. S29. UV-Vis titration spectra of receptor **2** with TBAPhCOO in CH_3CN.

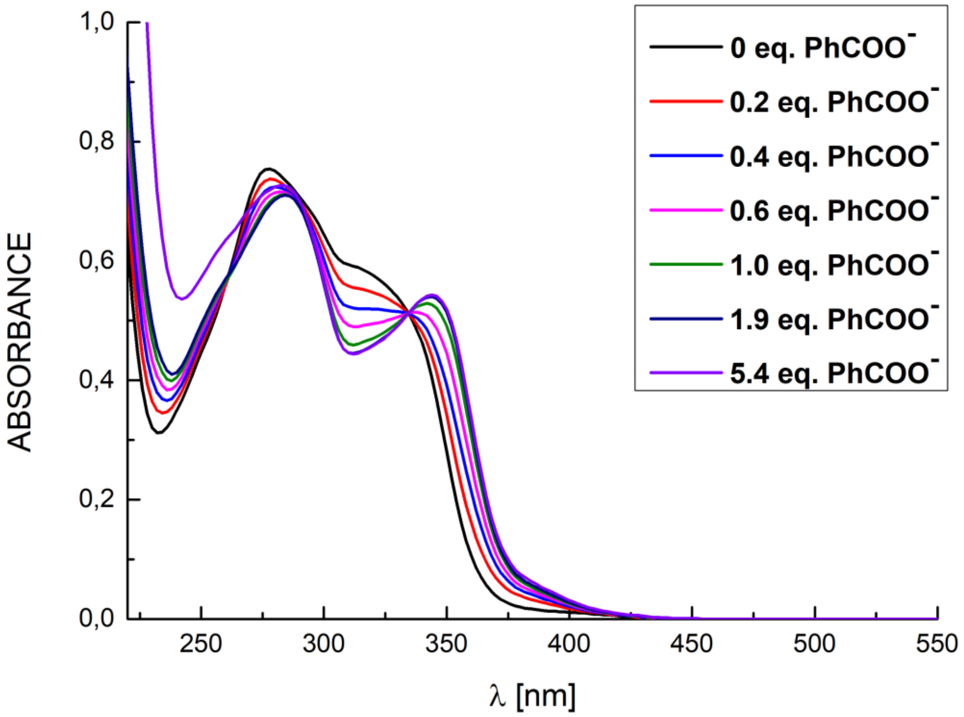

Fig. S30. UV-Vis titration of receptor **2** with TBACl in 0.5% H_2O in CH_3CN and selected binding isotherms.

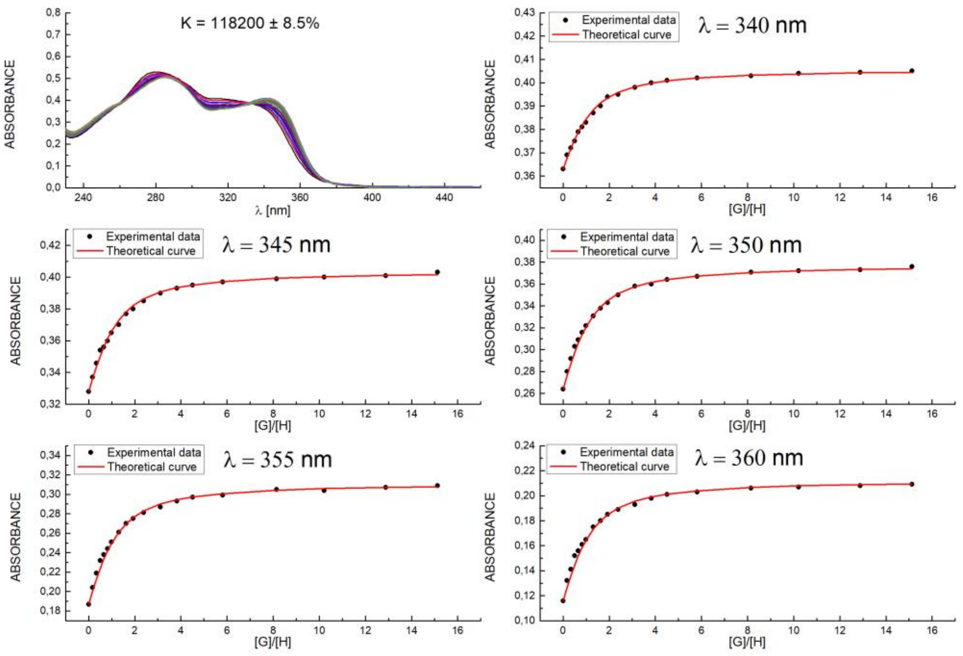

Fig. S31. UV-Vis titration of receptor **2** with TBACl in 0.5% H_2O in CH_3CN in the presence of 1 equivalent of $NaClO_4$ and selected binding isotherms.

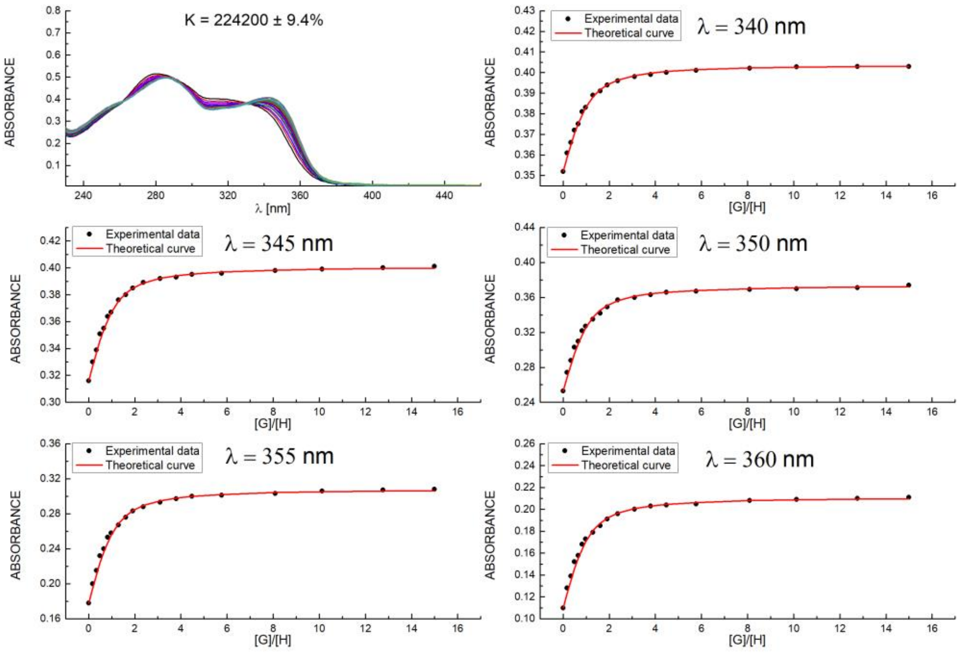

Fig. S32. UV-Vis titration of receptor **2** with TBACl in 0.5% H_2O in CH_3CN in the presence of 1 equivalent of KPF_6 and selected binding isotherms.

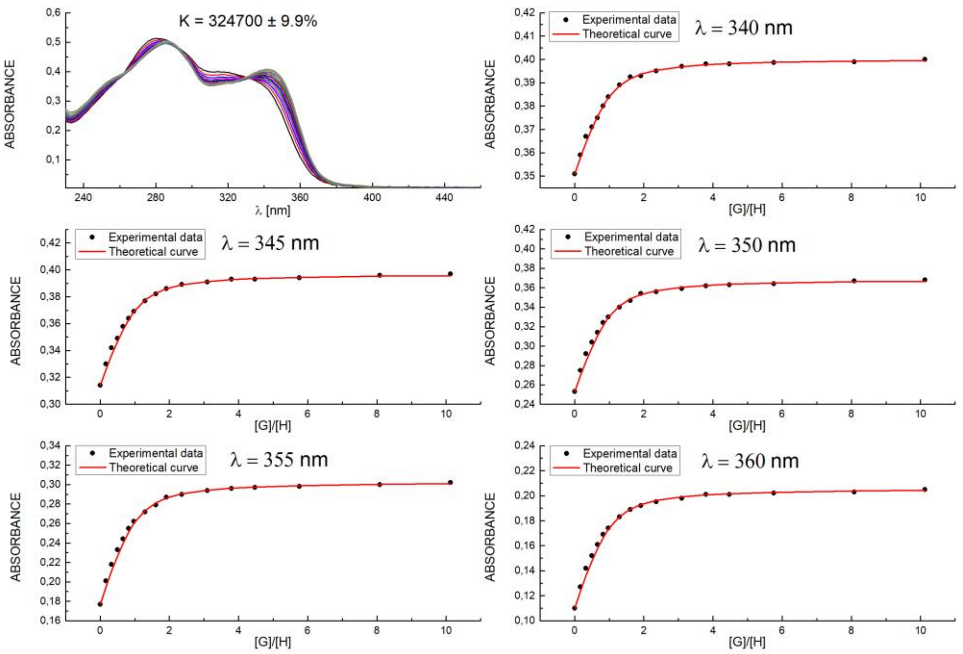

Fig. S33. UV-Vis titration of receptor **2** with TBACl in 1% H_2O in CH_3CN and selected binding isotherms.

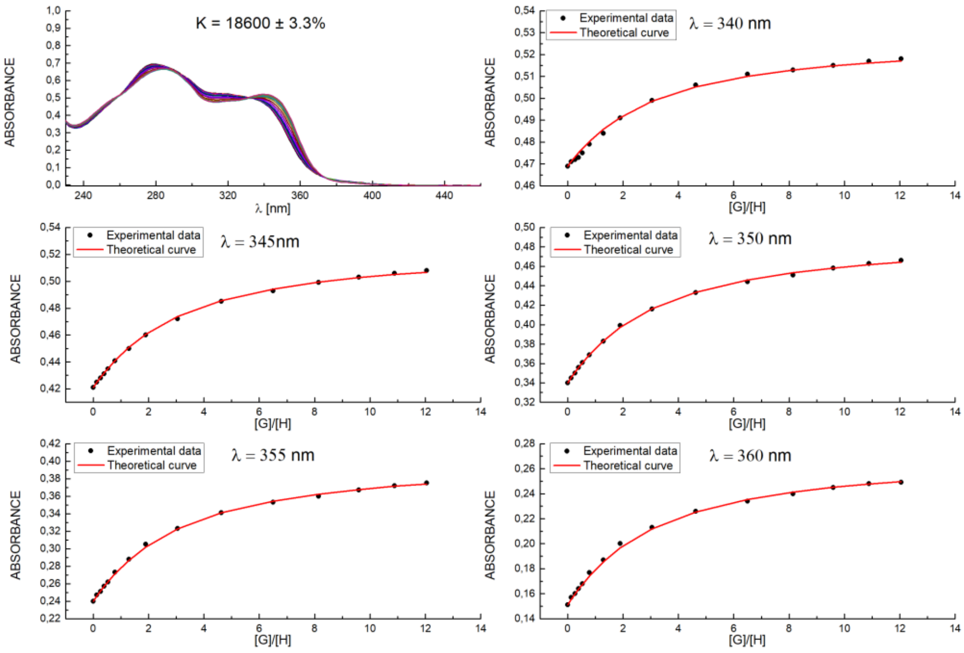

Fig. S34. UV-Vis titration of receptor **2** with TBACl in 1% H_2O in CH_3CN in the presence of 1 equivalent of $NaClO_4$ and selected binding isotherms.

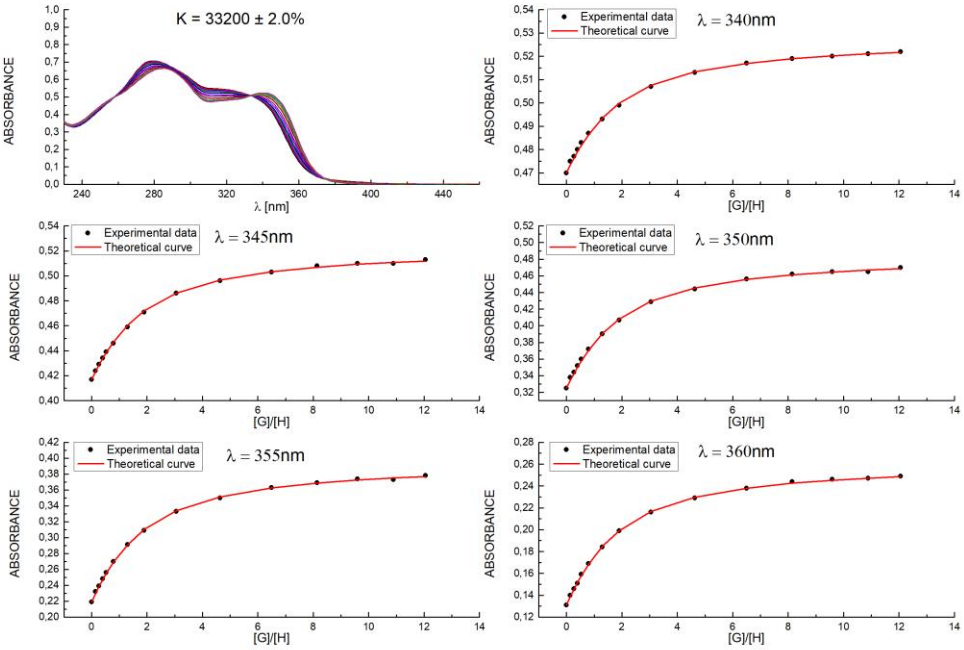

Fig. S35. UV-Vis titration of receptor **2** with TBACl in 1% H_2O in CH_3CN in the presence of 1 equivalent of KPF_6 and selected binding isotherms.

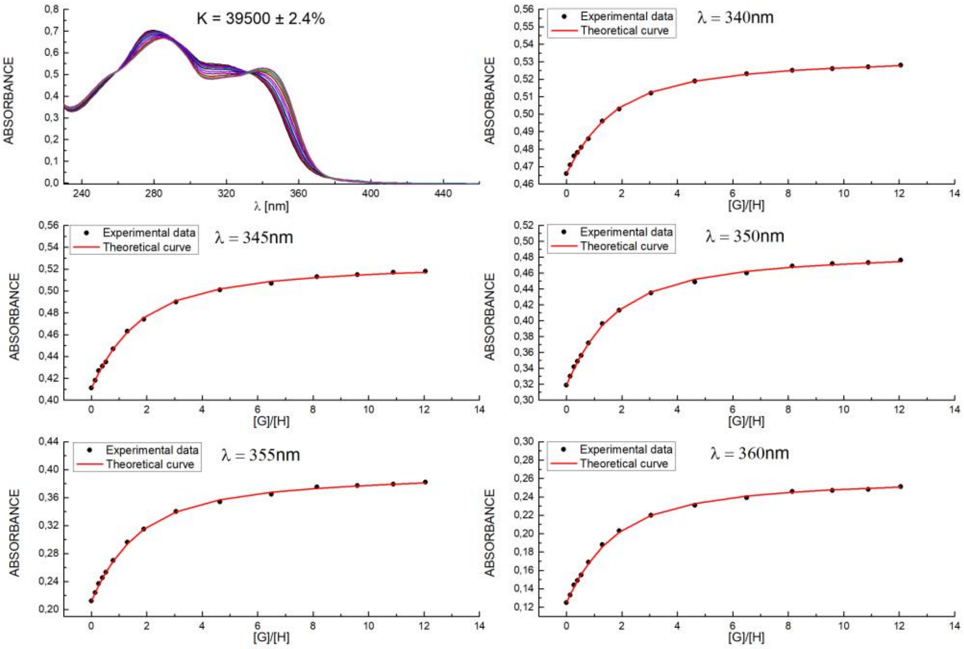

Fig. S36. UV-Vis titration of receptor **2** with NaCl in 5% H_2O in CH_3CN and selected binding isotherms.

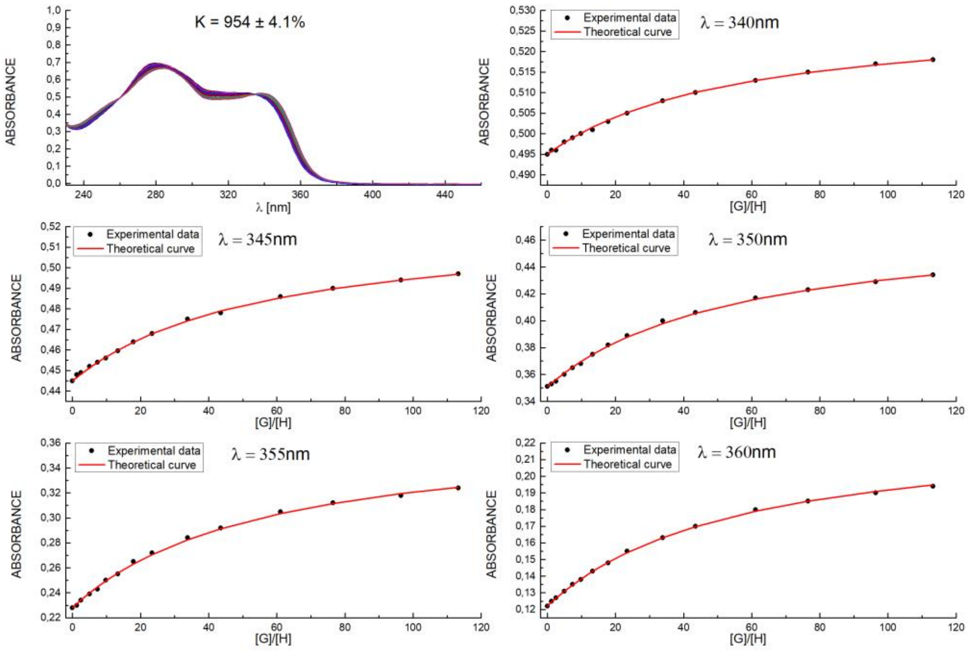

Fig. S37. UV-Vis titration of receptor **2** with KCl in 5% H_2O in CH_3CN and selected binding isotherms.

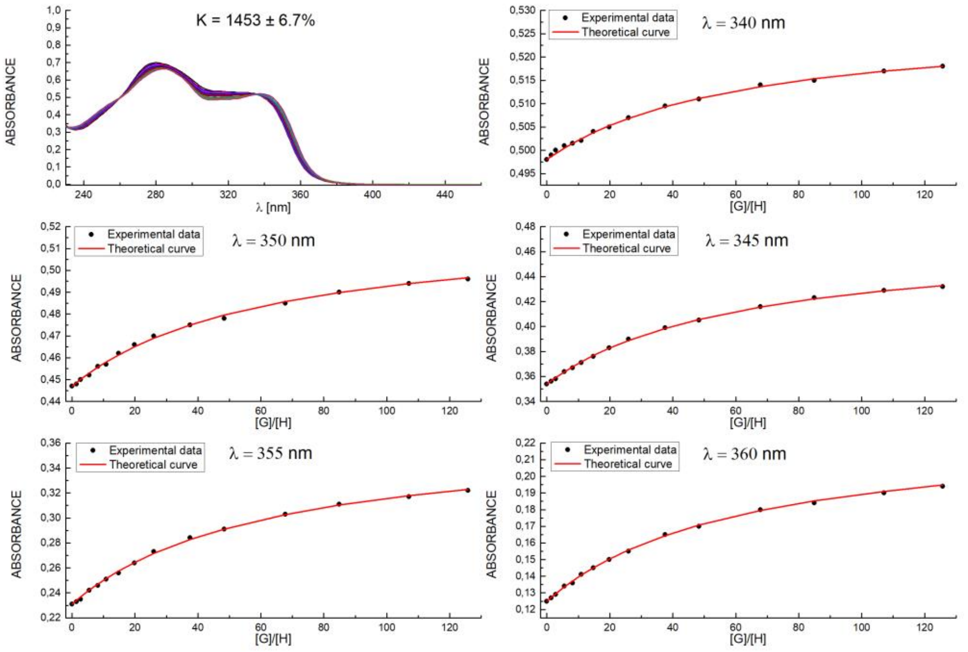

Fig. S38. UV-Vis titration of receptor **2** with TBA_2SO_4 and selected binding isotherms in CH_3CN.

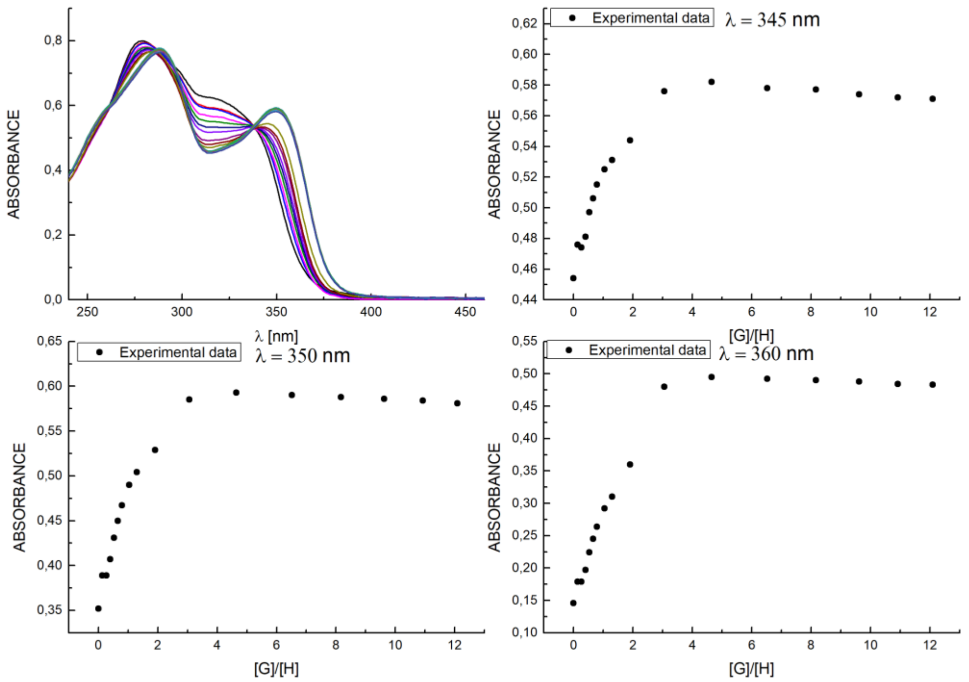

Fig. S39. UV-Vis titration of receptor **2** with TBA_2SO_4 in the presence of 1 equivalent of $NaClO_4$ and selected binding isotherms in CH_3CN.

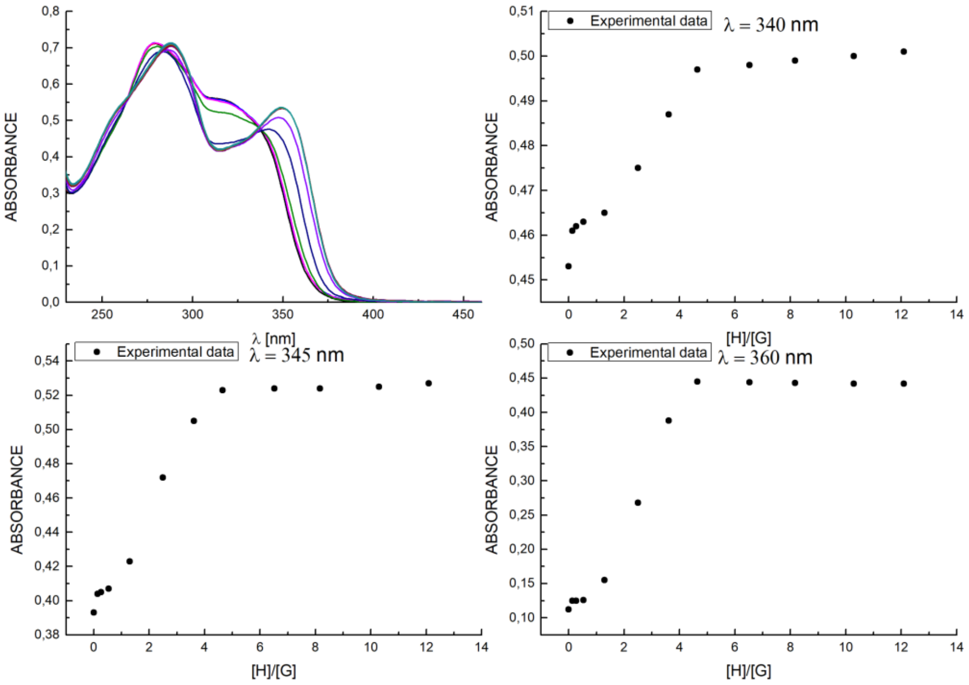

Fig. S40. UV-Vis titration of receptor **2** with TBA_2SO_4 in the presence of 1 equivalent of KPF_6 and selected binding isotherms in CH_3CN.

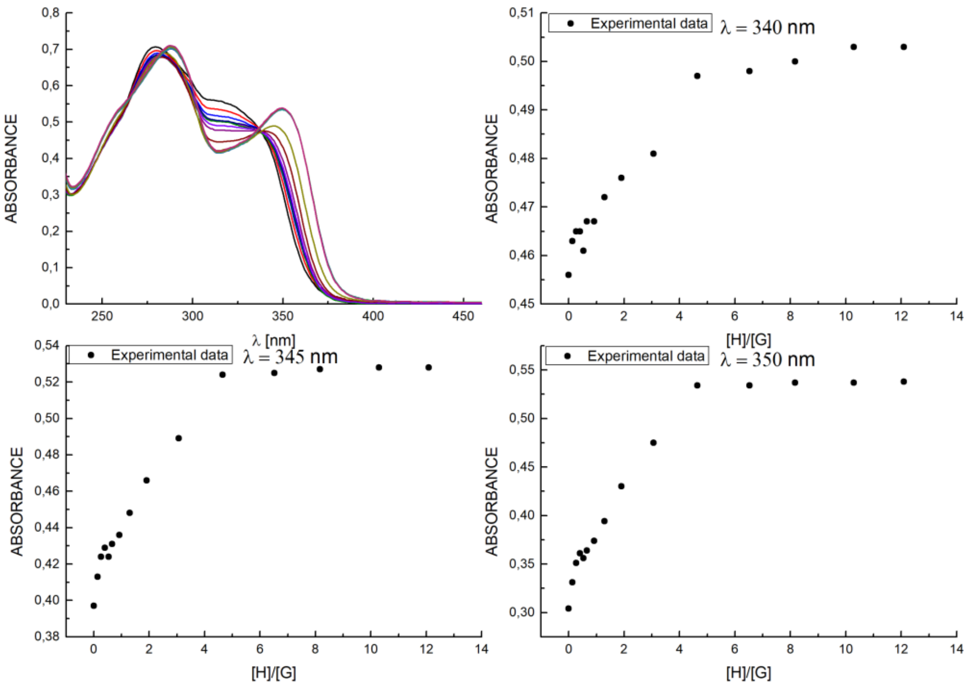

Fig. S41. UV-Vis titration of receptor **3** with TBACl and selected binding isotherms in CH_3CN.

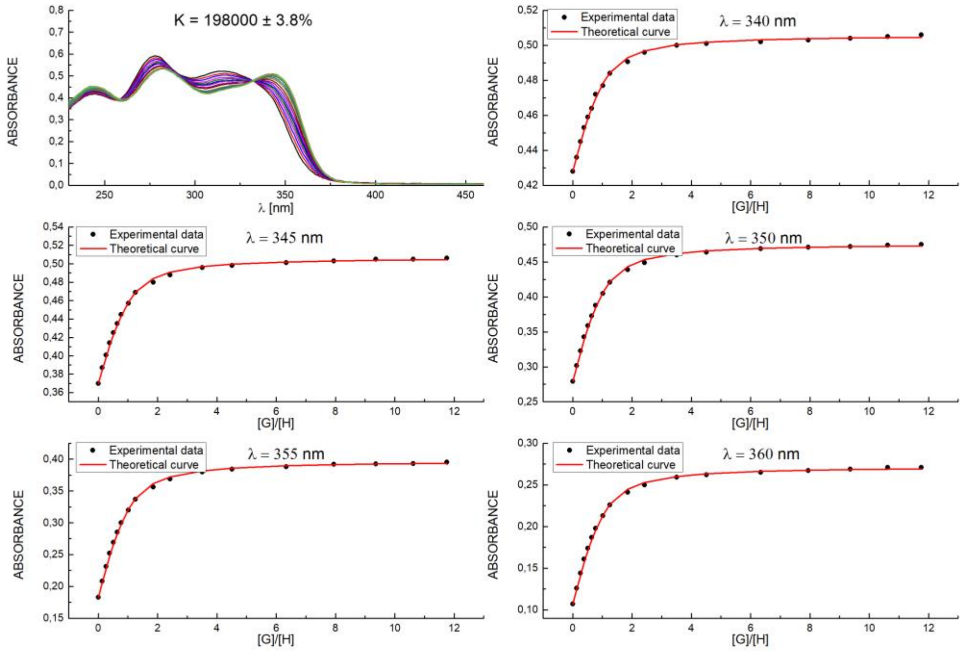

Fig. S42. UV-Vis titration of receptor **3** with TBACl in the presence of 1 equivalent of $NaClO_4$ and selected binding isotherms and selected binding isotherms in CH_3CN.

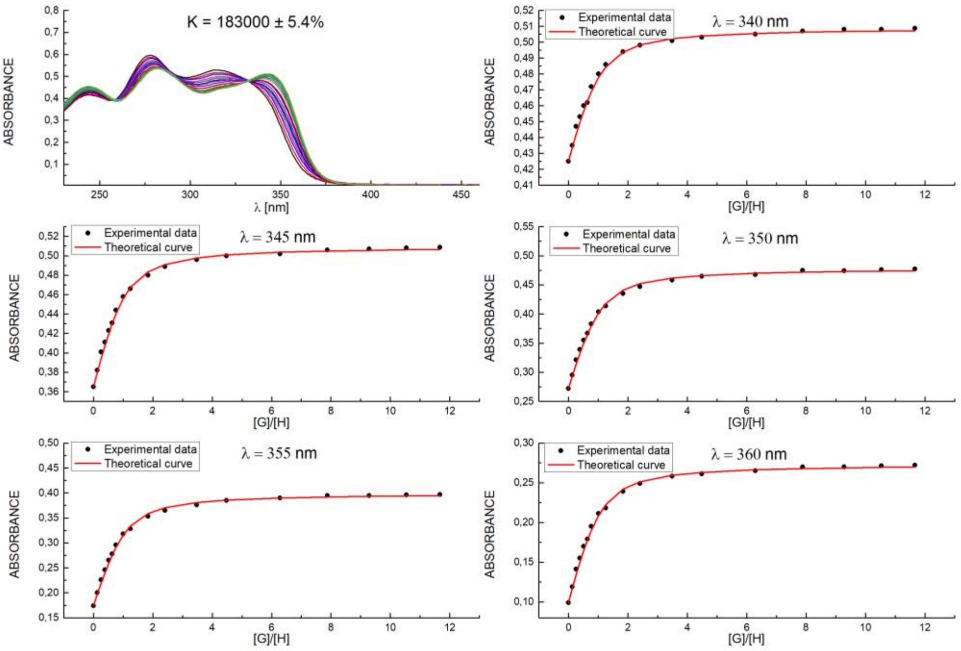

Fig. S43. UV-Vis titration of receptor **3** with TBACl in the presence of 1 equivalent of KPF_6 and selected binding isotherms and selected binding isotherms in CH_3CN.

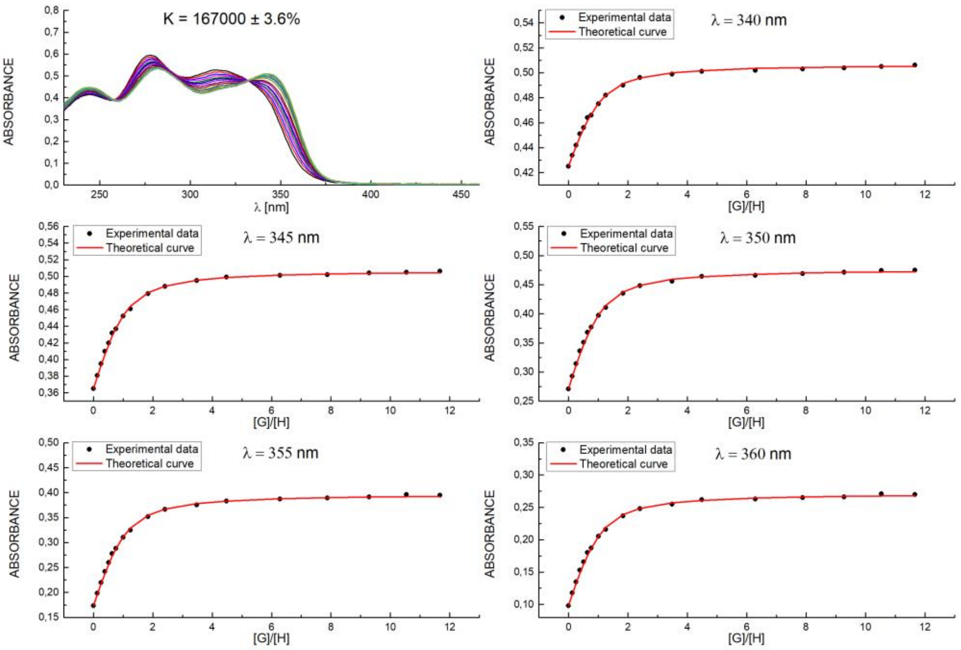

Fig. S44. UV-Vis titration of compounds **S2** with TBACl and selected binding isotherms in CH_3CN.

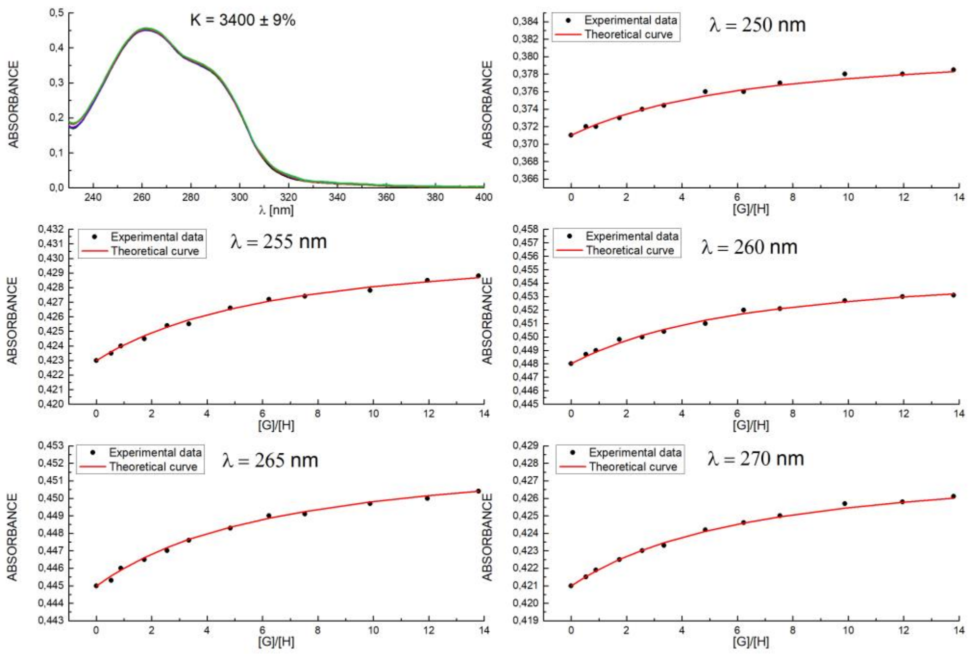

Fig. S45. UV-Vis titration of compounds **S2** with TBACl in the presence of 1 equivalent of $NaClO_4$ and selected binding isotherms and selected binding isotherms in CH_3CN.

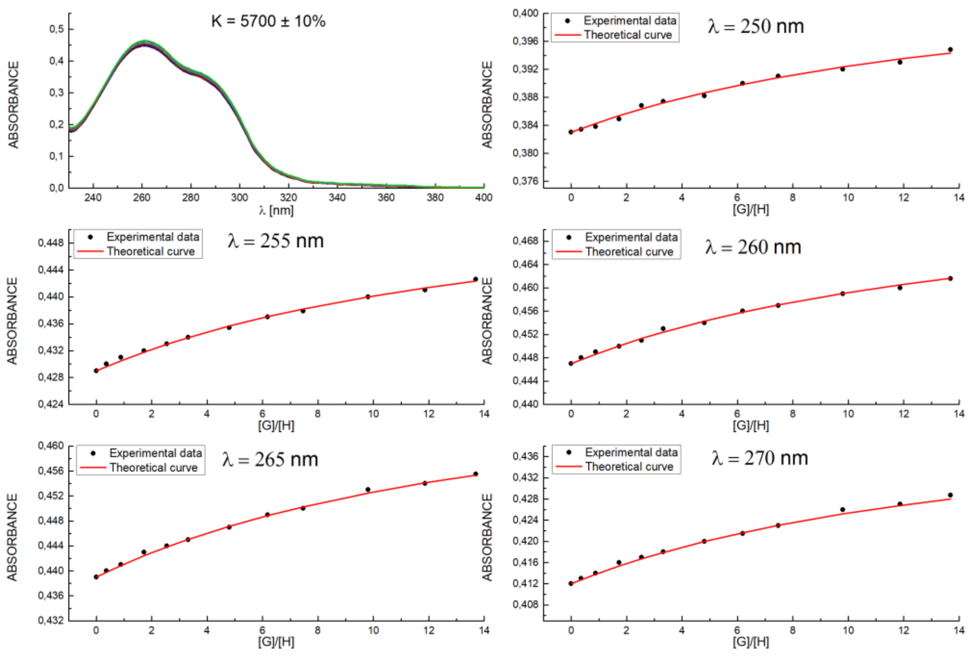

Fig. S46. UV-Vis titration of compounds **S2** with TBACl in the presence of 1 equivalent of KPF_6 and selected binding isotherms and selected binding isotherms in CH_3CN.

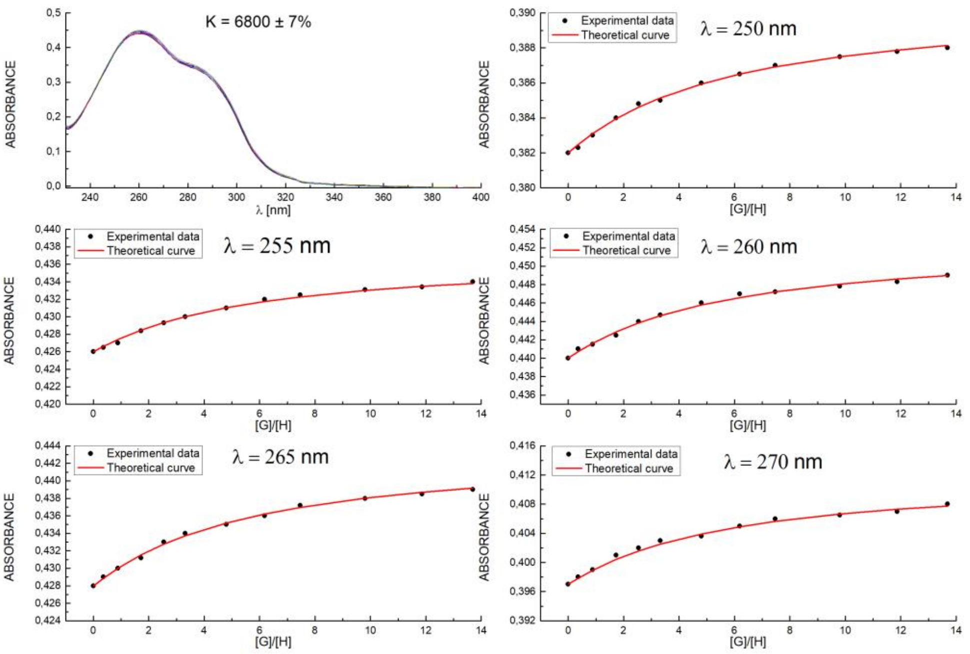

Table S1. Association constants (K_a) for interactions between receptor **2** and chloride anions and apparent association constants for interaction of receptor **2** with anions in the presence of one equivalent of sodium perchlorate or potassium hexafluorophosphate in the presence of water.[a]

**Water content [%]**	**2**	**2 + 1eq. Na⁺**	**2 + 1eq. K⁺**
**0.0**	275000 ± 8.5%	349000 ± 6.8%	568000 ± 9.9%
**0.5**	118300 ± 8.5%	224200 ± 9.4%	324700 ± 9.9%
**1.0**	18600 ± 3.3%	33200 ± 2.0%	39500 ± 2.4%

[a] UV-Vis, solvent CH_3CN, 293 K, [**2**] = $2.50 \cdot 10^{-5}$ M, chloride added as TBA salt, [TBACl] ~ 0.8 mM; M^{-1}.

2D NMR SPECTRA AND NMR TITRATION EXPERIMENTS

The ^{1}H NMR titration was performed on a Bruker 300 spectrometer, at 298K in CD_3CN. In each case, a 500 μL of freshly prepared 2.55 mM solution of receptors was added to a 5mm NMR tube. In the case of ion pair titration receptor **1, 2** and **3** was firstly pretreated with three equivalent of KPF_6 or $NaClO_4$ (refers to receptor). In the event of ion pair titration receptor **4** was firstly pretreated with one equivalent of KPF_6 or $NaClO_4$. Then small aliquots of solution of TBAX, containing receptors at constant concentration and in case of **1, 2, 3** three equivalent of KPF_6 or $NaClO_4$ and one equivalent of this salts in the case of **4**, were added and a spectrum was acquired after each addition. The resulting titration data were analyzed using BindFit (v0.5) package, available online at http://supramolecular.org

Fig. S47: ROESY spectrum of **2** in CD_3CN.

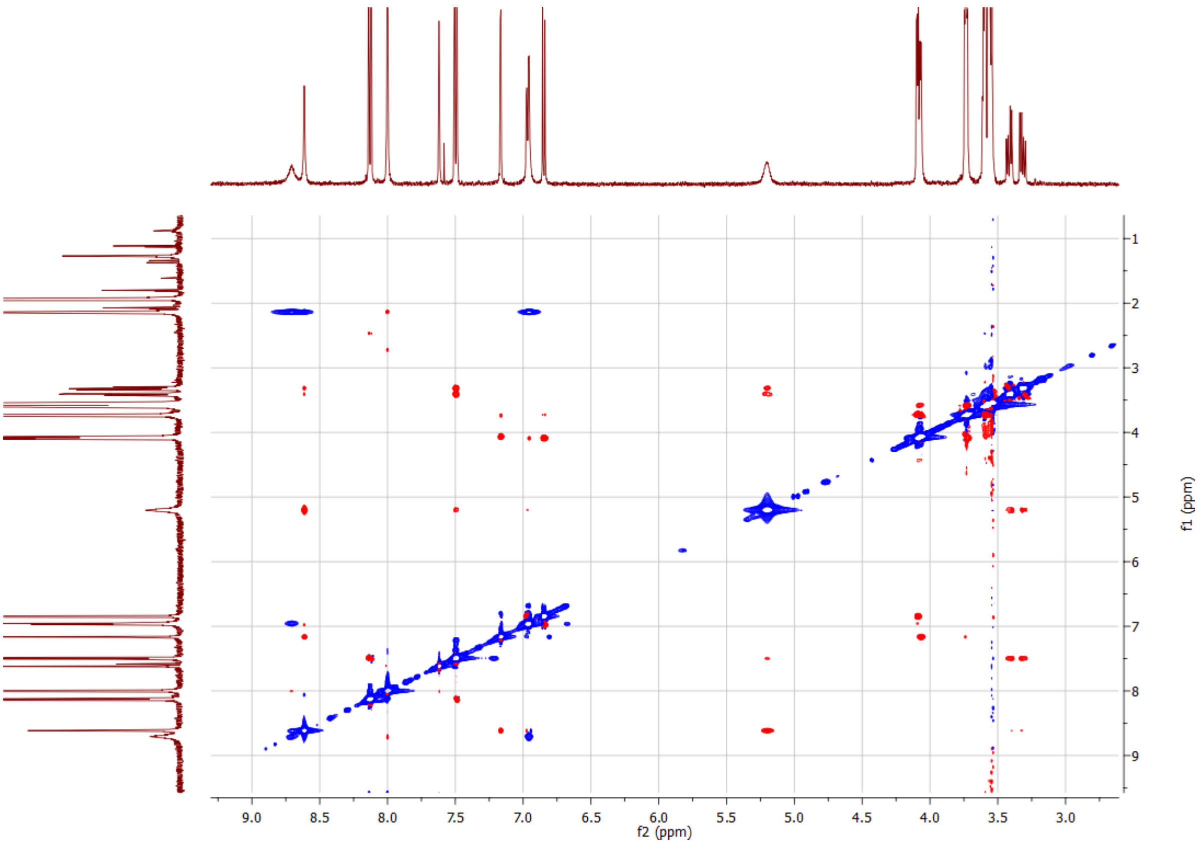

Fig. S48: HMBC spectrum of **2** in CD_3CN.

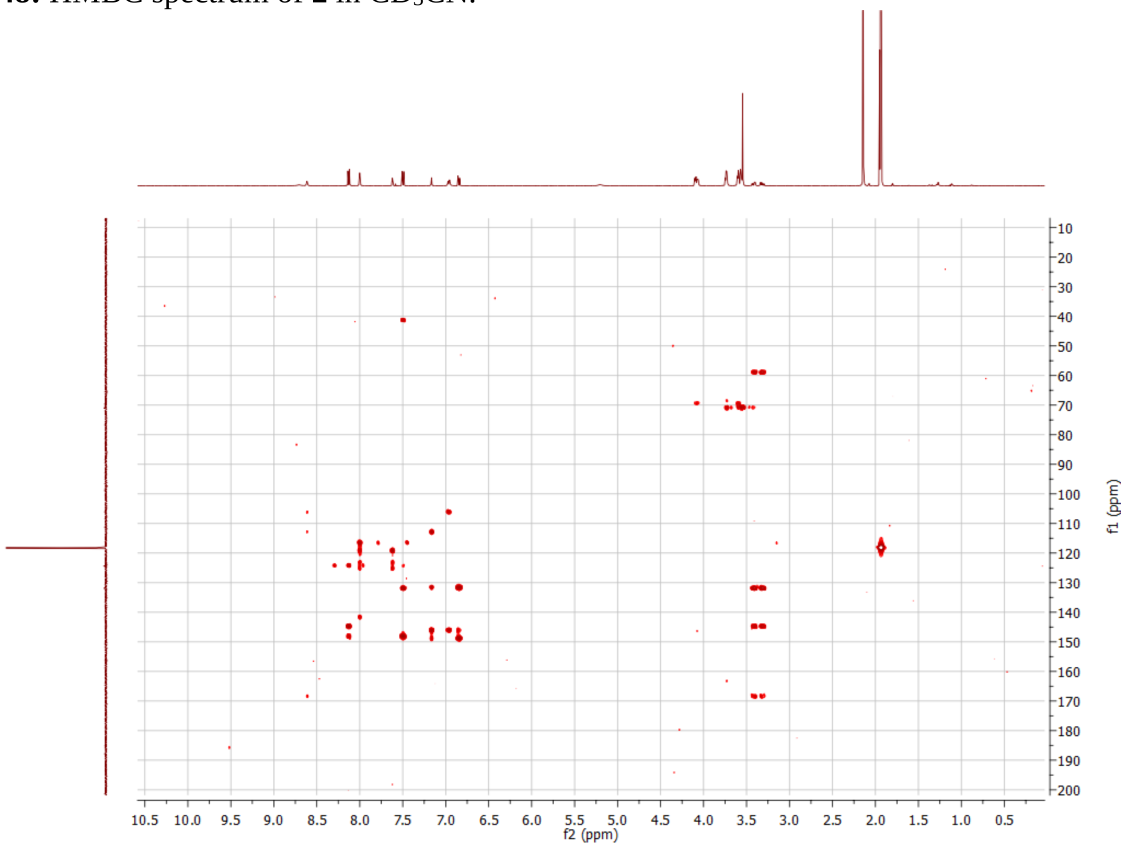

Fig. S48a. Partial ^{1}H NMR of **2** in CD_3CN along with assigned protons signals.

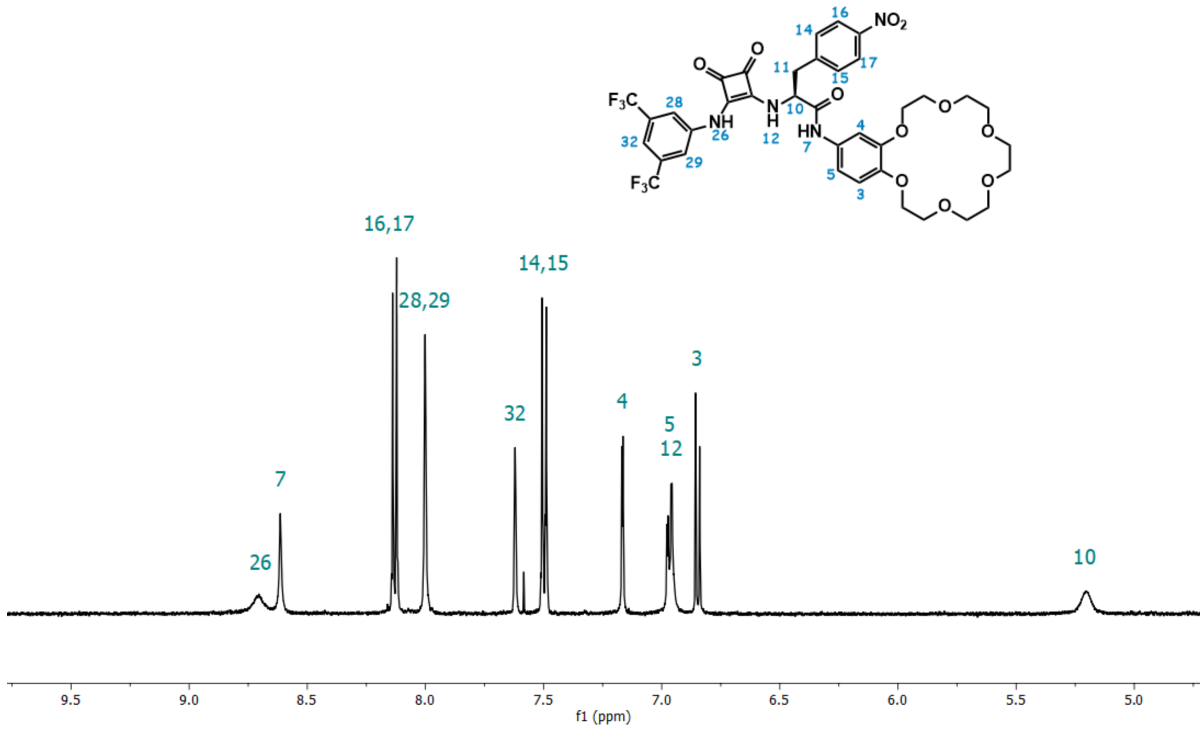

Fig. S48b. ^{1}H NMR of **2** in DMSO along with assigned carbon signals.

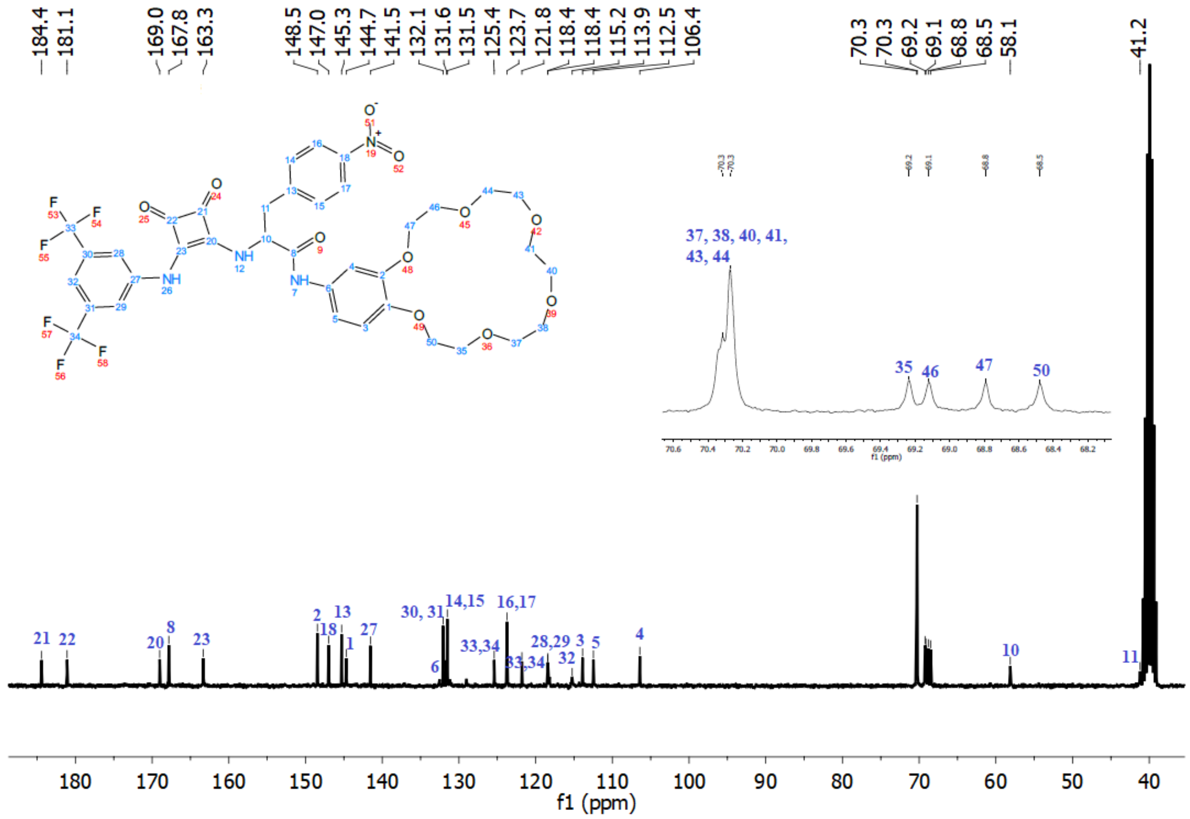

Fig. S49. ^{1}H NMR spectra recorded upon titration of receptor **4** in CD_3CN with TBACl.

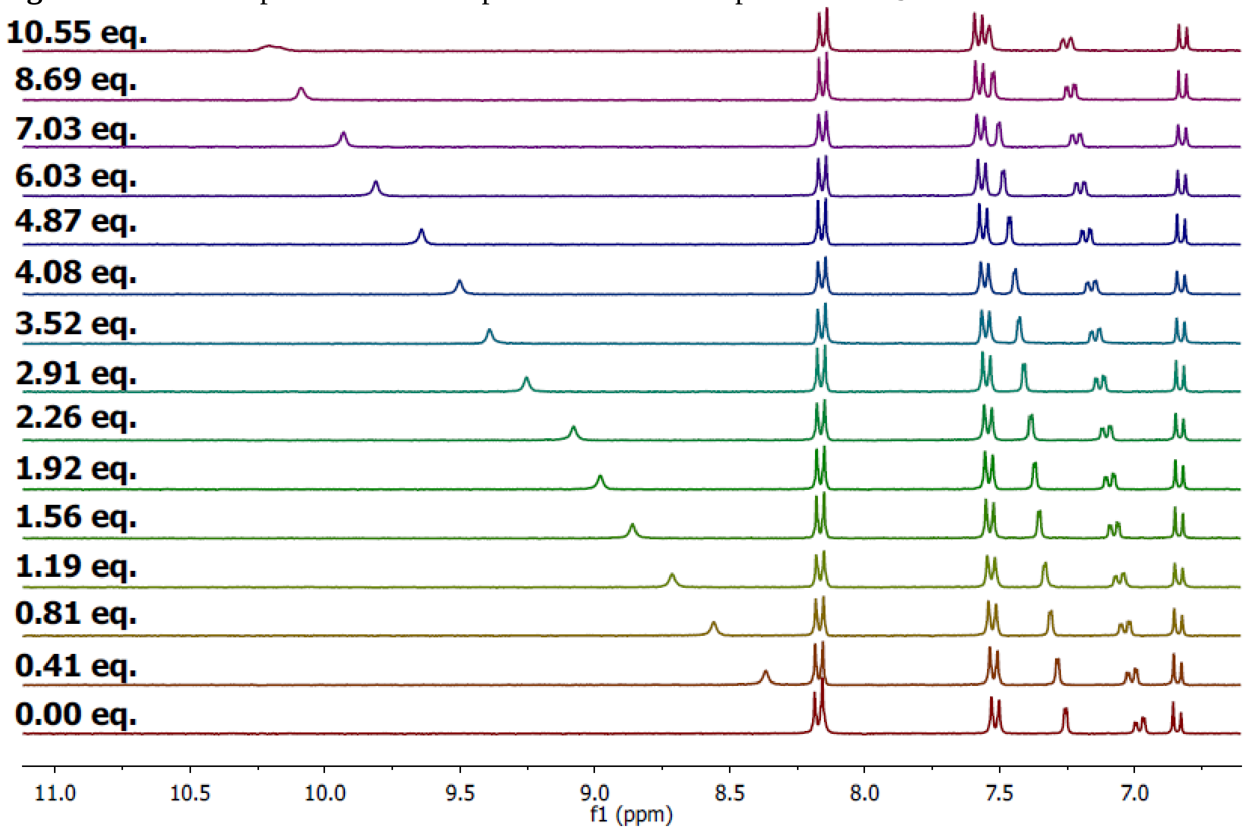

Fig. S50. ^{1}H NMR titration binding isotherms of receptor **4** ($3.11 \cdot 10^{-3}$M) in CD_3CN upon addition of increasing amounts of TBACl.

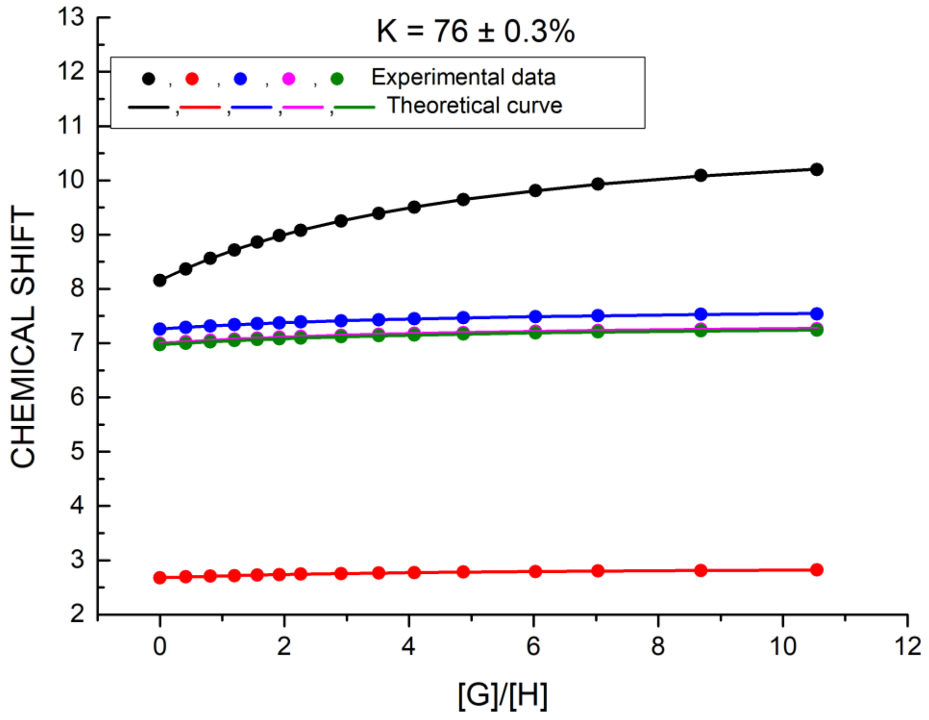

Fig. S51. ^{1}H NMR spectra recorded upon titration of receptor **4** in CD_3CN with TBACl in the presence of 1 eq. KPF_6.

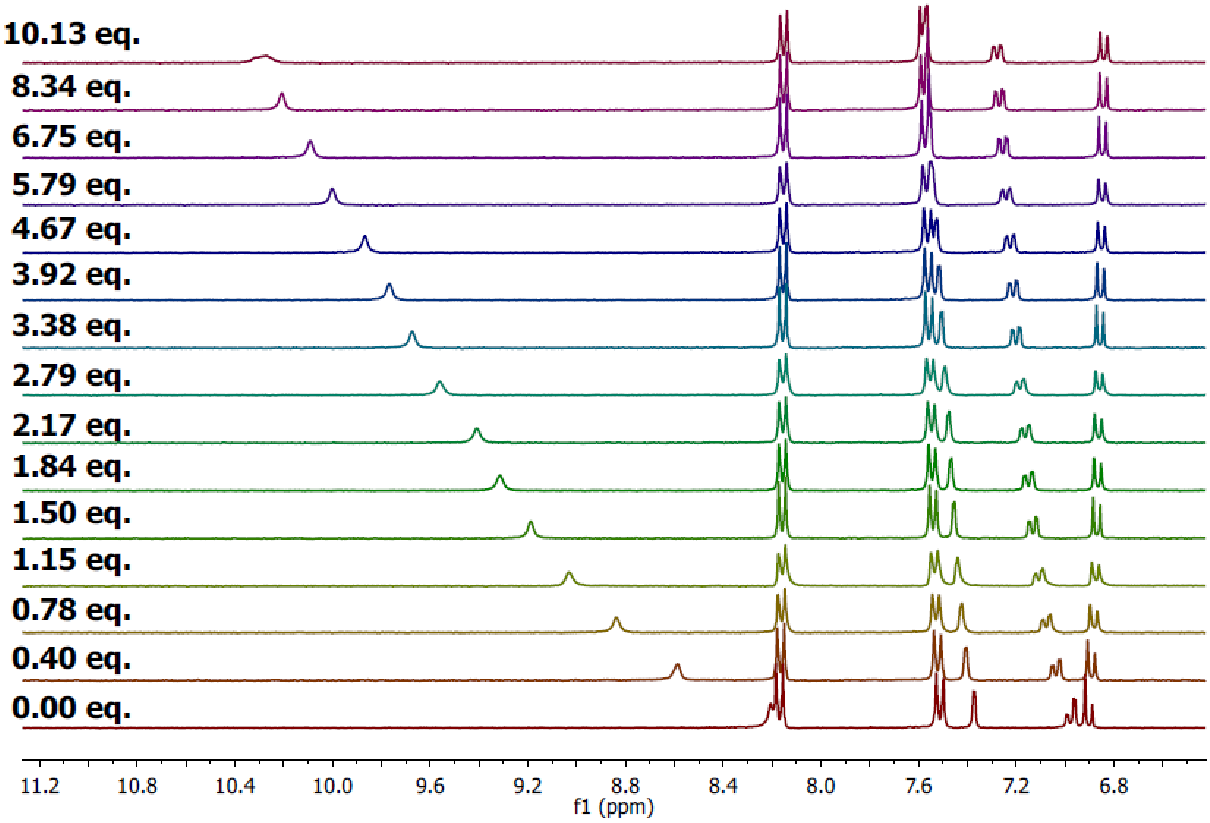

Fig. S52. ^{1}H NMR titration binding isotherms of receptor **4** ($3.11 \cdot 10^{-3}$M) in CD_3CN upon addition of increasing amounts of TBACl in the presence of KPF_6.

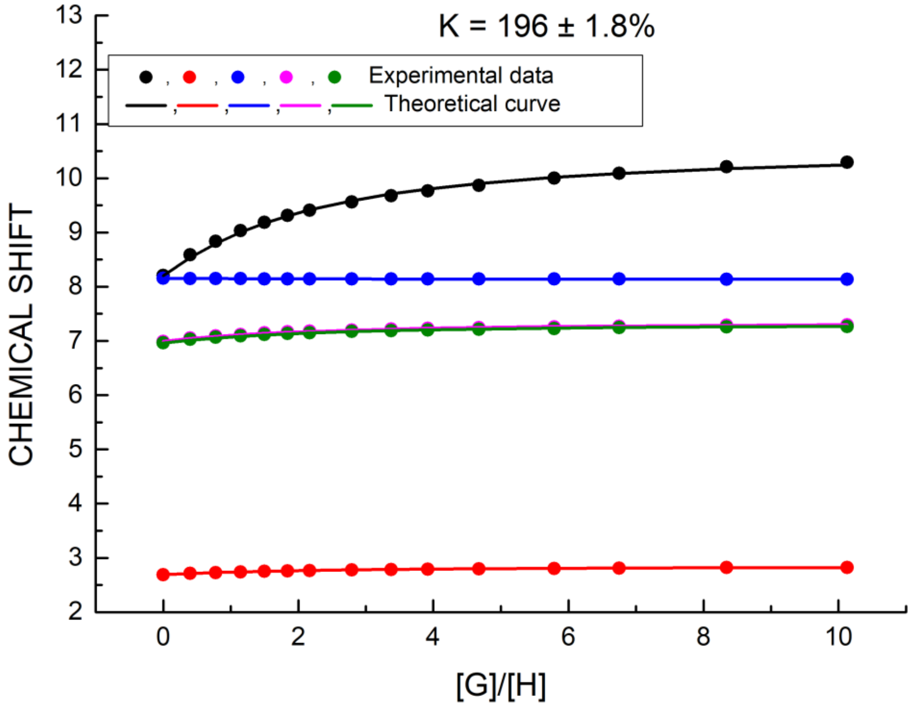

Fig. S53. ^{1}H NMR spectra recorded upon titration of receptor **2** in CD_3CN with TBACl.

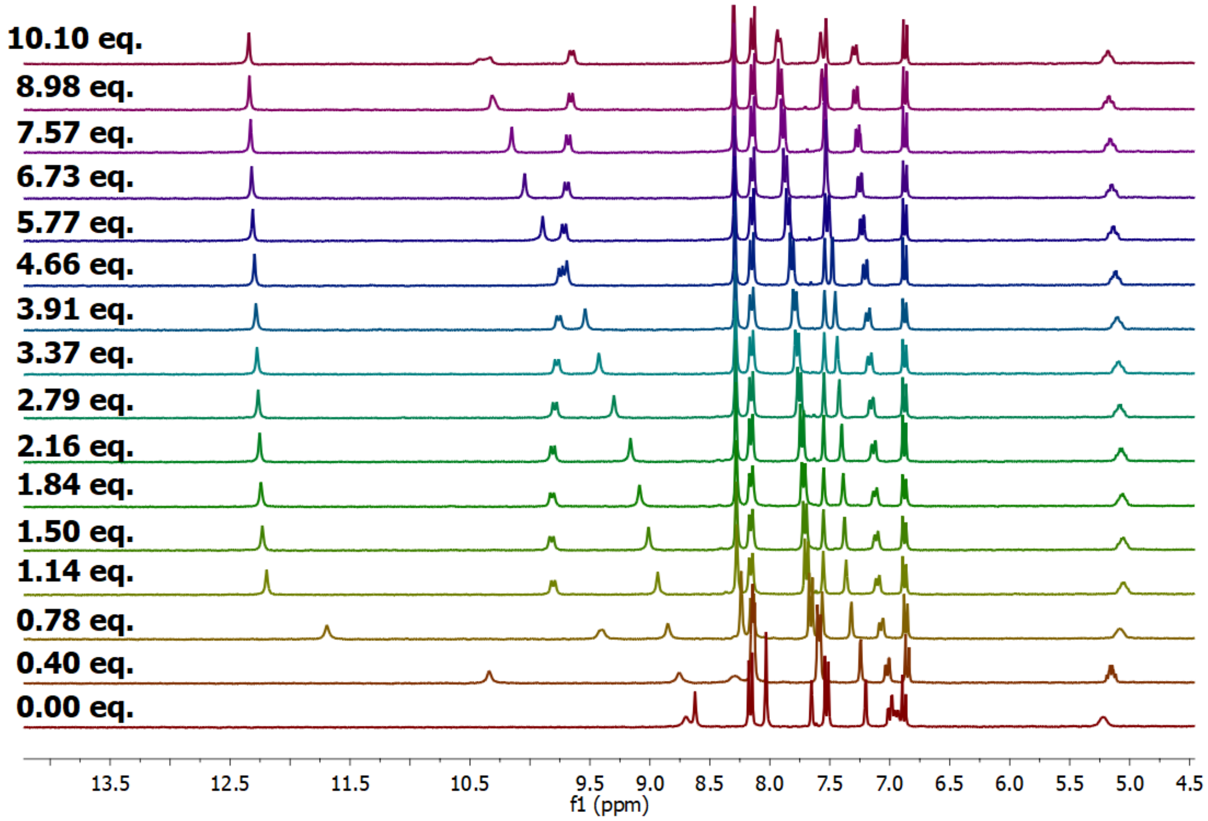

Fig. S54. ^{1}H NMR spectra recorded upon titration of receptor **2** in CD_3CN with TBACl in the presence of 1 eq. KPF_6.

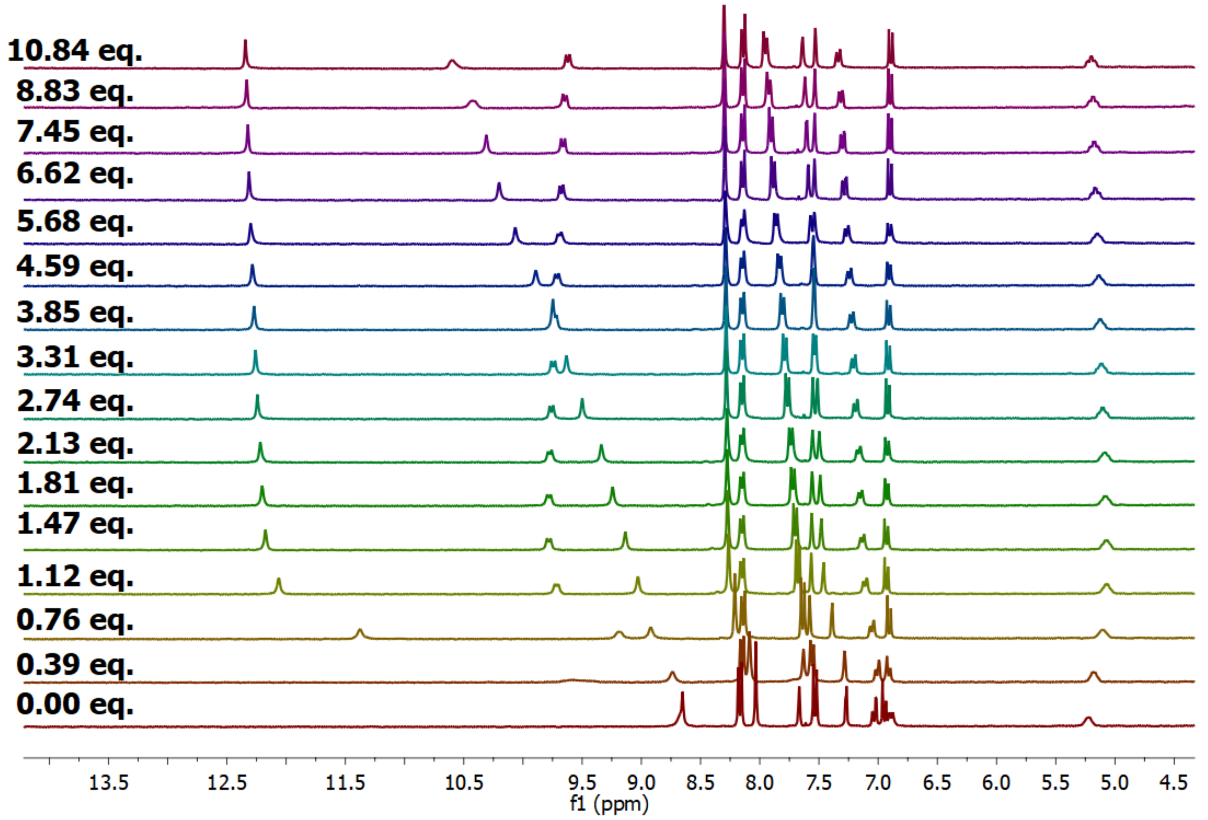

Fig. S55: ROESY spectrum of **2** with 10 equivalents of TBACl in CD_3CN.

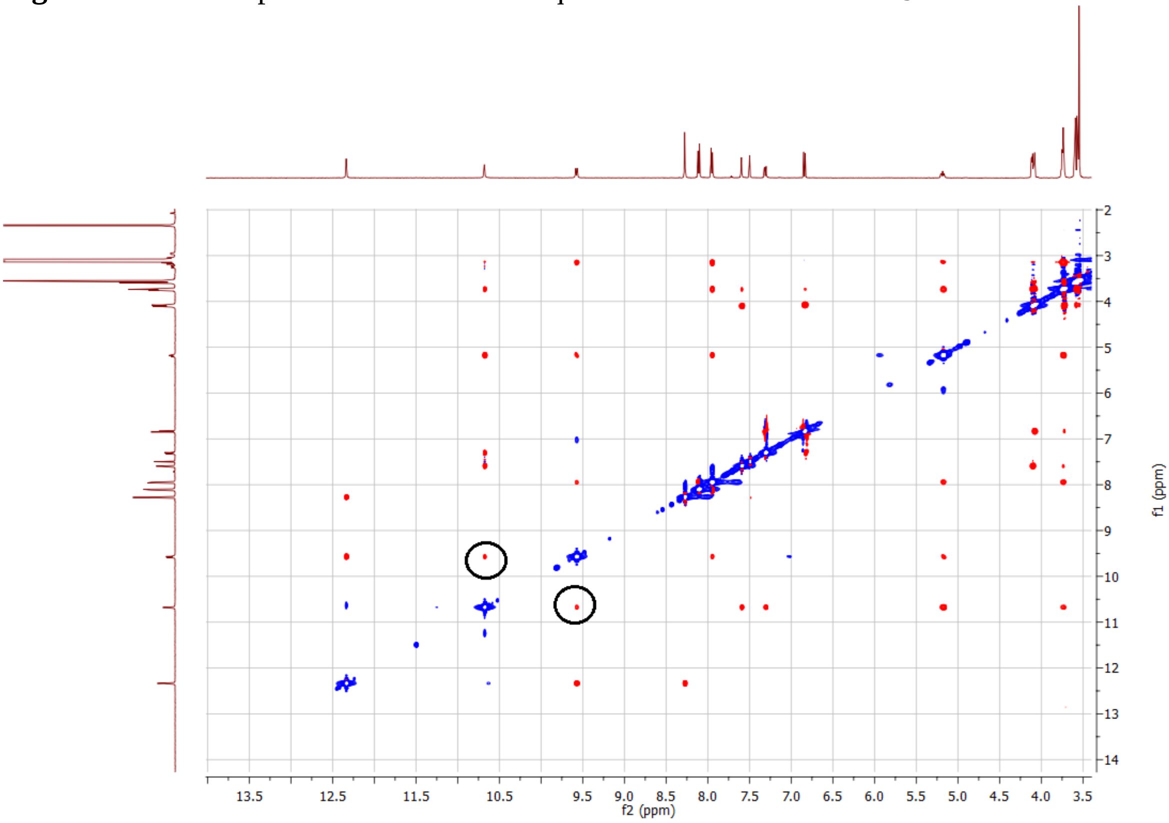

Fig. S55a: ROESY spectrum of **2** in CD_3CN (zoom).

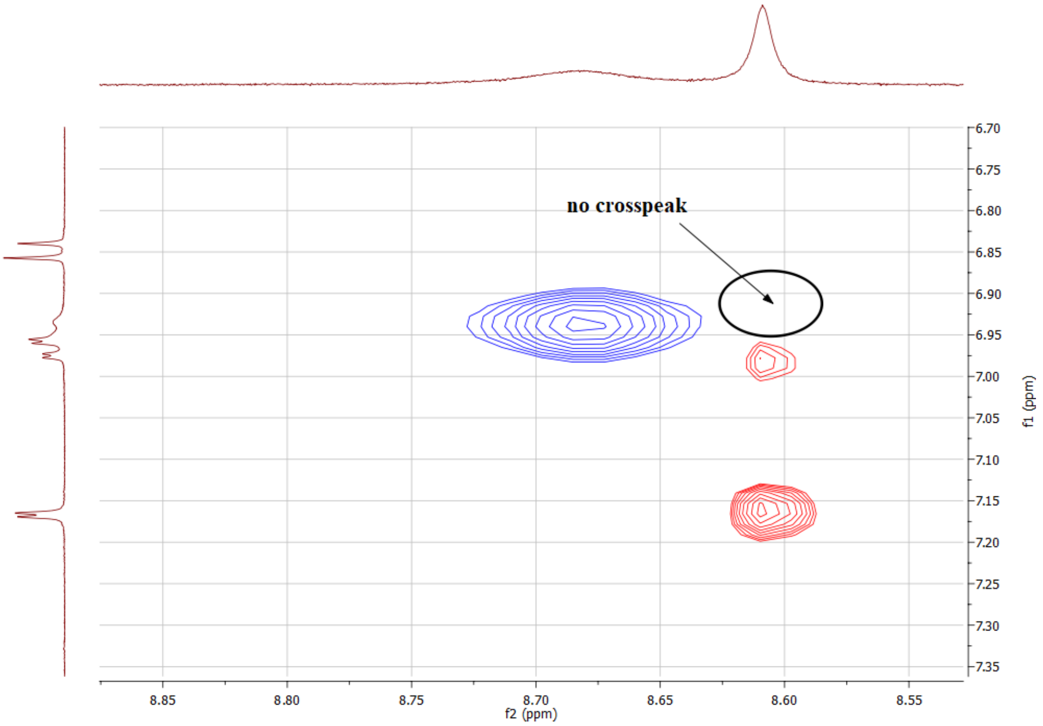

Fig. S55c: ROESY spectrum of **2** with 10 equivalents of TBACl in CD_3CN (zoom).

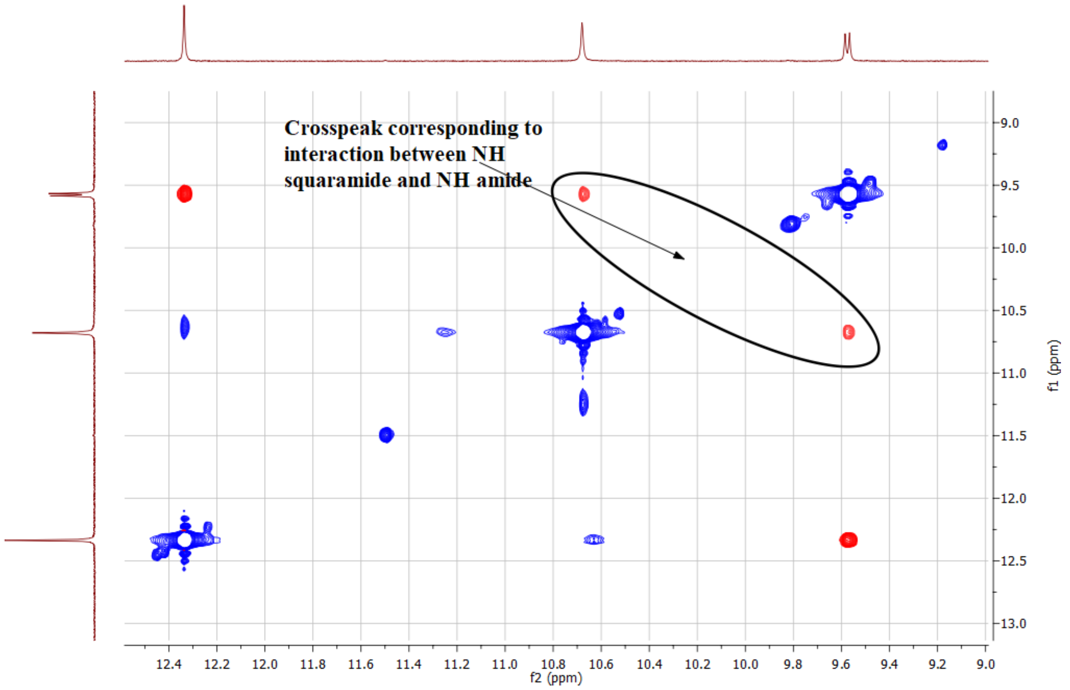

Fig. S56: COSY spectrum of **2** with 10 equivalents of TBACl in CD_3CN.

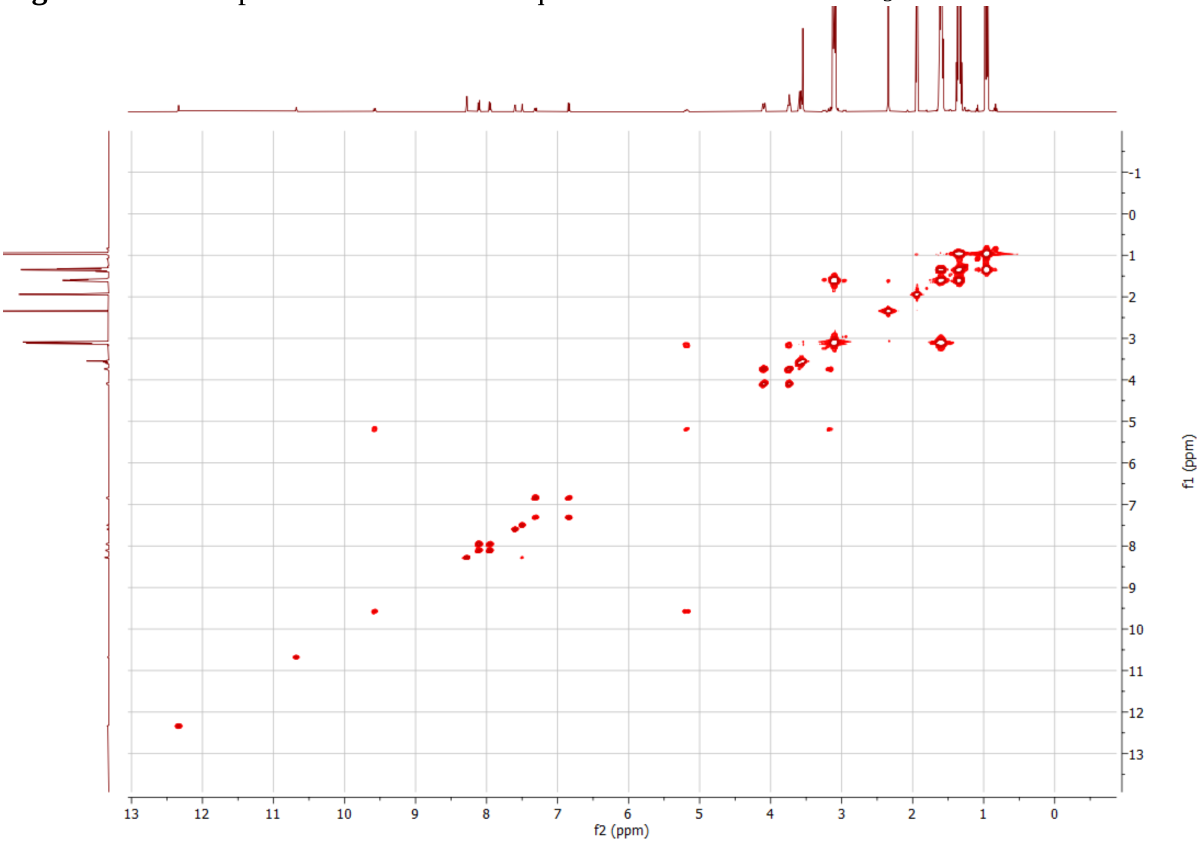

Fig. S57: HMBC spectrum of **2** with 10 equivalents of TBACl in CD_3CN.

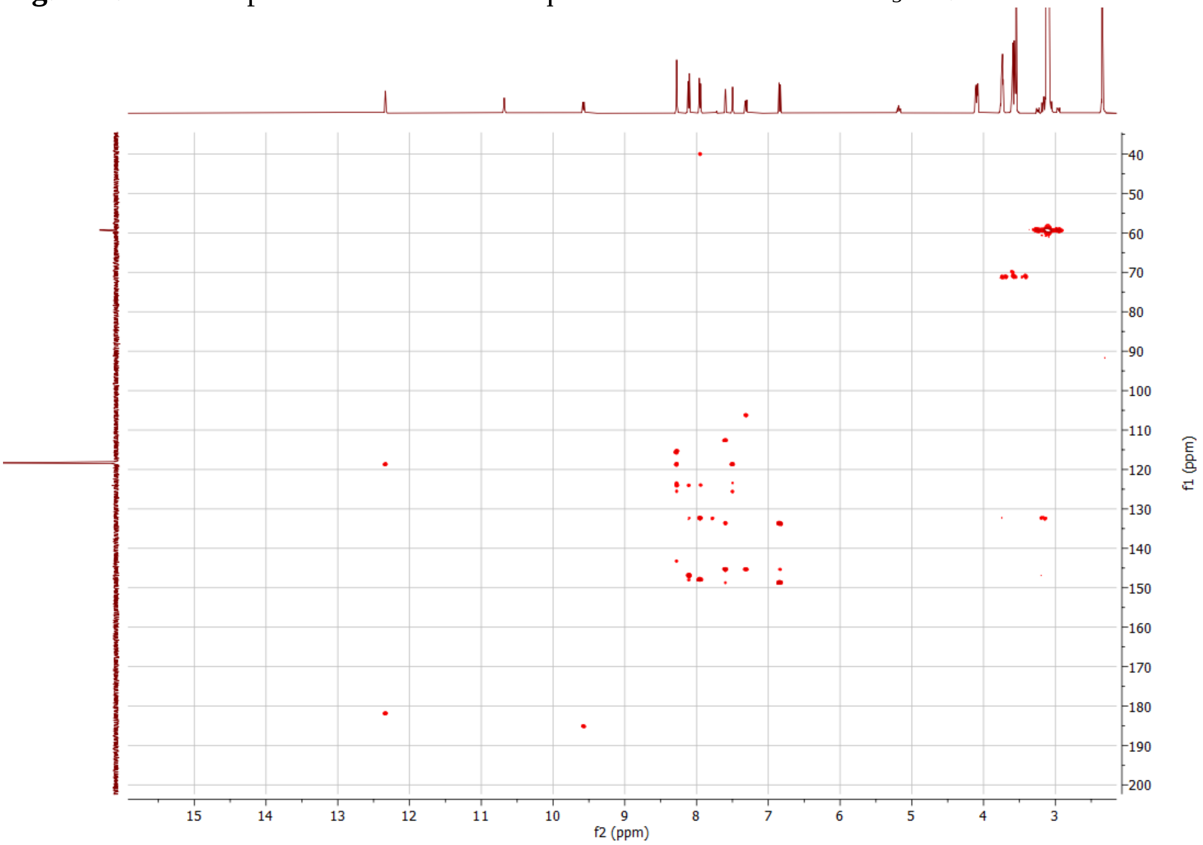

Fig. S58: HSQC spectrum of **2** with 10 equivalents of TBACl in CD_3CN.

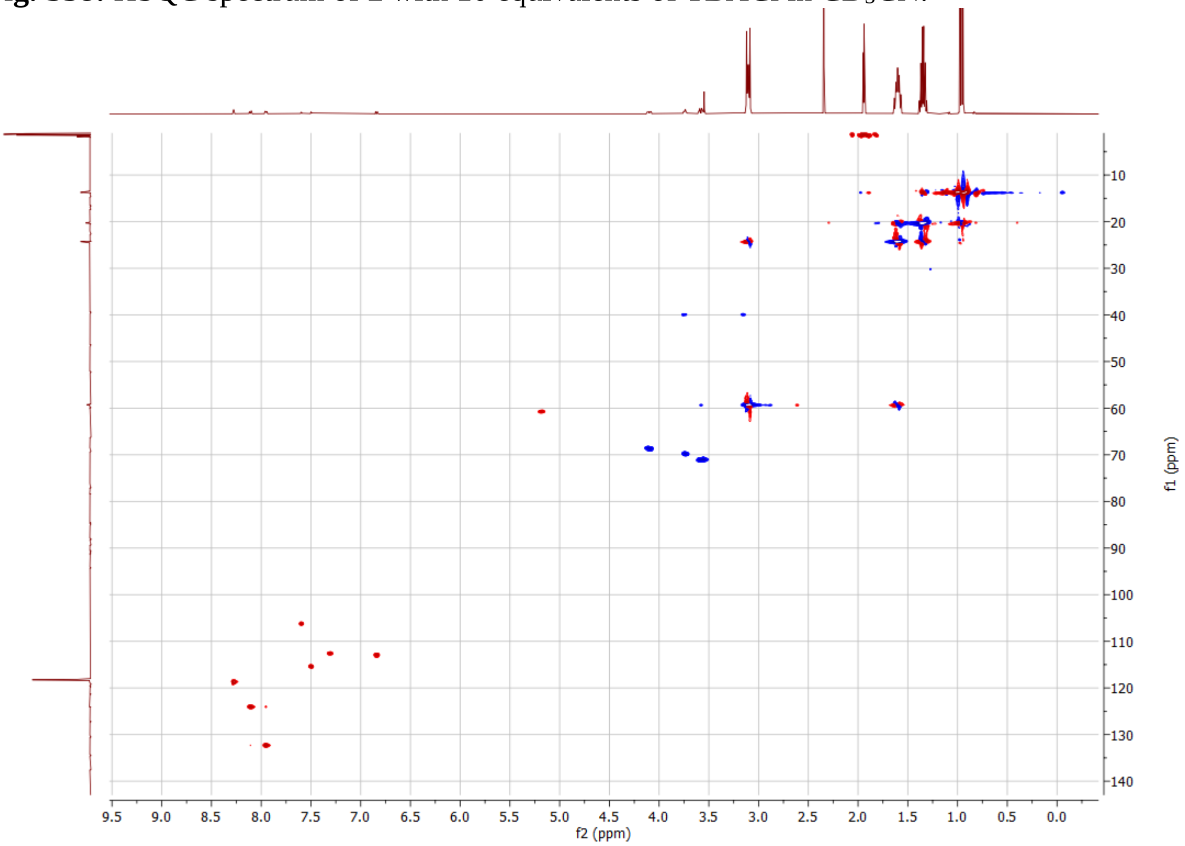

Fig. S59. ^{1}H NMR spectra recorded upon titration of receptor **2** in CD_3CN with TBA_2SO_4.

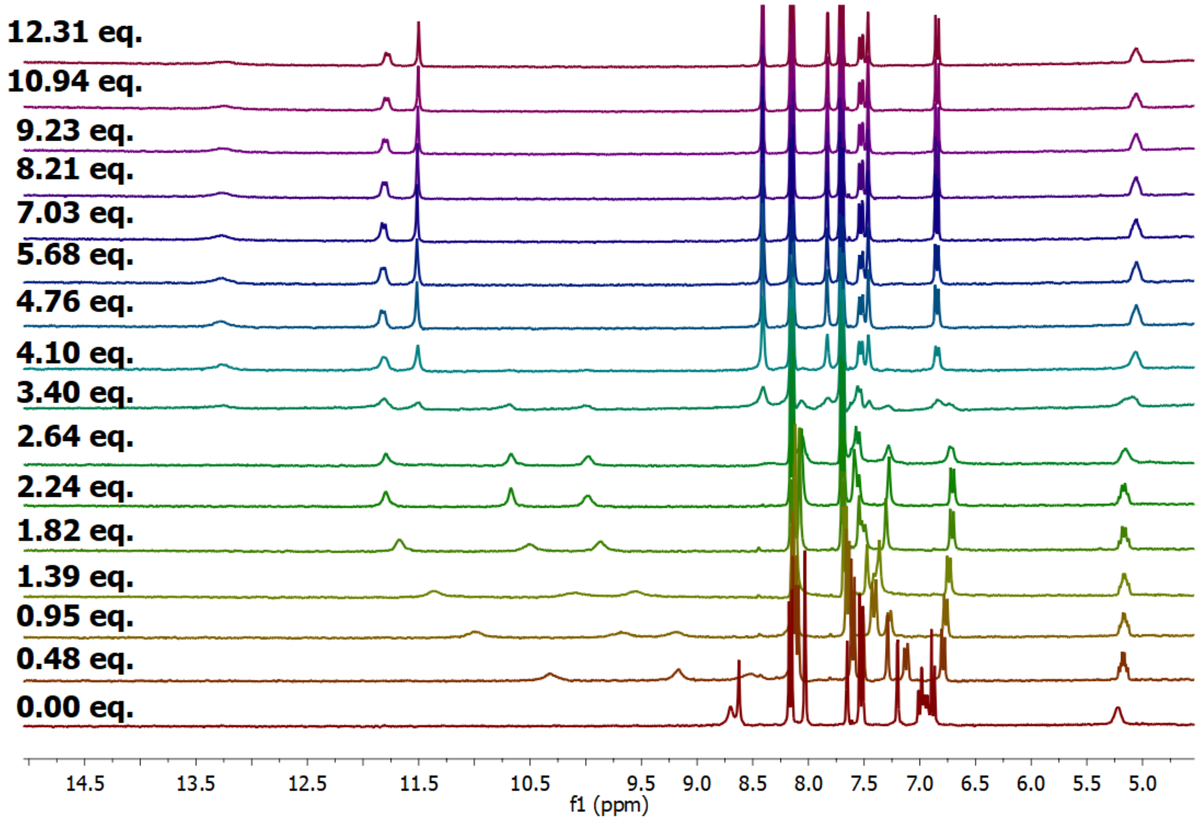

Fig. S60. ^{1}H NMR spectra recorded upon titration of receptor **2** in CD_3CN with TBA_2SO_4 in the presence of 1 eq. KPF_6.

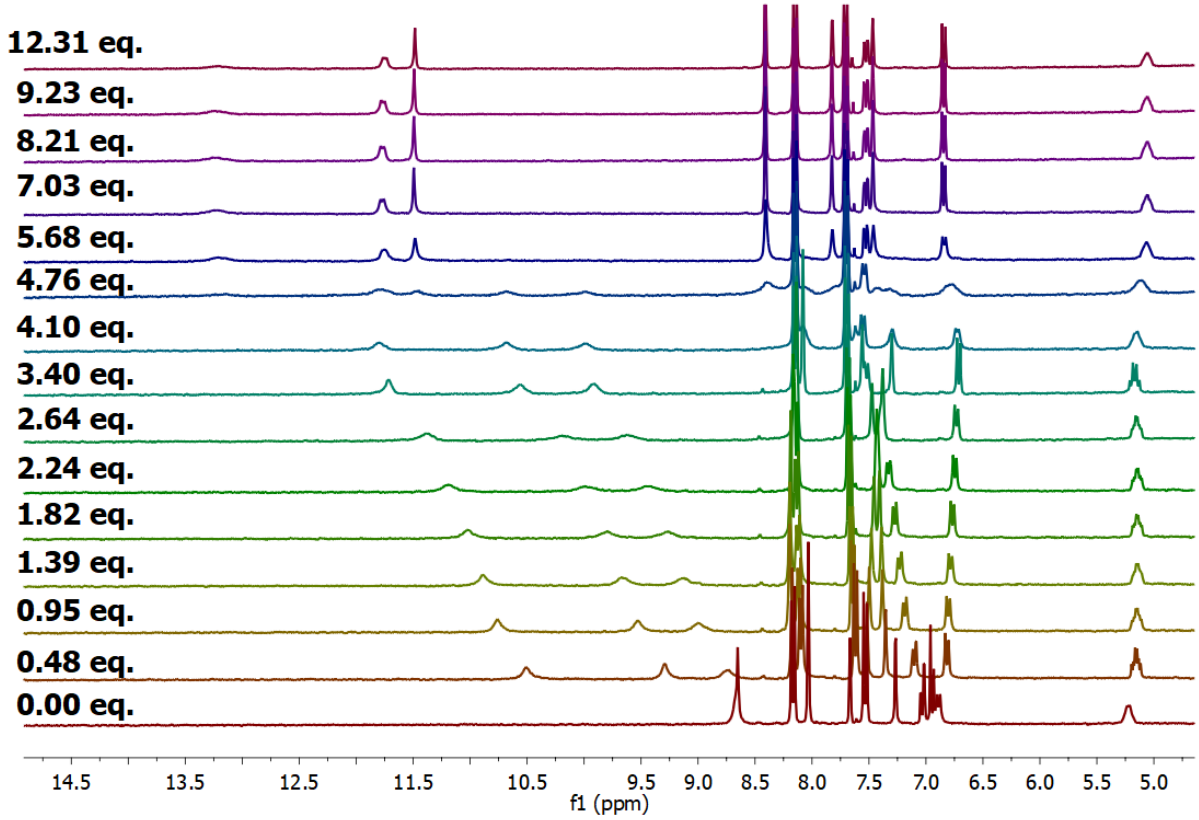

Fig. S61: ROESY spectrum of **2** with 2 equivalents of TBA_2SO_4 in CD_3CN.

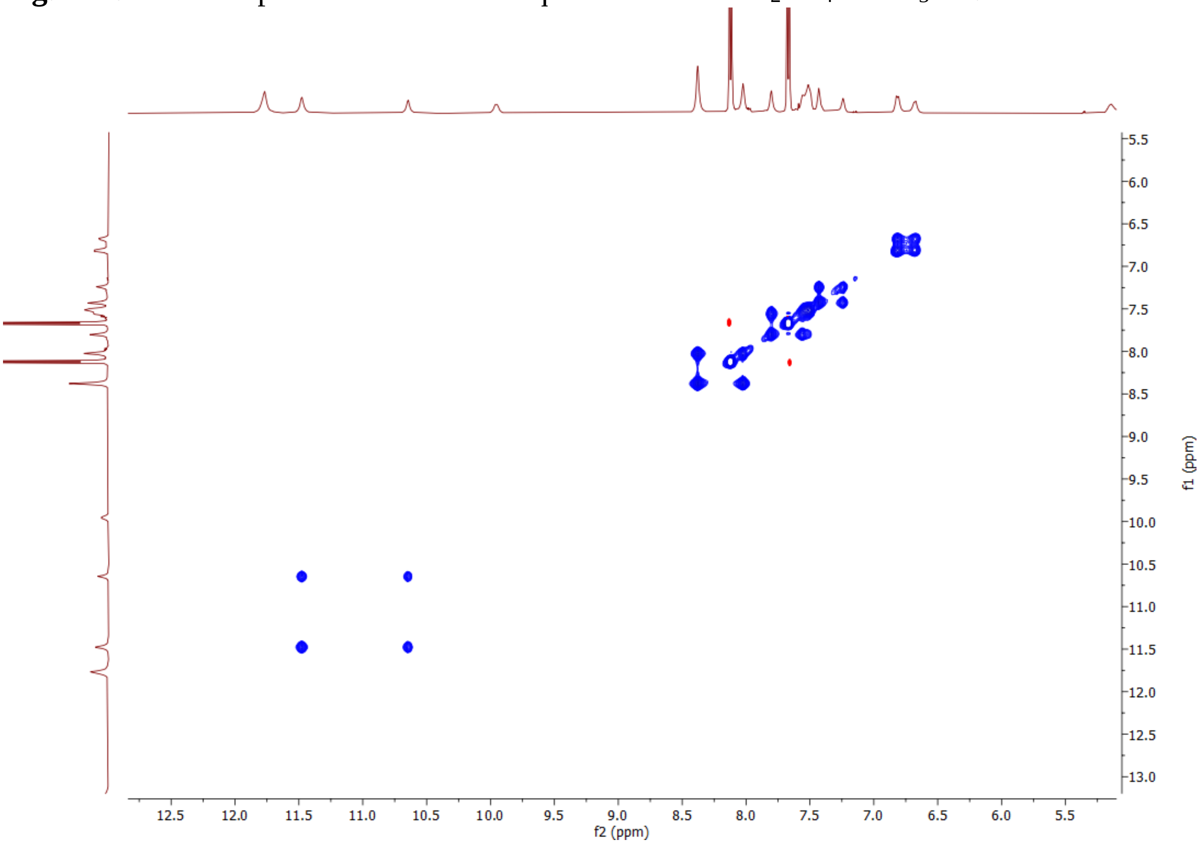

Fig. S62: COSY spectrum of **2** with 2 equivalents of TBA_2SO_4 in CD_3CN.

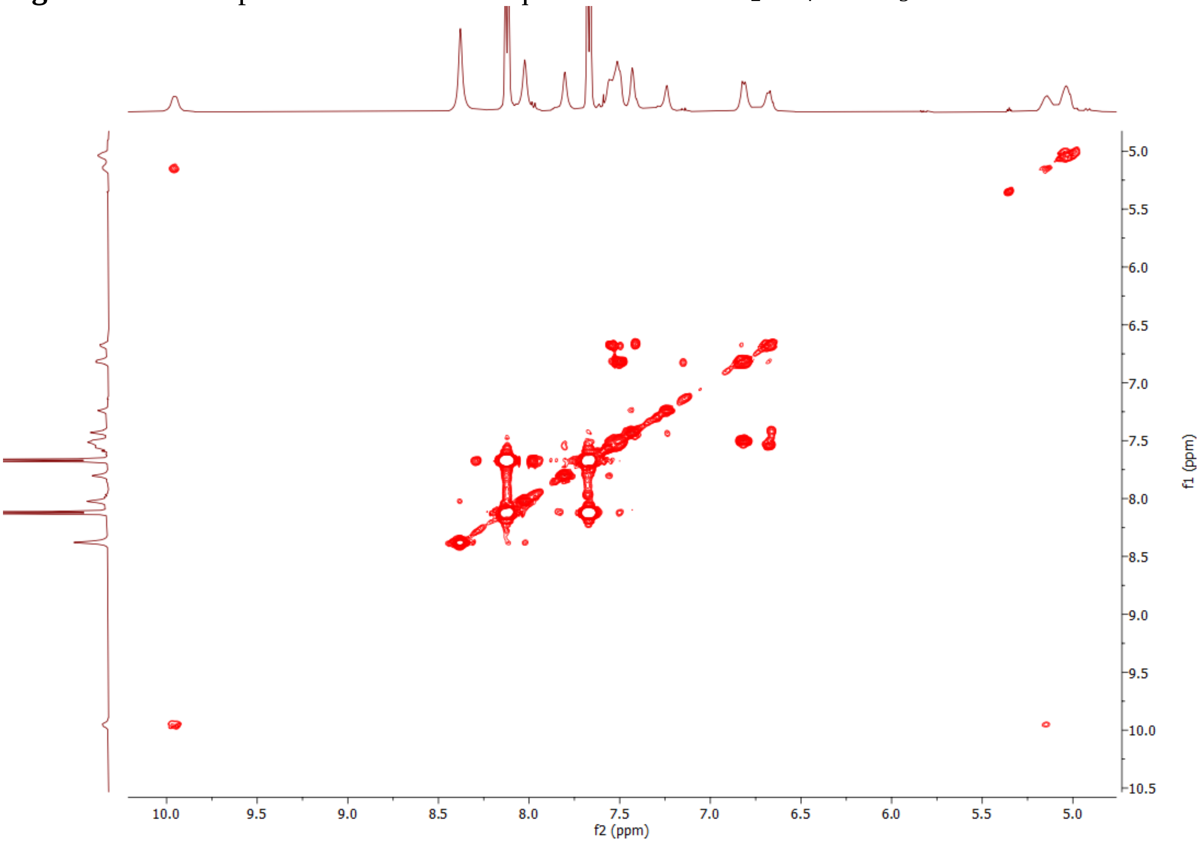

Fig. S63: HSQC spectrum of **2** with 2 equivalents of TBA_2SO_4 in CD_3CN.

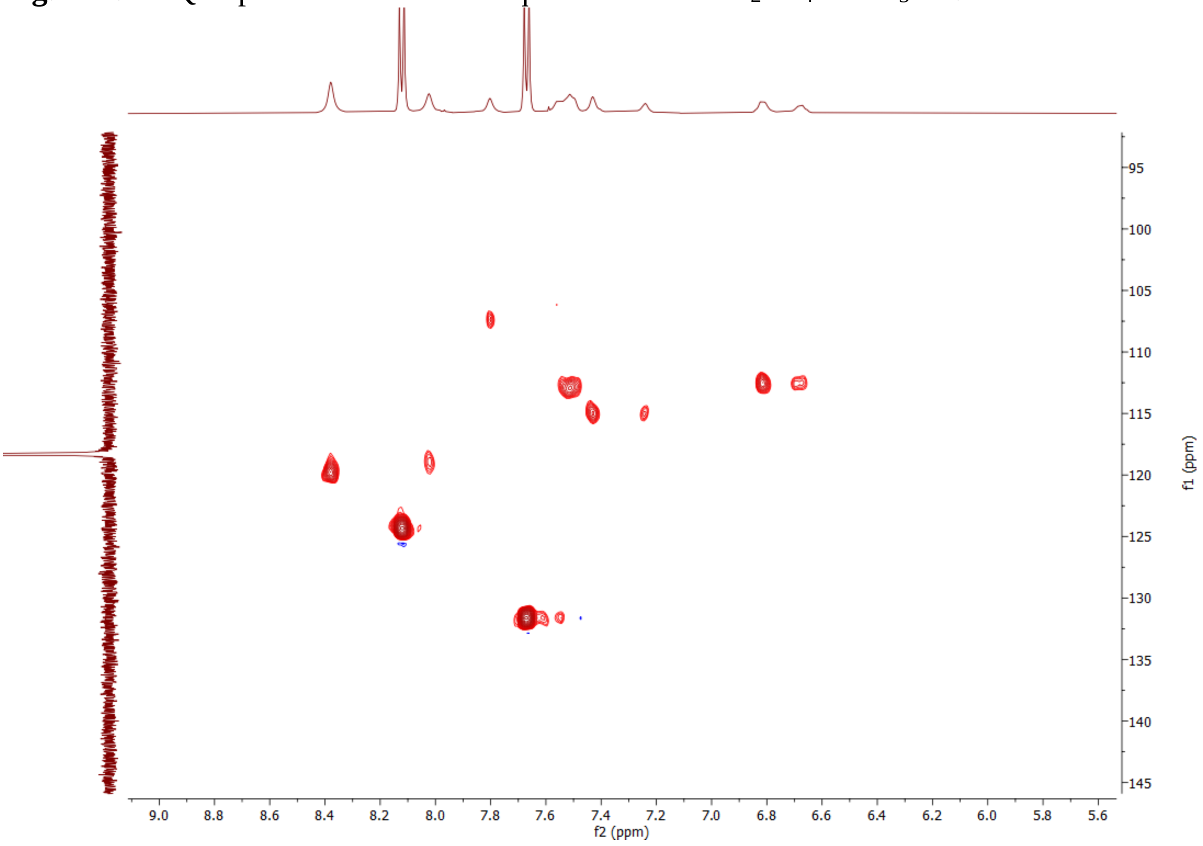

Fig. S64: HMBC spectrum of **2** with 2 equivalents of TBA_2SO_4 in CD_3CN.

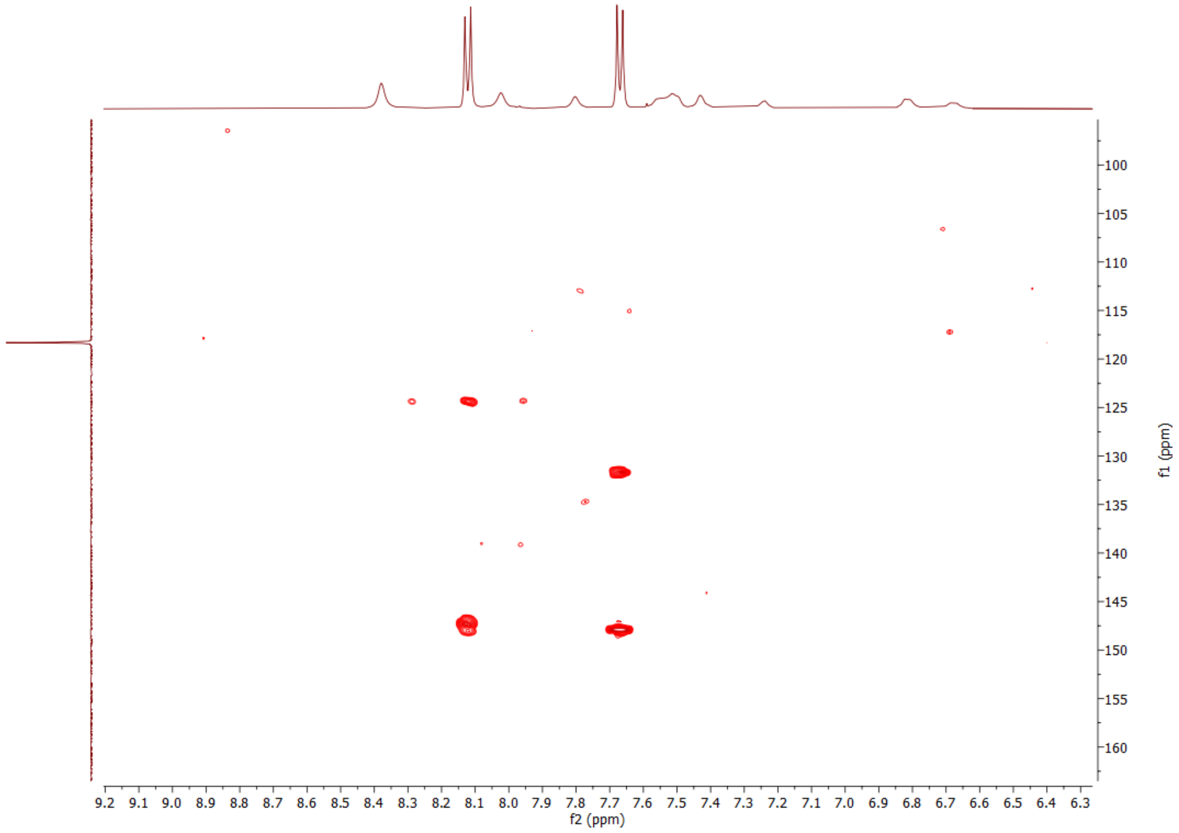

Fig. S65: TOCSY spectrum of **2** with 2 equivalents of TBA_2SO_4 in CD_3CN

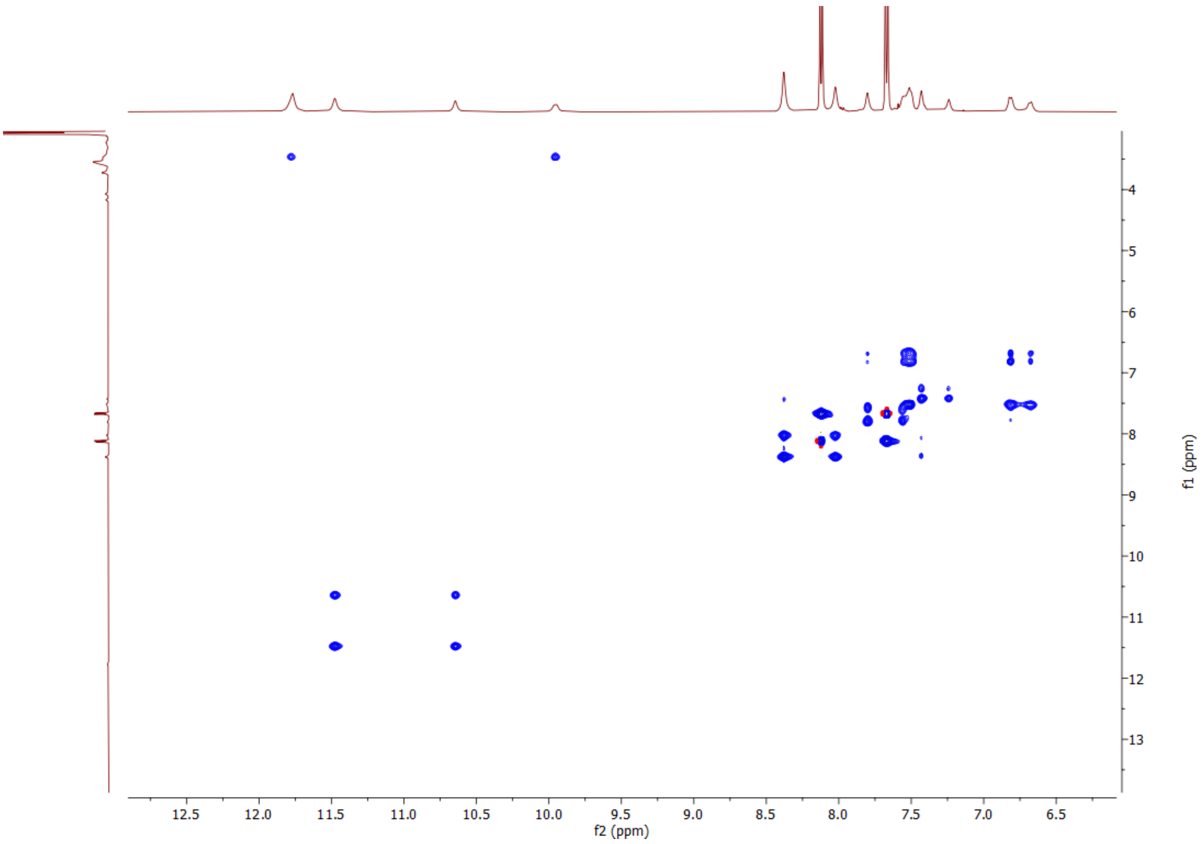

Fig. S66: COSY spectrum of **2** with 5 equivalents of TBA_2SO_4 in CD_3CN

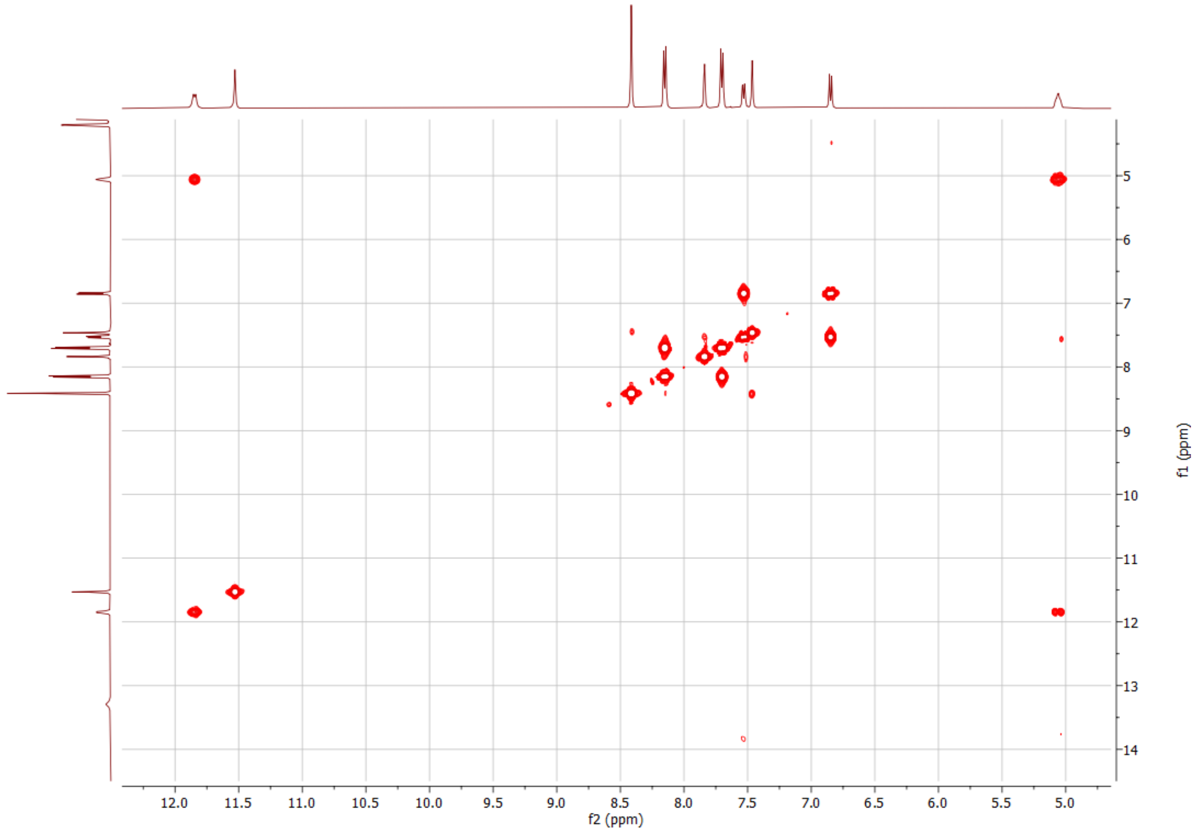

Fig. S67: HSQC spectrum of **2** with 5 equivalents of TBA_2SO_4 in CD_3CN.

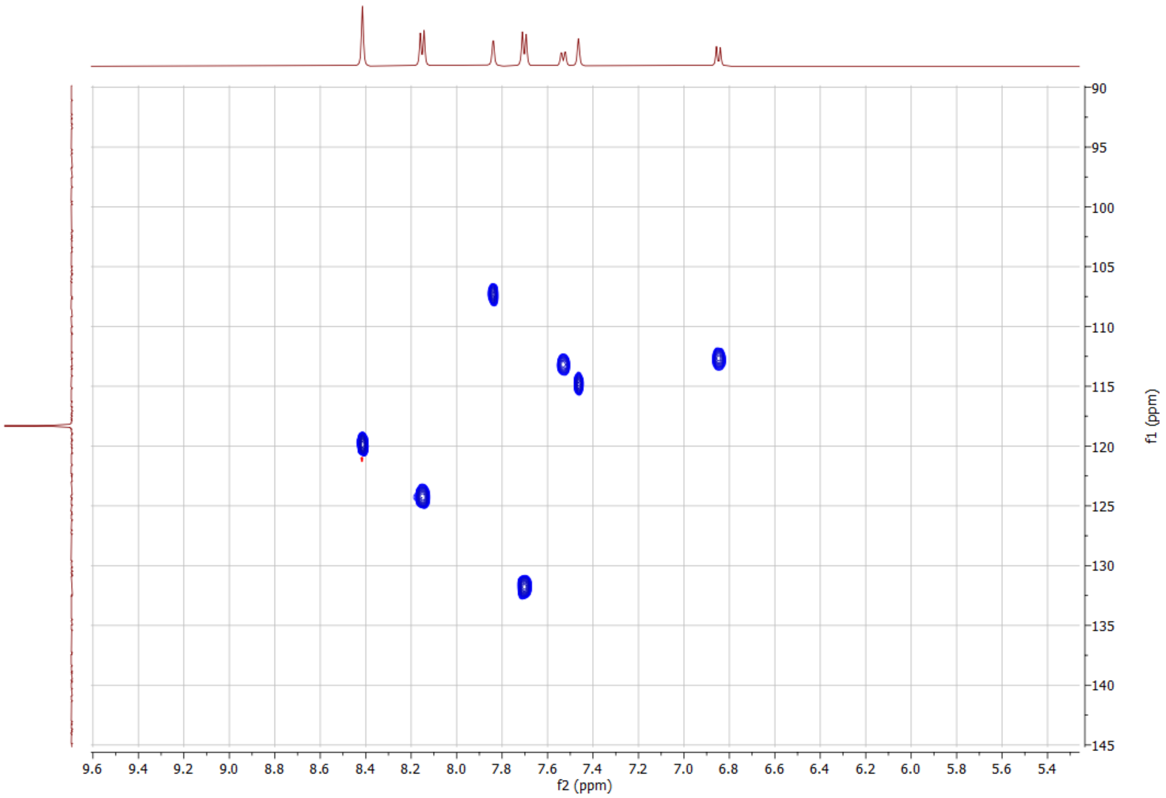

Fig. S68: HMBC spectrum of **2** with 5 equivalents of TBA_2SO_4 in CD_3CN.

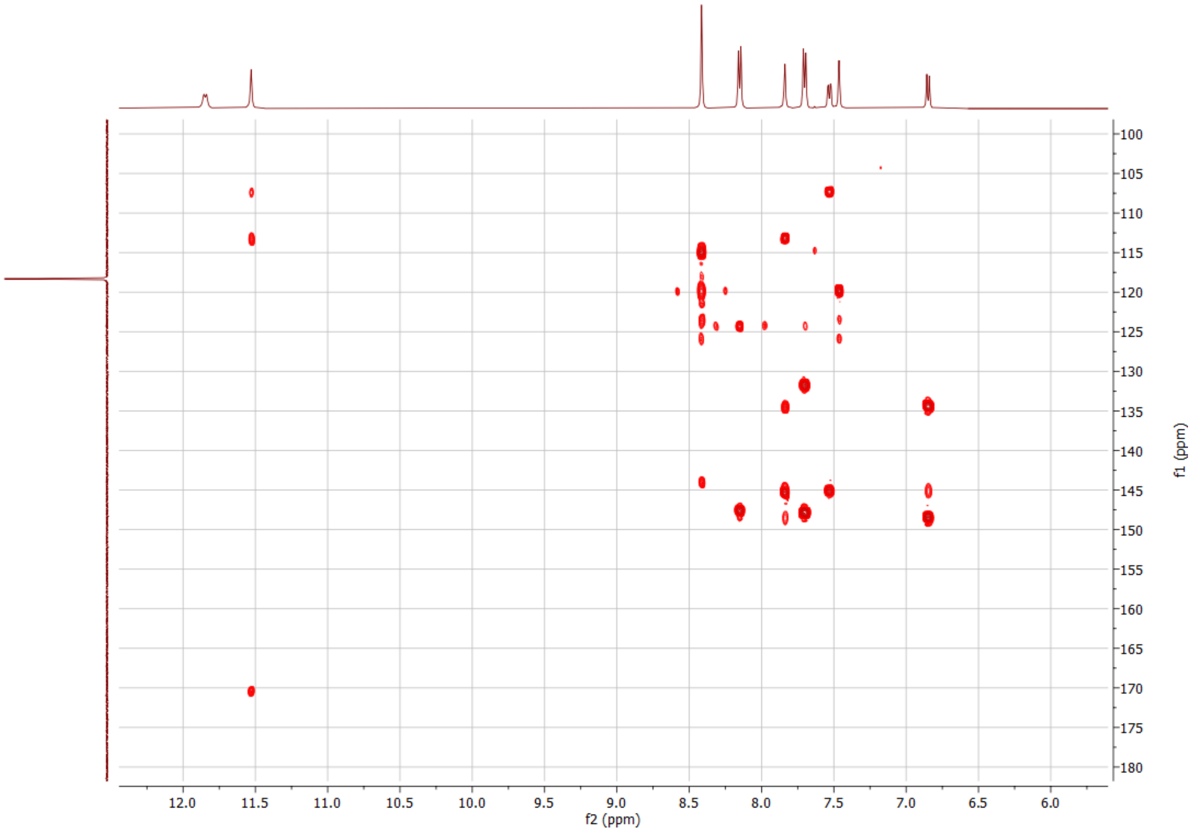

Fig. S69: ROESY spectrum of **2** with 5 equivalents of TBA_2SO_4 in CD_3CN.

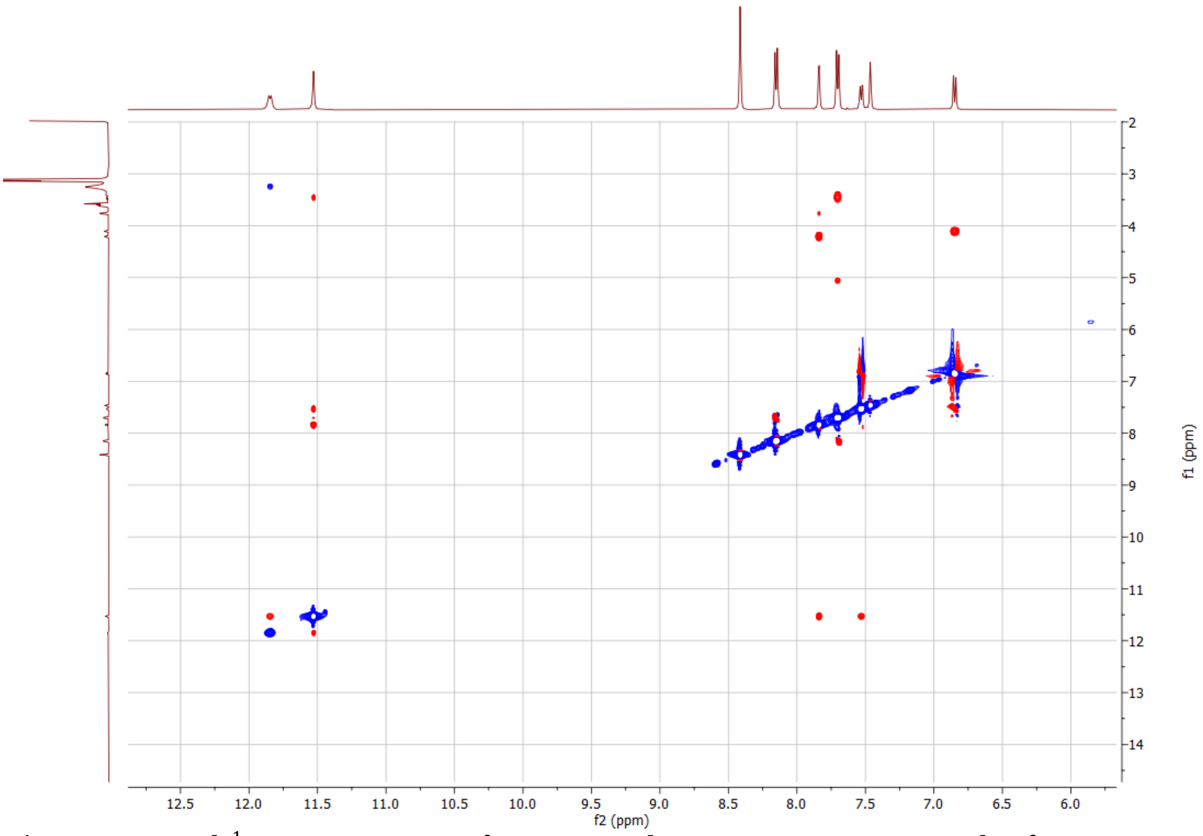

Fig. S70. Partial ^1H NMR spectra of a 3 mM solution receptor **2** in $CDCl_3$ after contacting with : (a) water, and aqueous 0.5 M solutions of (b) NaCl, (c) NaBr, (d) $NaNO_3$, (e) $NaNO_2$, (f) Na_2SO_4, (g) NaH_2PO_4.

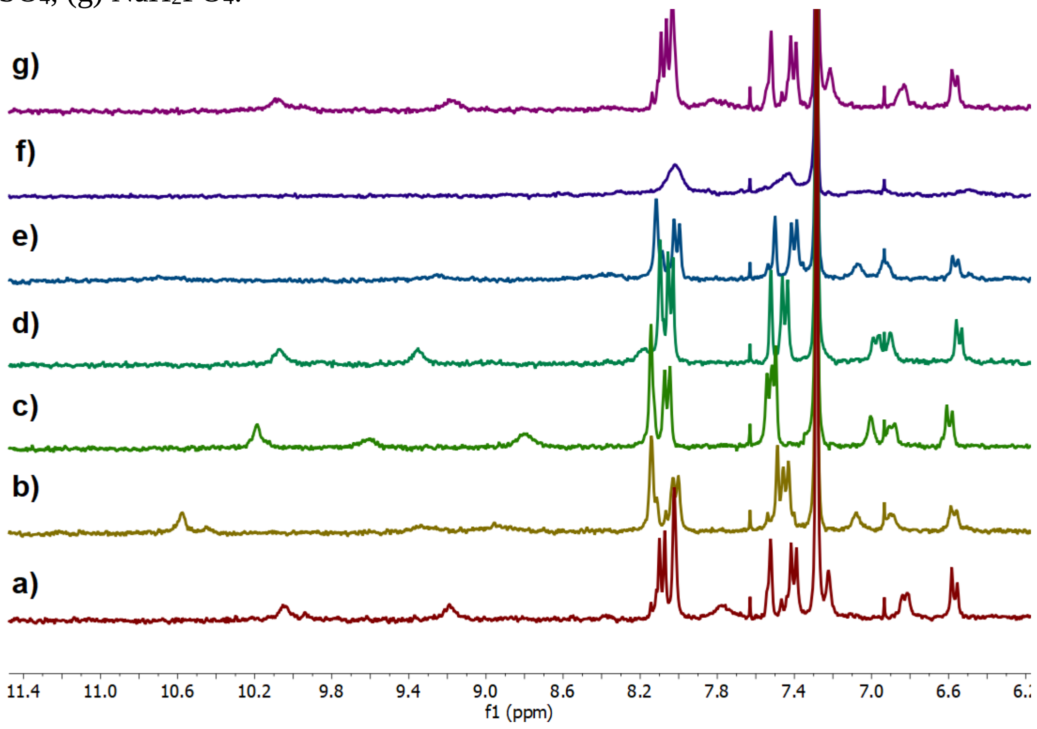

EXTRACTION EXPERIMENTS - ION CHROMATOGRAPHY

A solution of receptors **1**, **2** in chloroform (2 ml, ca. 5 mM or 20 mM) was intensive shaking with 5 mM aqueous solutions of suitable salts (2 ml) or mixture of salts for 5 minutes. Then 1 mL of aqueous phase was taken and tenfold diluted. The concentration of chloride, bromide, nitrite, nitrate, phosphate and sulfate anions in aqueous phase was determined by high performance ion chromatography (HPIC).

Fig. S71. Chromatograms obtained during extraction experiments after tenfold dilution (a) 5 Mm source phase aqueous solution of sodium salts of: NaCl, NaBr, $NaNO_2$, $NaNO_3$, NaH_2PO_4, Na_2SO_4 solution (b) after extraction with 5 mM of **1** in $CHCl_3$.

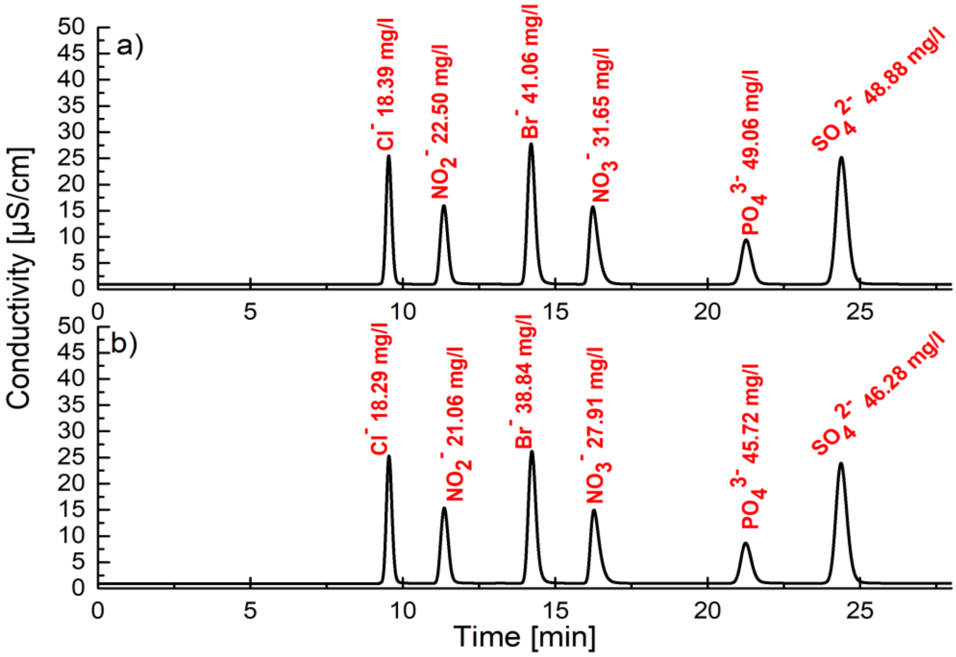

Fig. S72. Chromatograms obtained during extraction experiments after tenfold dilution (a) 5 Mm source phase aqueous solution of sodium salts of: NaCl, NaBr, $NaNO_2$, $NaNO_3$, NaH_2PO_4, Na_2SO_4 solution (b) after extraction with 5 mM of **2** in $CHCl_3$.

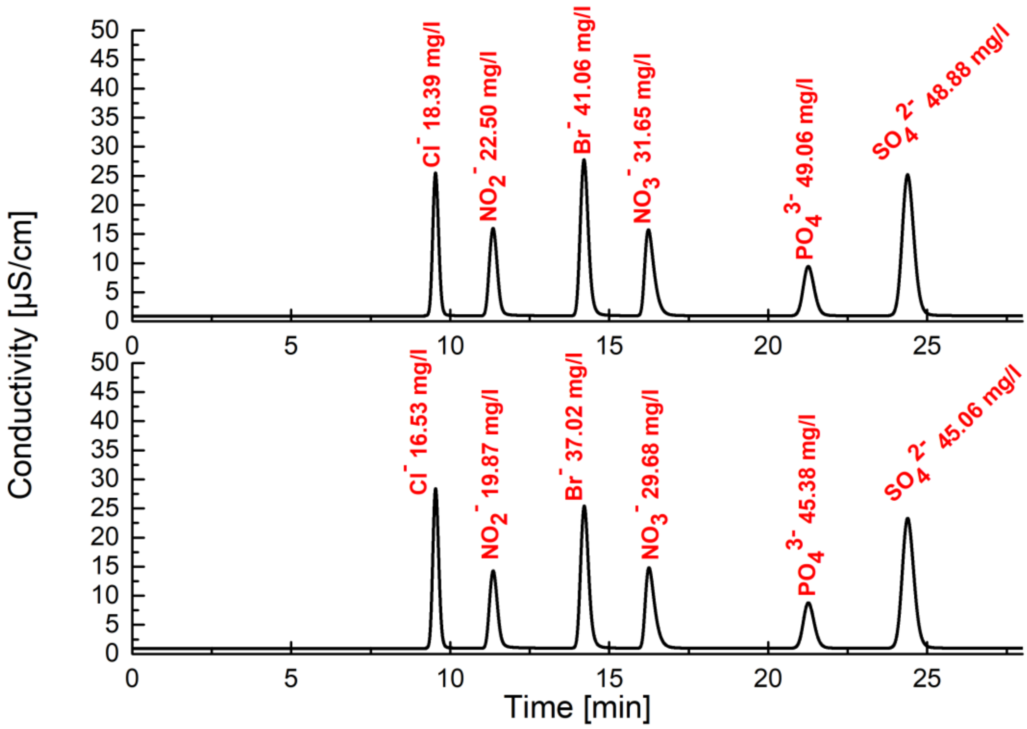

Fig. S73. Chromatograms obtained during extraction experiments after tenfold dilution (a) source phase: 5 Mm KCl solution (b) after extraction with 5 mM of **2** in $CHCl_3$.

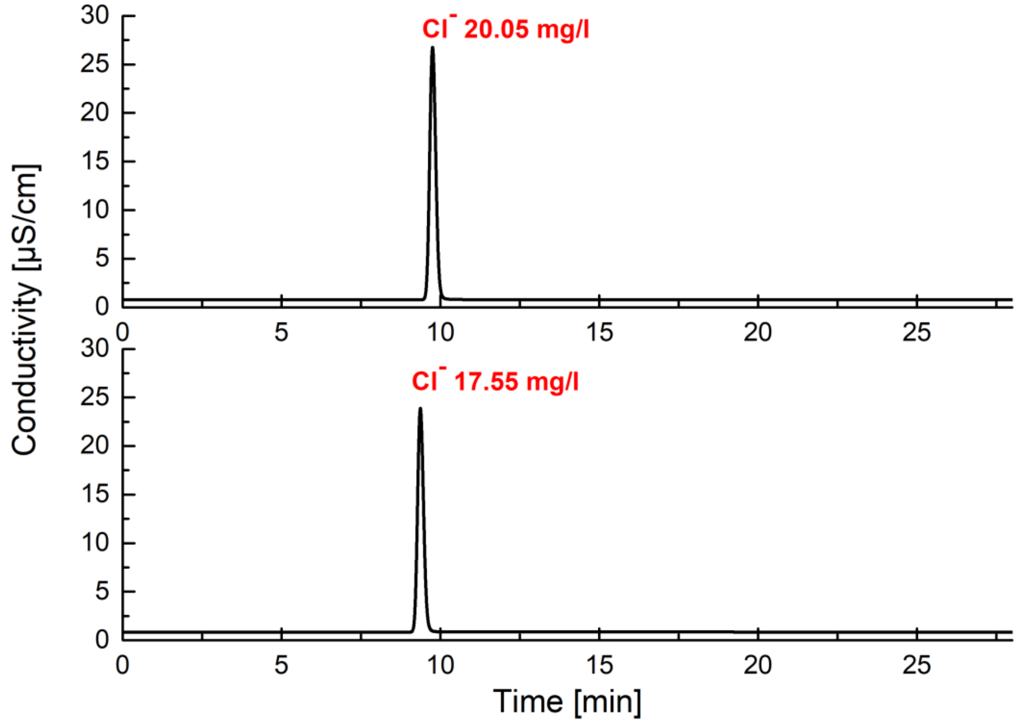

Fig. S74. Chromatograms obtained during extraction experiments after tenfold dilution (a) source phase: 5 Mm KBr solution (b) after extraction with 5 mM of **2** in $CHCl_3$.

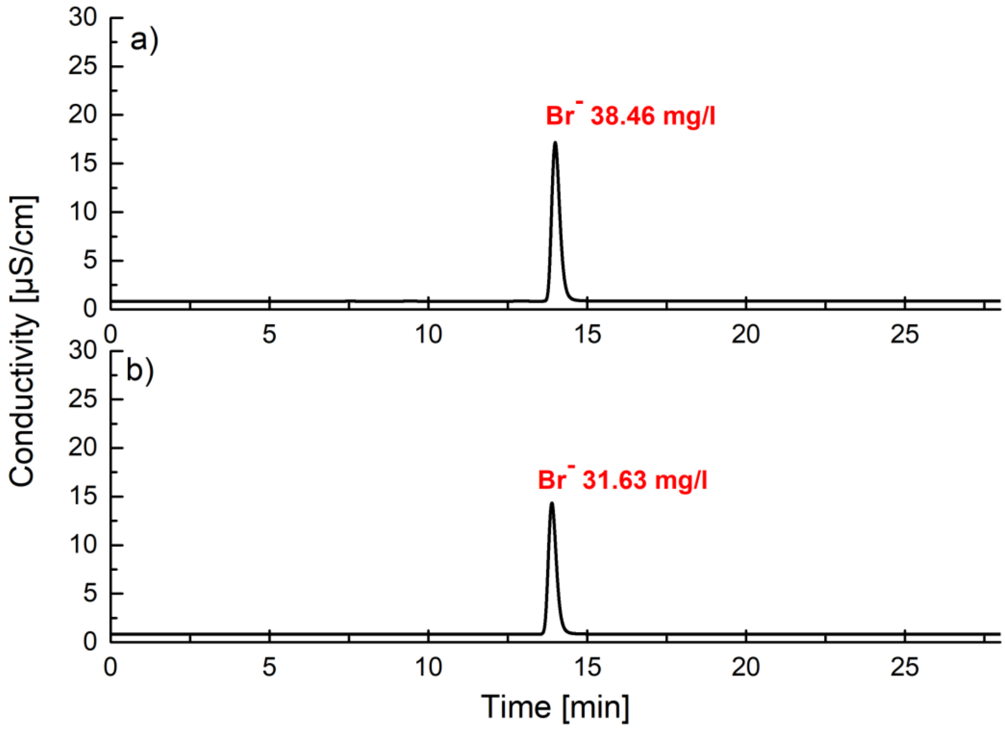

Fig. S75. Chromatograms obtained during extraction experiments after tenfold dilution (a) source phase: 5 Mm KNO_2 solution (b) after extraction with 5 mM of **2** in $CHCl_3$.

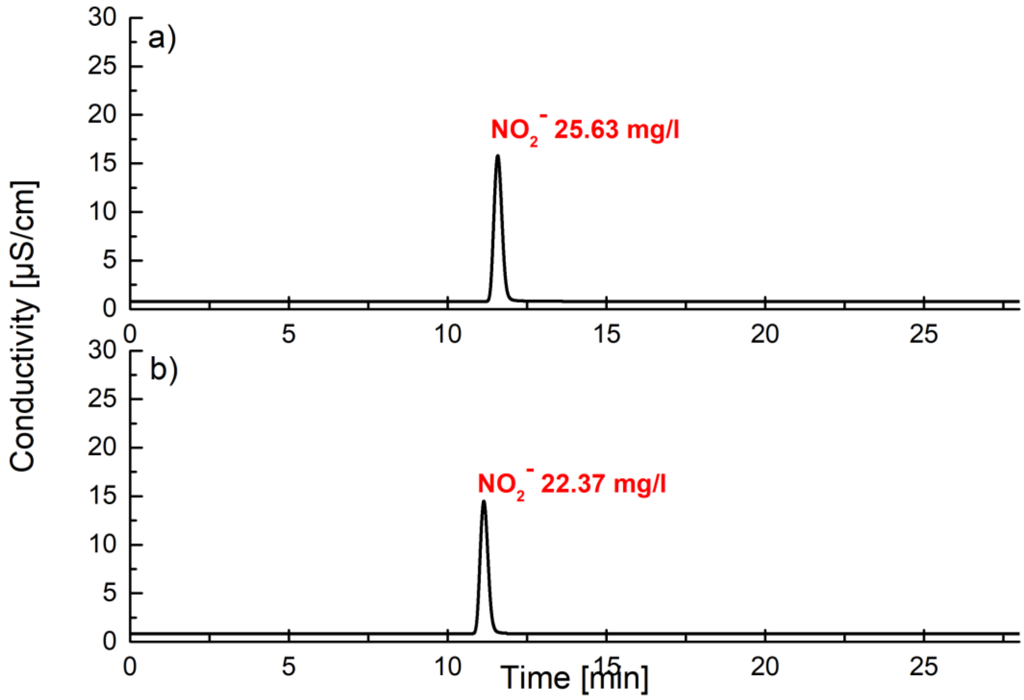

Fig. S76. Chromatograms obtained during extraction experiments after tenfold dilution (a) source phase: 5 Mm KNO_3 solution (b) after extraction with 5 mM of **2** in $CHCl_3$.

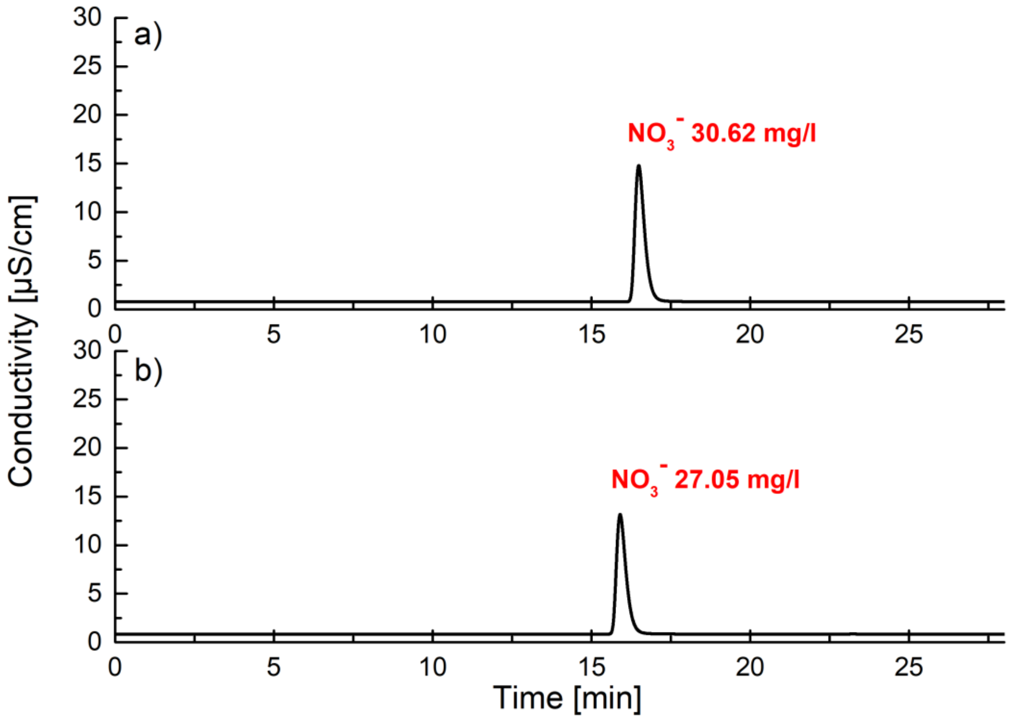

Fig. S77. Chromatograms obtained during extraction experiments after tenfold dilution (a) source phase: 5 Mm KH_2PO_4 solution (b) after extraction with 5 mM of **2** in $CHCl_3$.

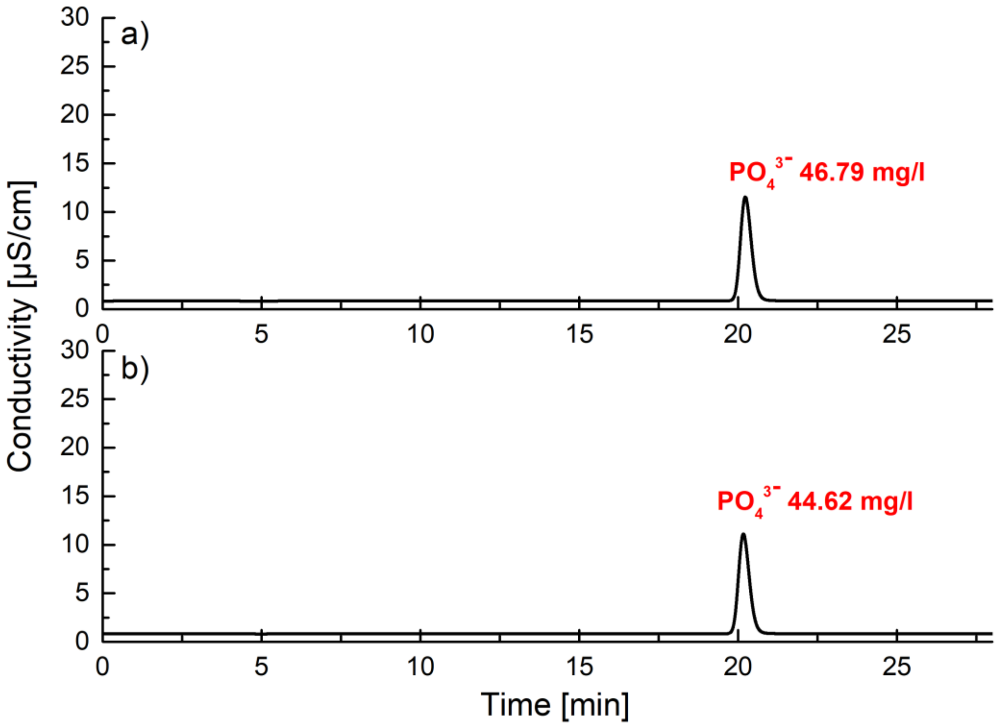

Fig. S78. Chromatograms obtained during extraction experiments after tenfold dilution (a) source phase: 5 Mm K_2SO_4 solution (b) after extraction with 5 mM of **2** in $CHCl_3$.

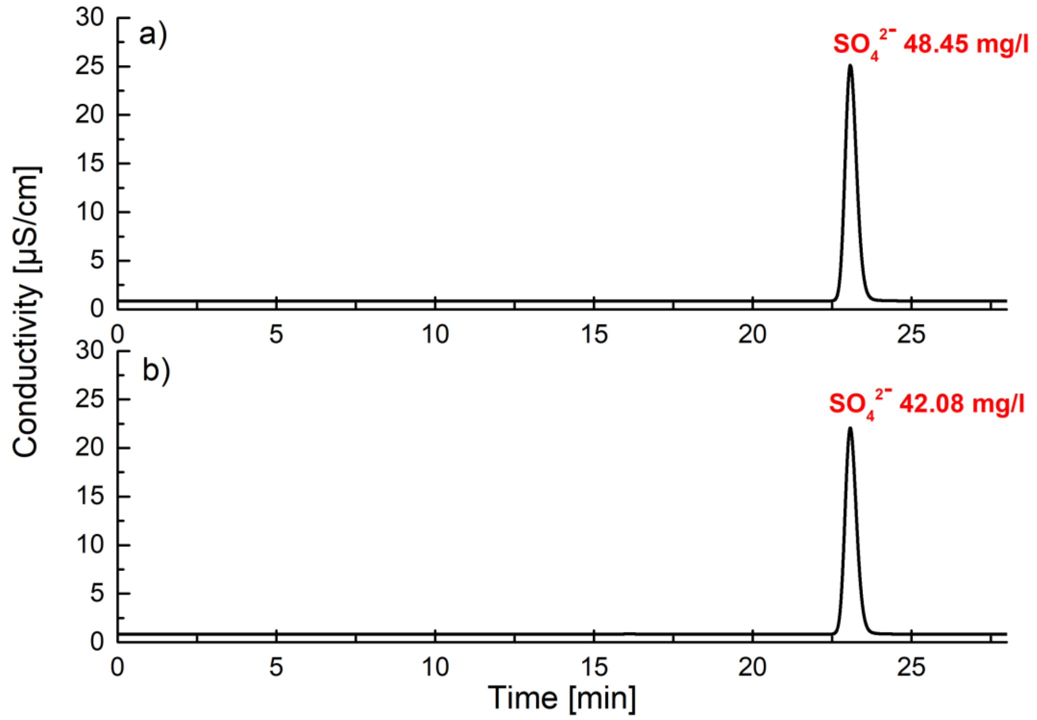

Fig. S79. Chromatograms obtained during extraction experiments after tenfold dilution (a) source phase: 5 Mm K_2SO_4 solution (b) after extraction with 20 mM of **2** in $CHCl_3$.

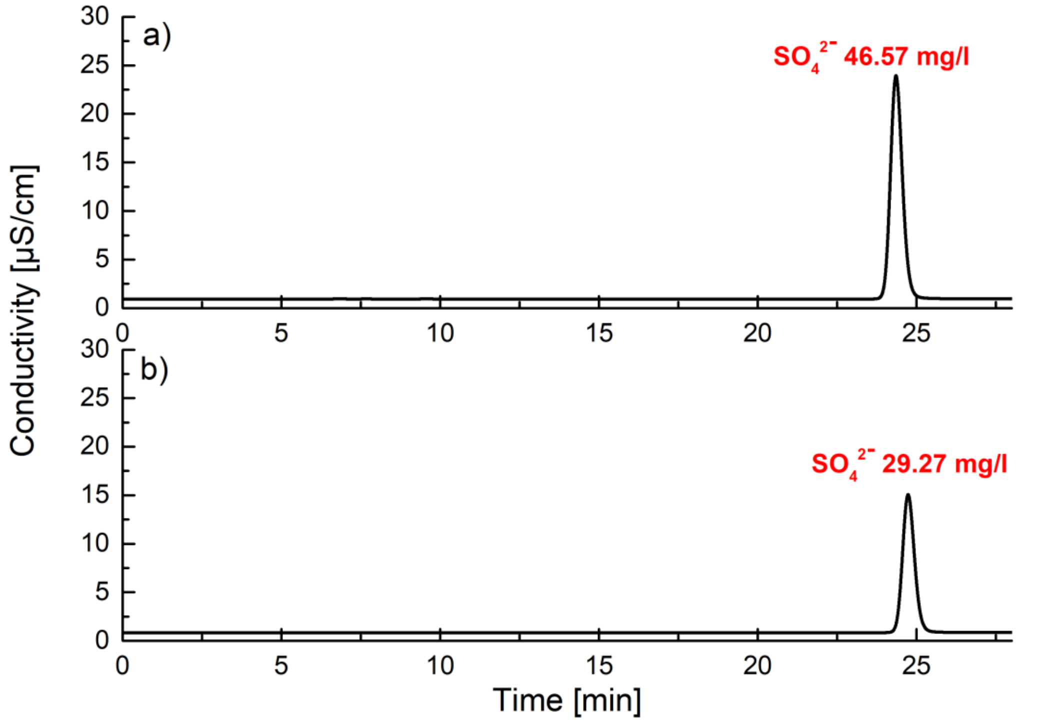

TRANSPORT ACROSS MEMBRANE

Fig. S80. Sulfates transport by **2** across a bulk chloroform membrane determined by the chloride concertation in the receiving phase. Source phase: 50 mM solution of TBA_2SO_4, K_2SO_4 in water; organic phase: 5 mM **2** in $CHCl_3$; receiving phase: water.

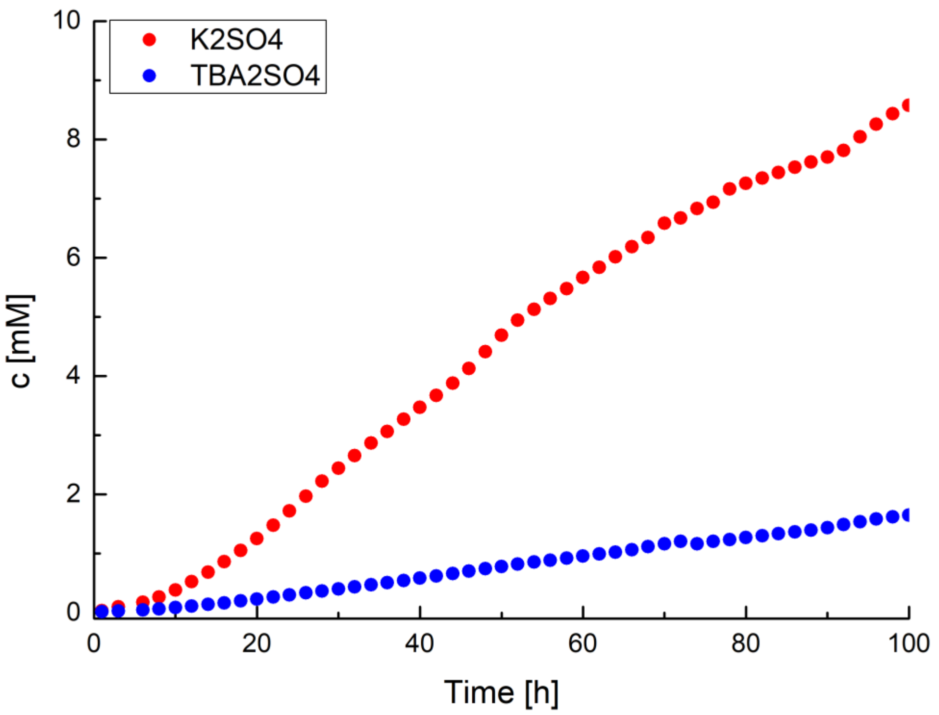

NMR SPECTRA

Fig. S81: ^{1}H NMR spectrum of **S1** in $CDCl_3$.

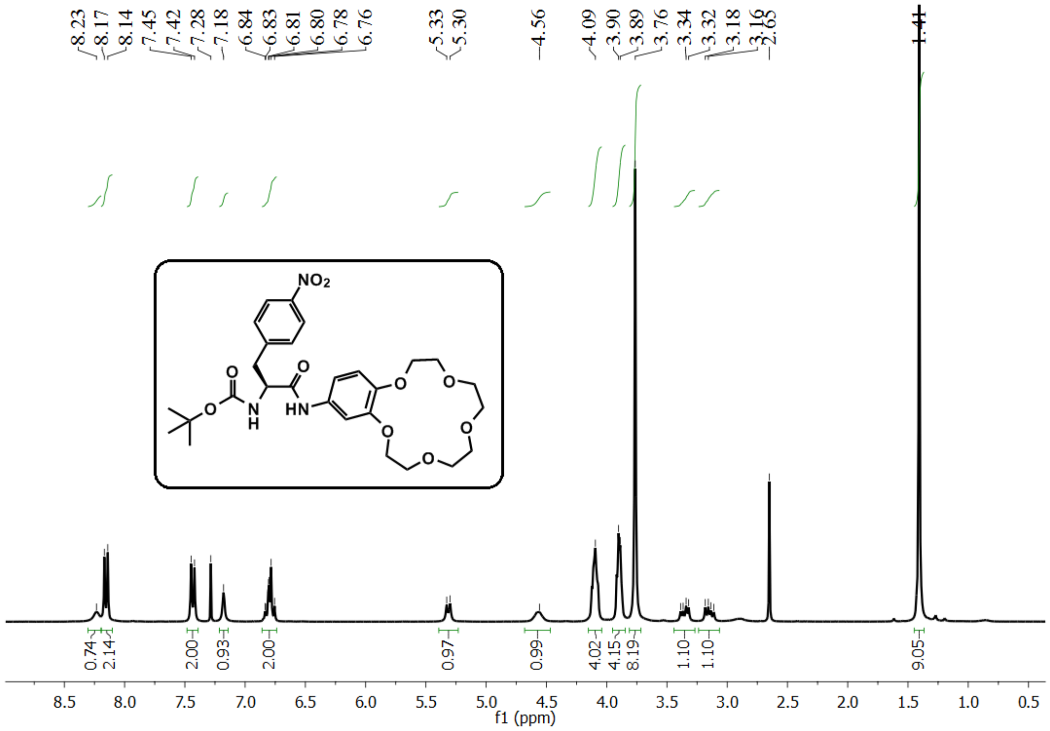

Fig. S82: ^{13}C NMR spectrum of **S1** in $CDCl_3$.

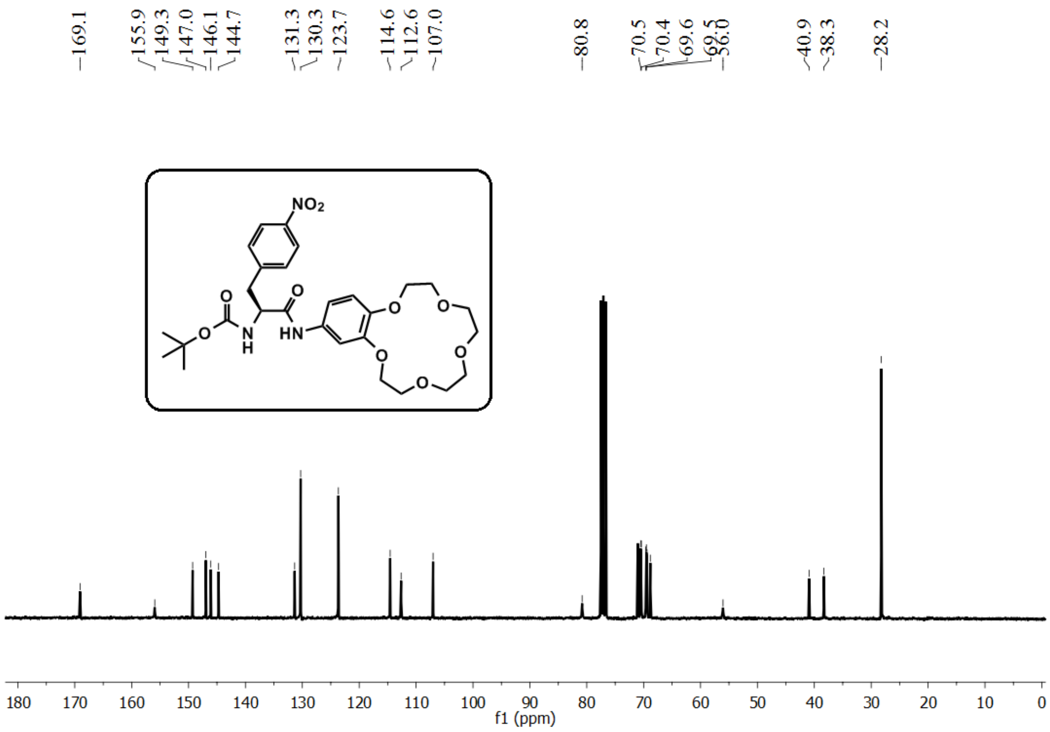

Fig. S83: ^{1}H NMR spectrum of **1** in DMSO-d_6.

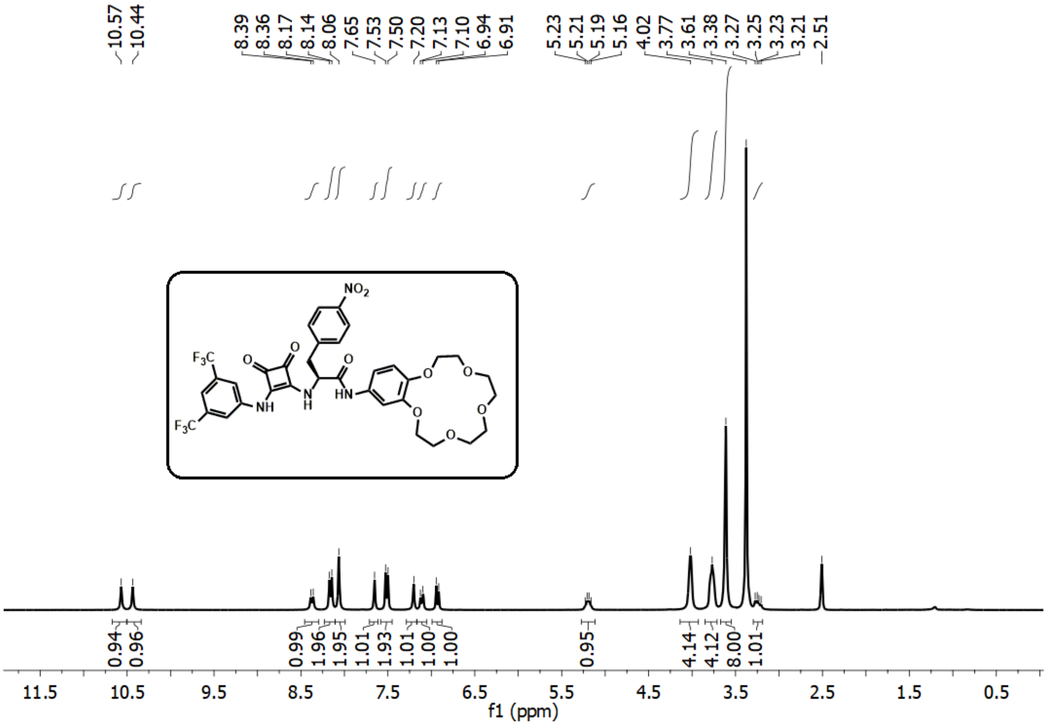

Fig. S84: ^{13}C NMR spectrum of **1** in DMSO-d_6.

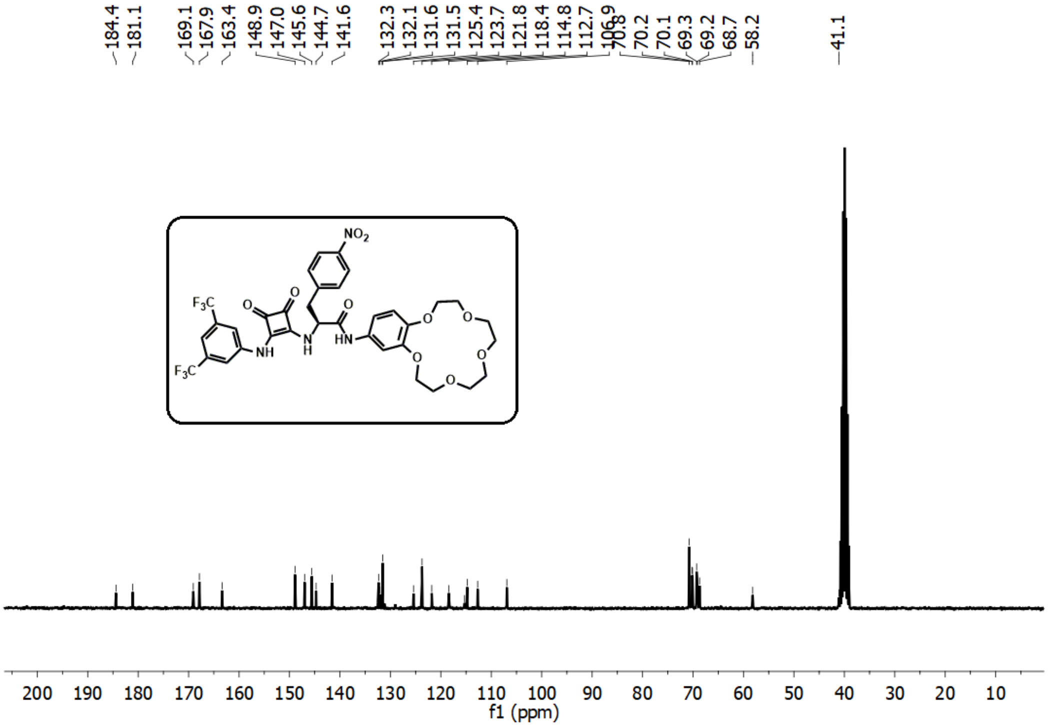

Fig. S85: ^{1}H NMR spectrum of **S4** in DMSO-d_6.

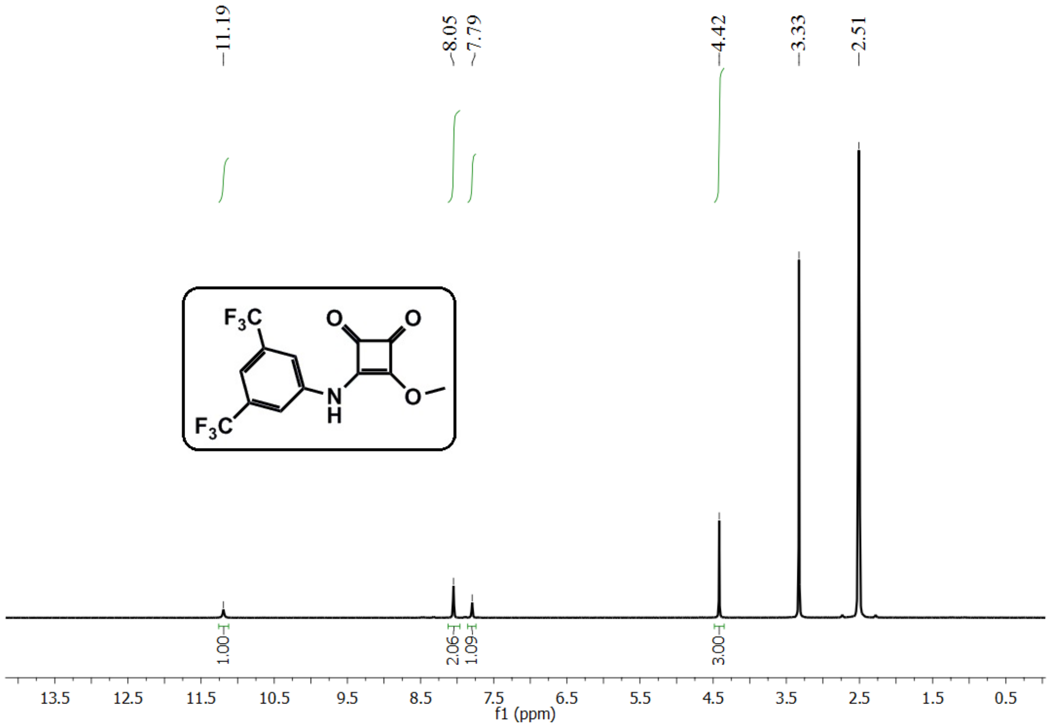

Fig. S86: ^{13}C NMR spectrum of **S4** in DMSO-d_6.

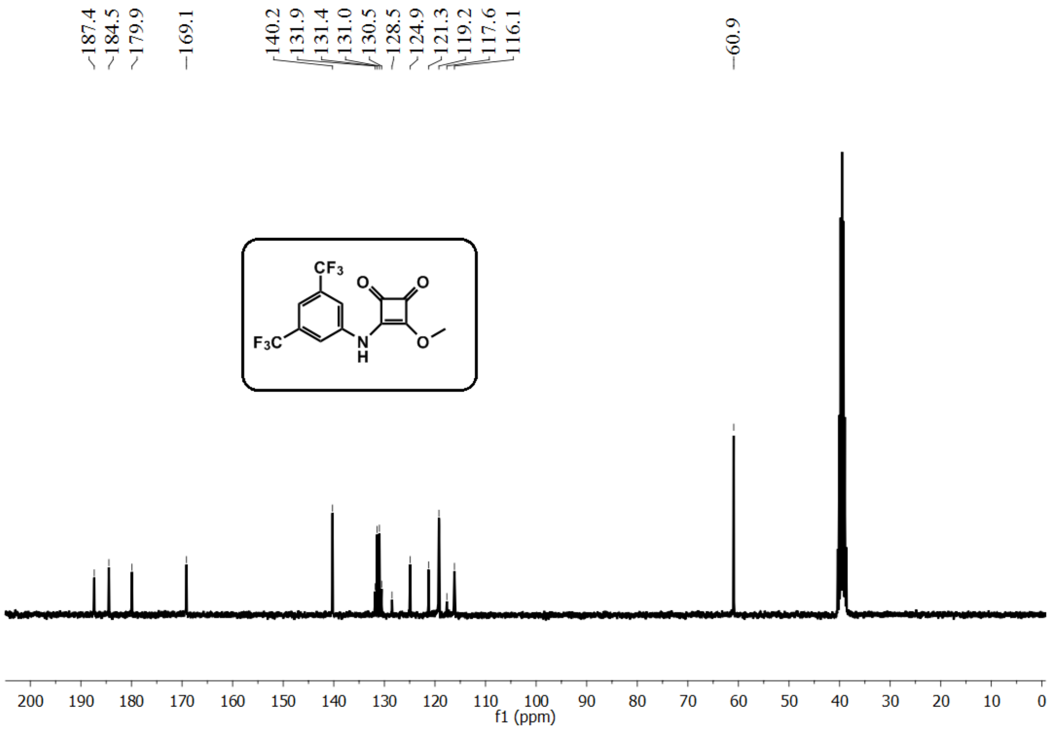

Fig. S87: ^{1}H NMR spectrum of **S2** in $CDCl_3$.

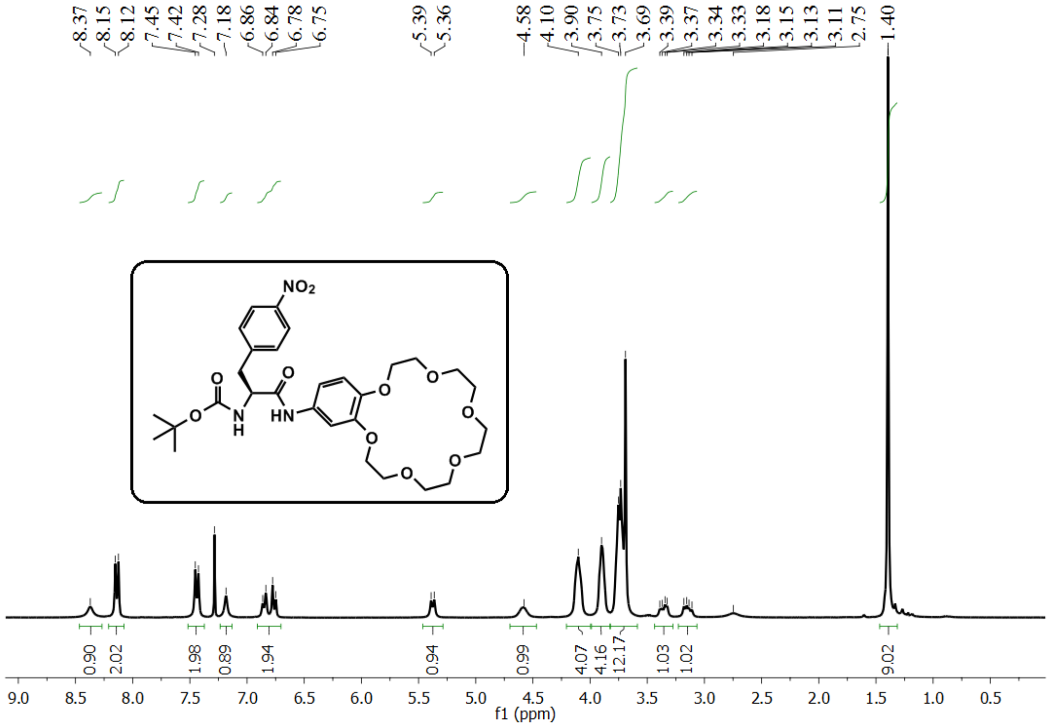

Fig. S88: ^{13}C NMR spectrum of **S2** in $CDCl_3$.

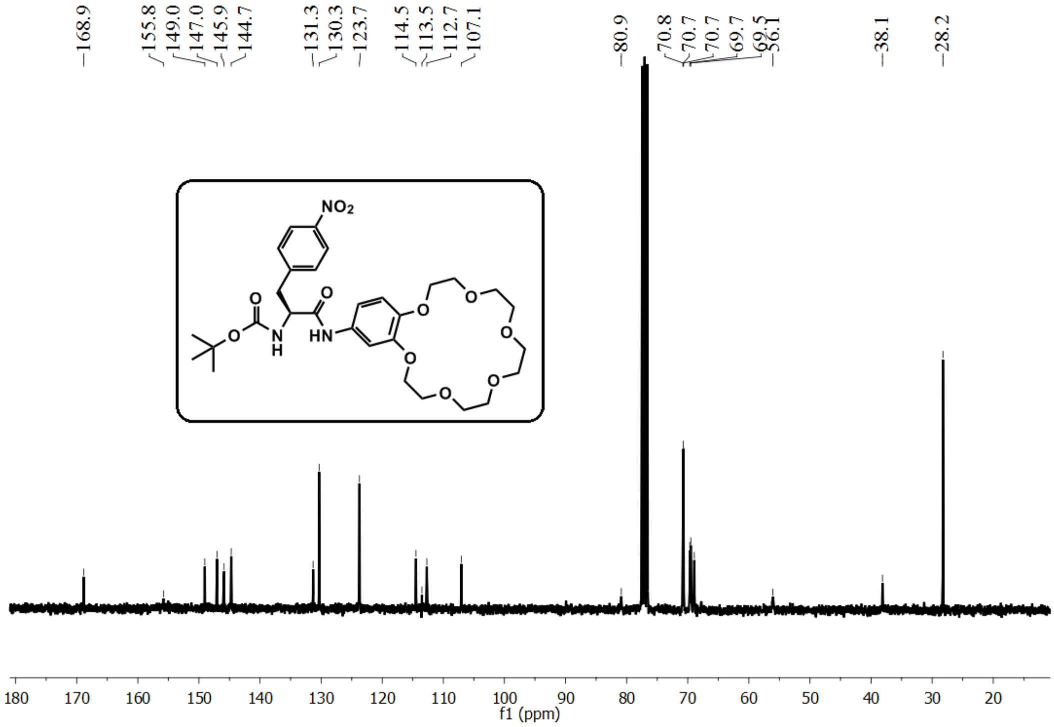

Fig. S89: ^{1}H NMR spectrum of **2** in DMSO-d_6.

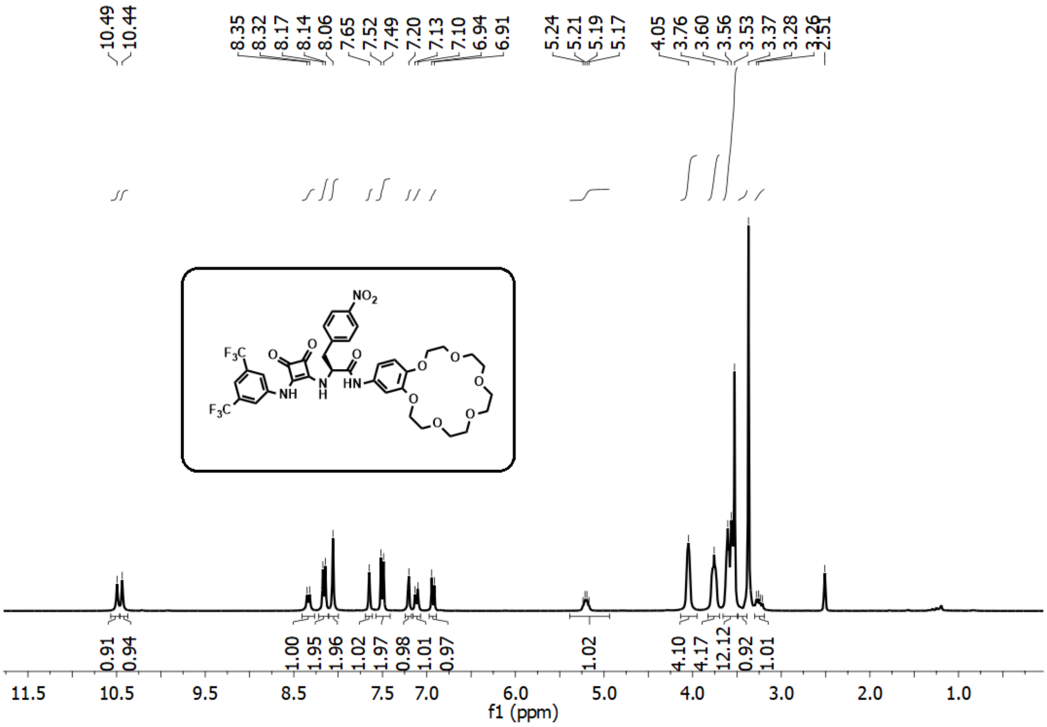

Fig. S90: ^{13}C NMR spectrum of **2** in DMSO-d_6.

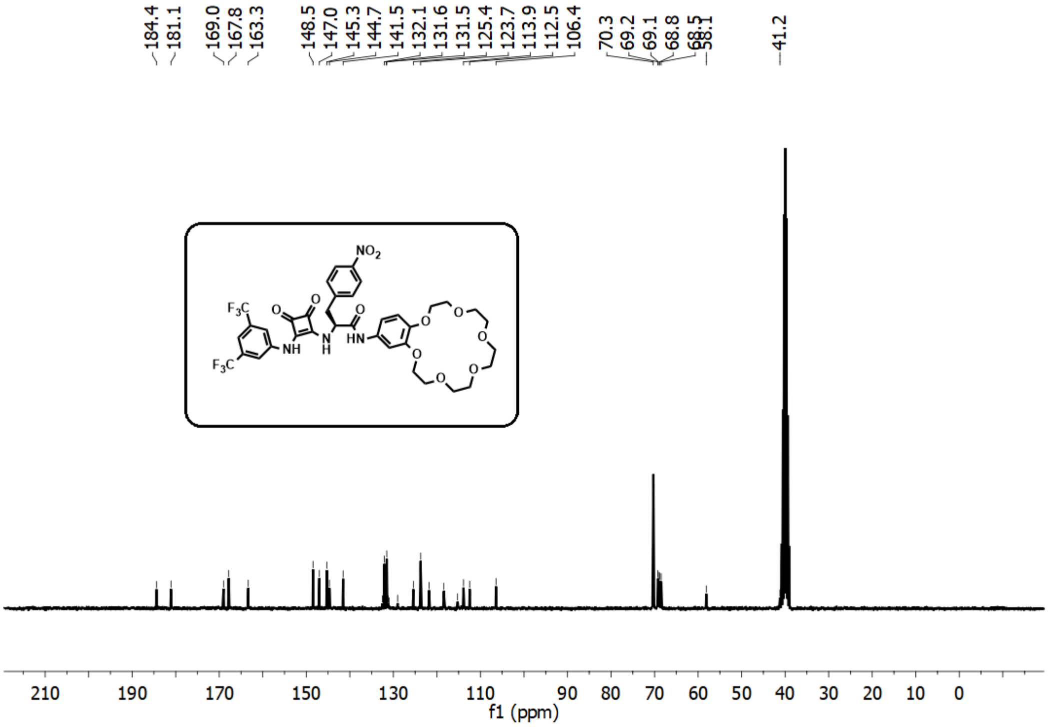

Fig. S91: ^{1}H NMR spectrum of **S3** in DMSO-d_6.

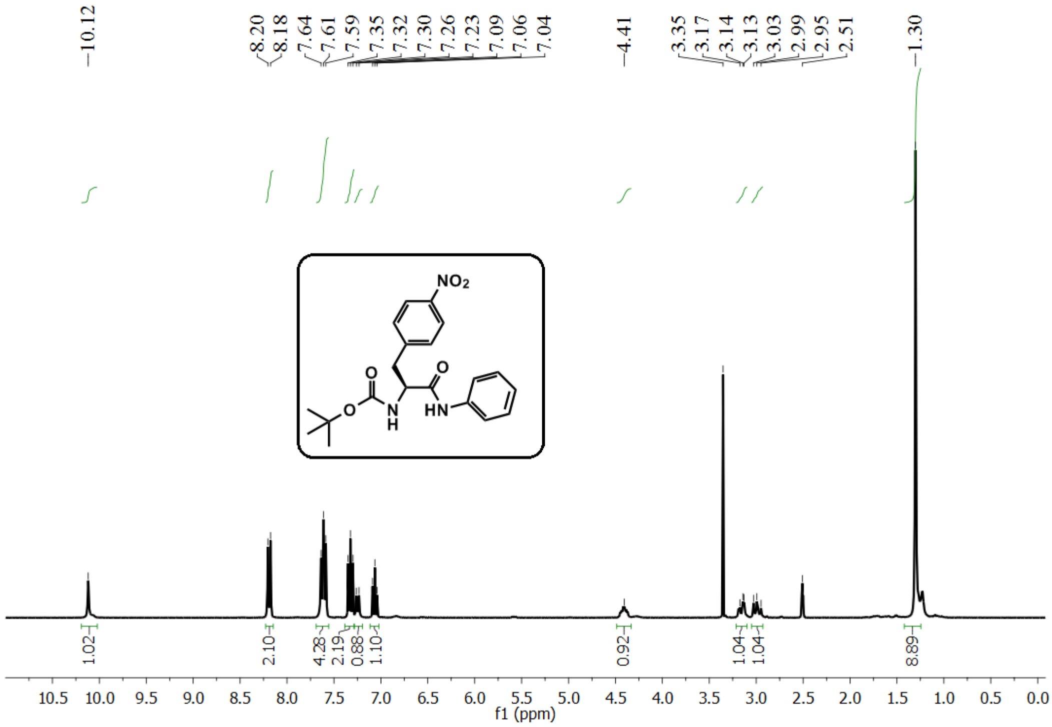

Fig. S92: ^{13}C NMR spectrum of **S3** in DMSO-d_6.

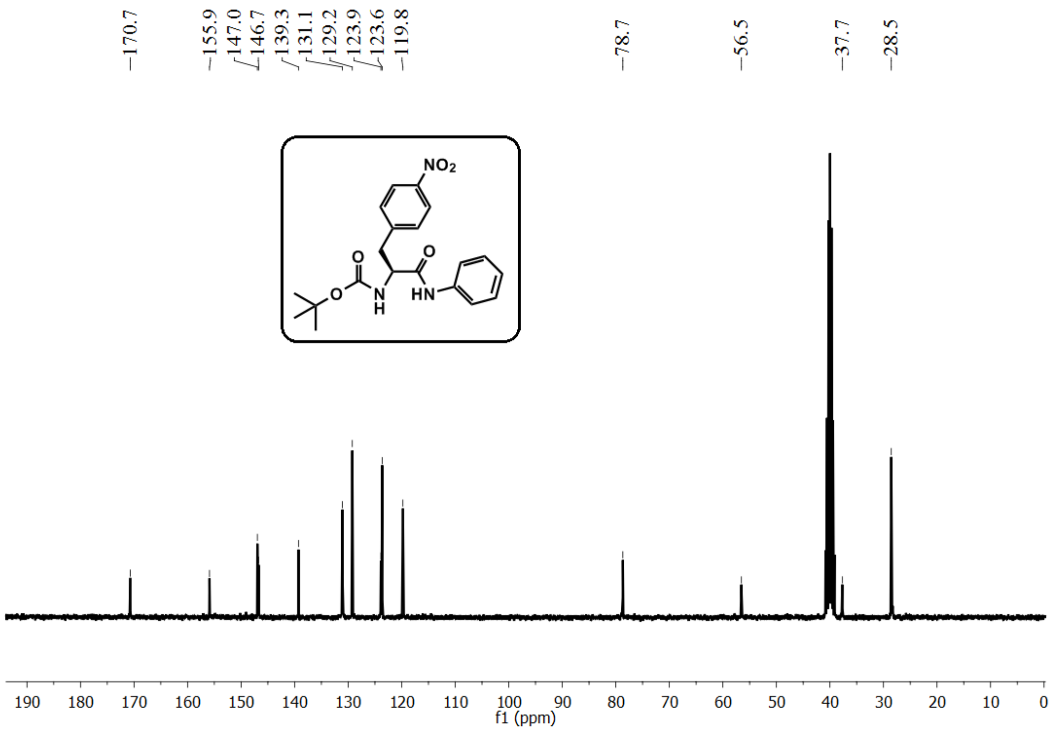

Fig. S93: ^{1}H NMR spectrum of **3** in DMSO-d_6.

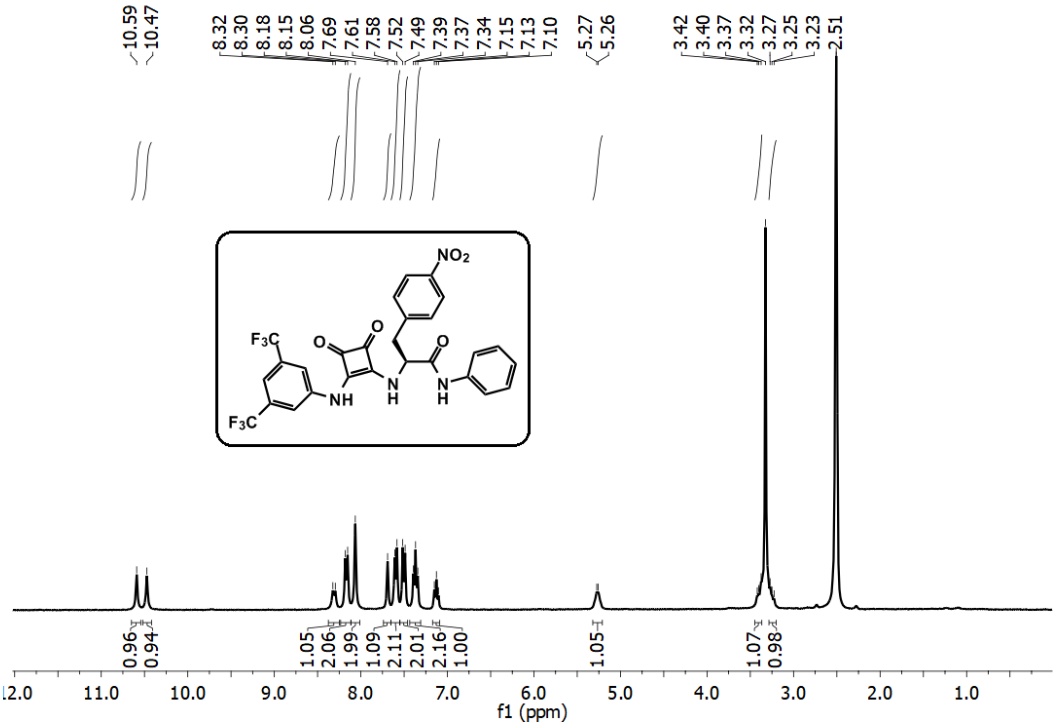

Fig. S94: ^{13}C NMR spectrum of **3** in DMSO-d_6.

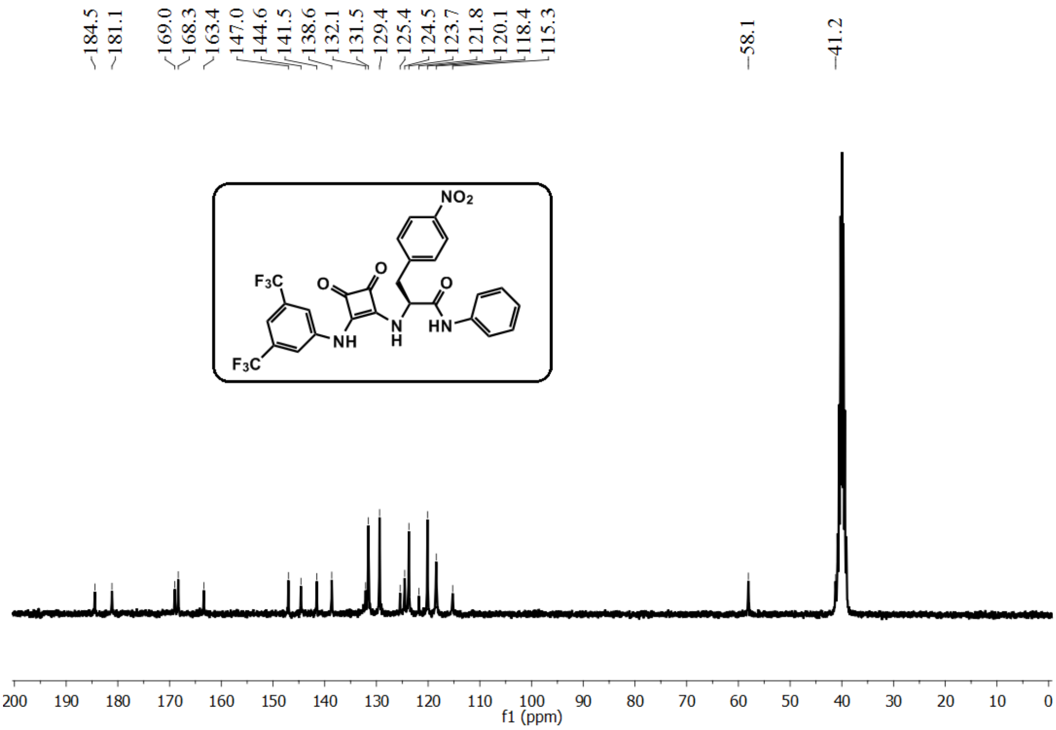

Fig. S95: ^{1}H NMR spectrum of **4** in DMSO-d_6.

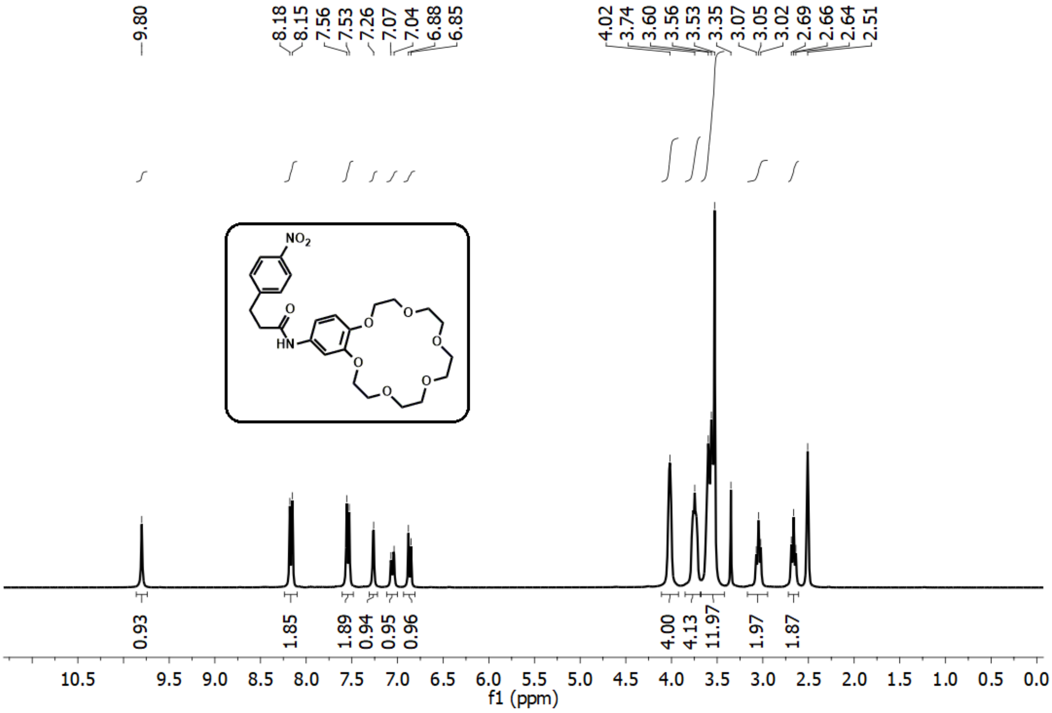

Fig. S96: ^{13}C NMR spectrum of **4** in DMSO-d_6.

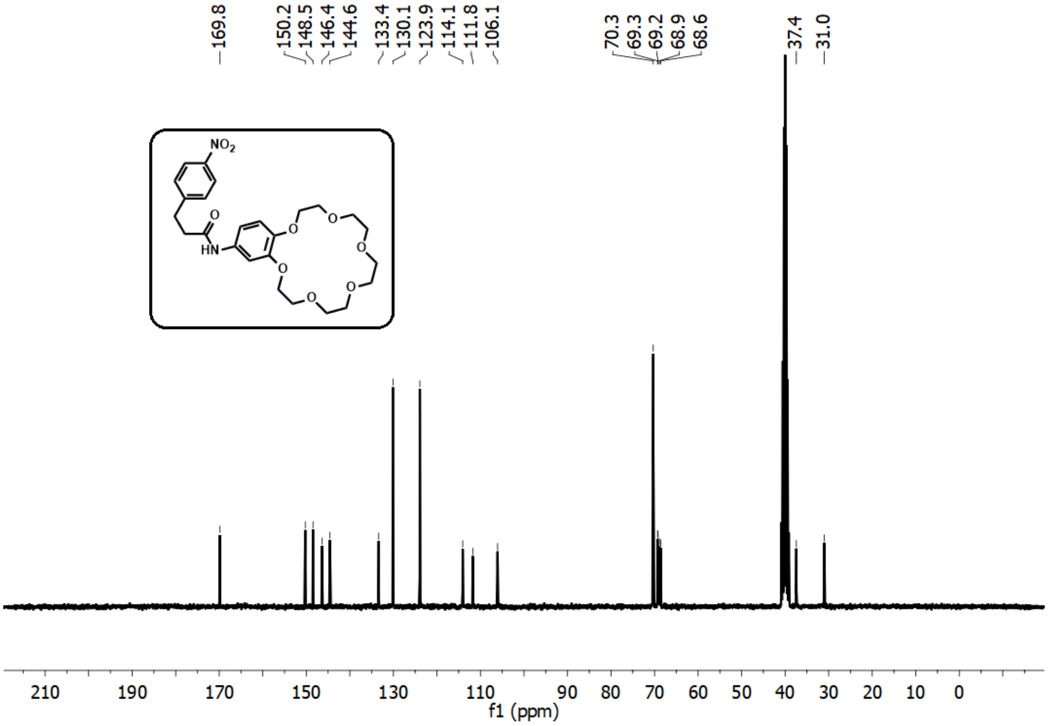

Supplement: Supplementary file 1 [file ijms-22-10754-s001.zip › ijms-1379543-supplementary.pdf]
